# Identification of potent biparatopic antibodies targeting FGFR2 fusion–driven cholangiocarcinoma

**DOI:** 10.1172/JCI182417

**Published:** 2025-02-27

**Authors:** Saireudee Chaturantabut, Sydney Oliver, Dennie T. Frederick, Jiwan J. Kim, Foxy P. Robinson, Alessandro Sinopoli, Tian-Yu Song, Yao He, Yuan-Chen Chang, Diego J. Rodriguez, Liang Chang, Devishi Kesar, Meilani Ching, Ruvimbo Dzvurumi, Adel Atari, Yuen-Yi Tseng, Nabeel Bardeesy, William R. Sellers

**Affiliations:** 1 Broad Institute of MIT and Harvard, Cambridge, Massachusetts, USA.; 2 Dana-Farber Cancer Institute, Boston, Massachusetts, USA.; 3 Harvard Medical School, Boston, Massachusetts, USA.; 4 Faculty of Pharmacy, Silpakorn University, Nakhon Pathom, Thailand.; 5 Ridgeline Discovery GmbH, Basel, Switzerland.; 6 Massachusetts General Hospital Cancer Center, Boston, Massachusetts, USA.

**Keywords:** Oncology, Therapeutics, Drug therapy, Liver cancer, Signal transduction

## Abstract

Translocations involving FGFR2 gene fusions are common in cholangiocarcinoma and predict response to FGFR kinase inhibitors. However, response rates and durability are limited due to the emergence of resistance, typically involving FGFR2 kinase domain mutations, and to suboptimal dosing, relating to drug adverse effects. Here, we develop biparatopic antibodies targeting the FGFR2 extracellular domain (ECD) as candidate therapeutics. Biparatopic antibodies can overcome drawbacks of bivalent monospecific antibodies, which often show poor inhibitory or even agonist activity against oncogenic receptors. We show that oncogenic transformation by FGFR2 fusions requires an intact ECD. Moreover, by systematically generating biparatopic antibodies targeting distinct epitope pairs in FGFR2 ECD, we identified antibodies that effectively block signaling and malignant growth driven by FGFR2 fusions. Importantly, these antibodies demonstrate efficacy in vivo, synergy with FGFR inhibitors, and activity against FGFR2 fusions harboring kinase domain mutations. Thus, we believe that biparatopic antibodies may serve as an innovative treatment option for patients with FGFR2-altered cholangiocarcinoma.

## Abstract 2

We identify biparatopic FGFR2 antibodies that are effective against FGFR2 fusion driven cholangiocarcinoma.

## Graphical abstract

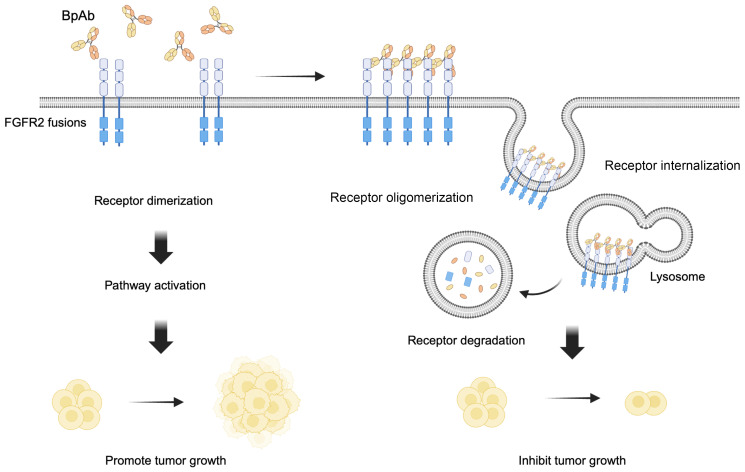

## Introduction

FGFR2 fusions are found across a variety of cancer types including in 10%–15% of primary intrahepatic cholangiocarcinomas (ICCs) (1, 2). While 3 FGFR1-3/4 inhibitors are approved for the treatment of ICC (3), positive trial results are tempered by a short duration of disease control (less than 9 months) and limited response rates (18%–42%) (4). Major challenges of approved FGFR inhibitors include on-target, off-tumor adverse effects and the emergence of resistance mutations, particularly V565 gatekeeper mutations (3). On-target hyperphosphatemia, attributable to the role of FGFR1 in phosphate homeostasis, limits optimal dosing of FGFR1-3 inhibitors (5). While the recently developed FGFR2 selective kinase inhibitor, RLY-4008, shows increased response rates, its benefits are not durable (6). Consequently, although FGFR2-fusion–positive ICCs exhibit sustained dependence on FGFR2 signaling, targeting the pathway with kinase inhibitors alone is insufficient to achieve the desired therapeutic benefit.

Therapeutic antibodies against the extracellular domain (ECD) of FGFR2 could serve as complementary treatment modalities to FGFR kinase inhibitors, offering the potential for high specificity and retaining efficacy in the setting of kinase domain mutations. Importantly, the ECD is retained in all cases of intracellular fusion events. Thus, the FGFR2 ECD may be amenable to antibody-mediated targeting, although there are key questions and hurdles to address to ensure optimal therapeutic development.

One such question is the uncertainty of whether ligand activation contributes to the transforming capacity of FGFR2 fusions, which has important implications for antibody design. In this regard, antibodies to receptor tyrosine kinases (RTKs) can potentially function by blocking signaling as well as through antibody-dependent cellular cytotoxicity (ADCC) or through cytotoxic payloads (7–9). However, bivalent antibodies against RTKs are often only marginally effective inhibitors of signaling and instead often act through ADCC or antibody-drug conjugate payloads (ADCs) (7–9). Indeed, of currently approved antibodies in cancer, less than 10% exhibit signaling pathway blockade, with over 60% exerting immune effector functions and over 25% classified as ADCs (10). Furthermore, receptor targeting by some monospecific (monoparatopic) antibodies lead to agonistic activity due to receptor dimerization and activation (11–14). These data suggest that improvements in the activity of traditional monospecific bivalent antibodies could lead to more effective therapeutic antibodies. As a result, distinct antibody formats have been explored.

Here, we developed biparatopic antibodies targeting of FGFR2 fusions in ICC. First, we defined the contributions of the FGFR2 ECD to transformation by FGFR2 fusion alleles. Second, we generated biparatopic antibodies targeting the FGFR2 ECD. Biparatopic antibodies, which recognize 2 distinct epitopes on the same protein, are a promising format that can produce highly potent antagonists (15–17). By generating all 15 possible combinatorial heterodimeric biparatopic antibodies from 6 optimized monospecific antibodies that bind to distinct epitopes along the FGFR2 ECD, we identified 2 anti-FGFR2 biparatopic antibodies that are markedly superior to their parental bivalent antibodies in their potency against FGFR2-fusion driven cancers. Our study highlights the potential of biparatopic antibodies targeting FGFR2 as therapeutic agents.

## Results

### The extracellular domain is necessary for full transformation by FGFR2 fusions.

**Figure 1:**
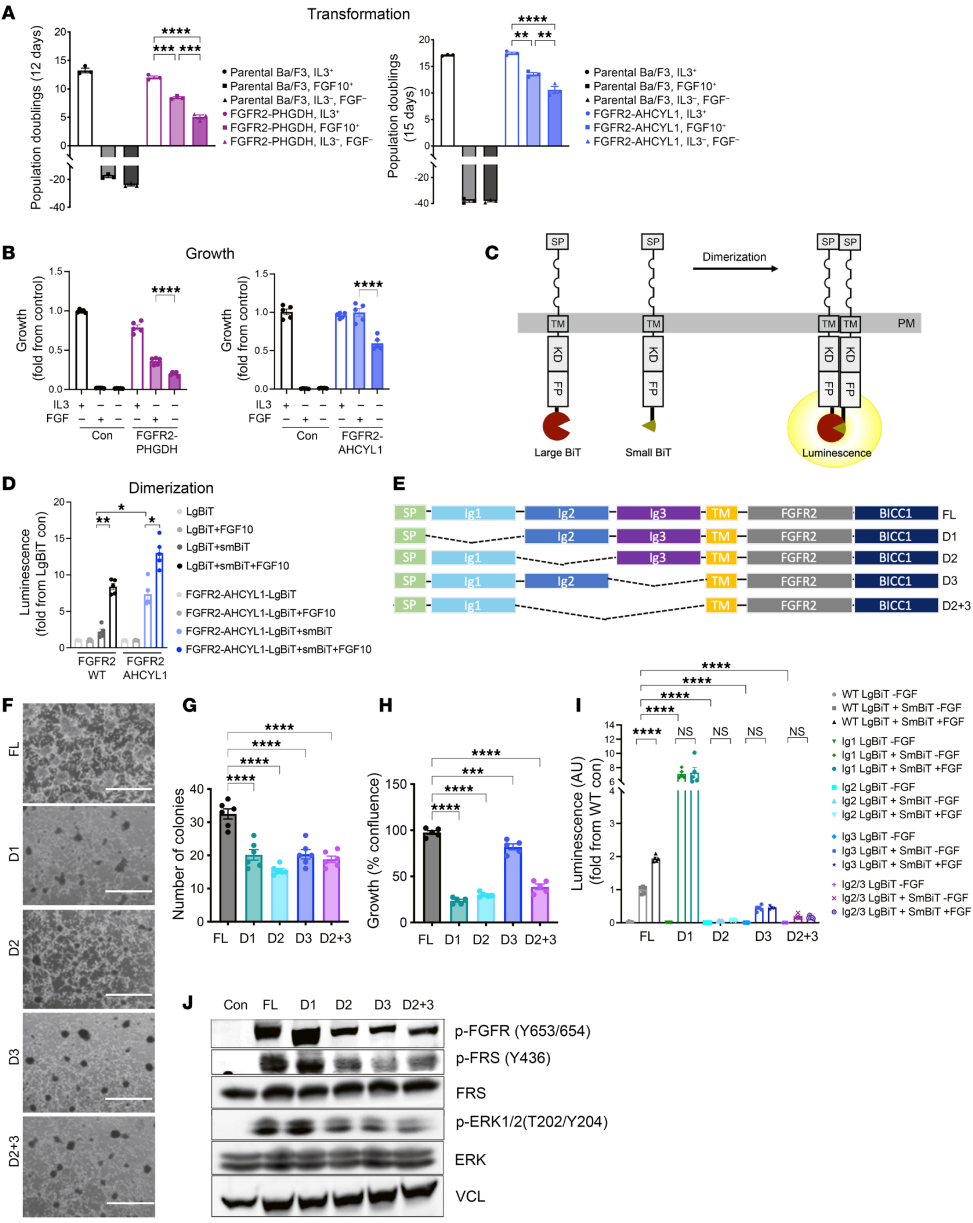
The extracellular domain is necessary for full transformation by FGFR2 fusions. (**A**) Transformation assays showing cumulative population doublings in BaF3 cells expressing FGFR2-PHGDH (12 days) and FGFR2-AHCYL1 (15 days) with or without FGF10 (100 ng/mL) or IL-3 (10 ng/mL), as indicated (*n* = 3). (**B**) Growth of BaF3 cells expressing FGFR2-PHGDH and FGFR2-AHCYL1 analyzed by CellTiter-Glo at 5 days after IL-3 removal (*n* = 5). (**C**) Illustration of the dimerization assay using FGFR2-fusion NanoBiT constructs. Large BiT and Small BiT subunits are fused to the C-terminus of FGFR2 fusions. SP, signal peptide; TM, transmembrane; KD, kinase domain; FP, fusion partner;PM, plasma membrane. (**D**) HEK-293T cells expressing FGFR2-WT and FGFR2-AHCYL1 fused to LgBiT alone or fused to LgBiT and SmBiT were used to quantify the receptor dimerization in the presence or absence of FGF10. Shown is the fold increase over FGFR2-LgBiT activity alone (*n* = 5). (**E**) Illustration of FGFR2-BICC1 constructs with D1 (Ig1), D2 (Ig2), D3 (Ig3), or D2+D3 (Ig2+Ig3) deletions in the ECD. (**F**) Representative images of focus formation assays of NIH-3T3 cells expressing FGFR2 WT or the indicated ECD deletion variants. Scale bar: 250 μm. (**G**) Quantification of number of colonies from **F** (*n* = 6). (**H**) Growth of NIH3T3 cells overexpressing FL, D1, D2, D3, and D2+3–deleted FGFR2-BICC1 constructs as measured by Incucyte at 5 days after plating (*n* = 5). (**I**) Dimerization of FGFR2-BICC1 D1, D2, D3, or D2+D3 ECD–deleted constructs in HEK-293T cells compared with full-length FGFR2-BICC1. Fold change in luminescence over FGFR2-WT–LgBiT is shown (*n* = 5). (**J**) Immunoblotting of FGFR2 downstream pathway effectors in HEK-293 cells expressing FGFR2-BICC1 ECD deletion constructs. All data are mean ± SEM. Data are representative of 1 out of 3 independent experiments. *P < 0.05, **P < 0.01, ***P < 0.001, ****P < 0.0001 by 1-way ANOVA multiple comparisons.

To ascertain the role of FGFR2-fusion ECDs, we developed BaF3 and NIH3T3 fibroblast cell lines expressing FGFR2 fusions: FGFR2-BICC1 (the most common fusion found in ICC), FGFR2-AHCYL1, and FGFR2-PHGDH proteins. Expression of FGFR2 fusions resulted in IL-3–independent growth of BaF3 cells and transformation of NIH3T3 cells (Figure 1A and Supplemental Figure 1A; supplemental material available online with this article; https://doi.org/10.1172/JCI182417DS1); growth of these cells was attenuated by the FGFR inhibitor (FGFRi) infigratinib (Supplemental Figure 1A). Transformation and proliferation of the FGFR2-fusion expressing lines were further enhanced by the FGFR2 ligand FGF10 (Figure 1, A and B). To measure receptor dimerization, we utilized NanoBiT assays that detect protein interactions by proximity-mediated luciferase complementation (18) (Figure 1C). We validated expression of full-length FGFR2-WT and FGFR2-AHCYL1 coupled to the NanoBiT fragments LgBiT and SmBiT (Supplemental Figure 1, B and C) and assayed luminescent activity upon coexpression. Complementation-based luciferase activity of FGFR2 fusions was significantly higher than that of FGFR2-WT (Figure 1D), indicating ligand-independent dimerization. Nonetheless, addition of FGF10 significantly enhanced receptor dimerization of FGFR2-WT and FGFR2-AHCYL1 (Figure 1D). These data indicate that the FGFR2-fusion ECD is functional and enhances fusion receptor activation through ligand-mediated dimerization.

Next, we asked whether subdomains of the ECD were required for FGFR2-fusion dimerization, cell growth, and transformation. To this end, we generated FGFR2 fusions with deletions of the D1, D2, and D3 subdomains (Figure 1E). Since the D2 and D3 domains are necessary and sufficient for ligand binding, we also generated D2 + D3 deletion constructs. Each ECD deletion was expressed in NIH3T3 cells lacking endogenous FGFR2, and we performed colony formation and proliferation assays. Comparable expression of each construct was observed via immunoblotting (Supplemental Figure 1, D and E). D1, D2, D3, and D2 + D3 deletions each reduced growth (35%–77% growth inhibition) and transformation capacity (36%–50% reduction) compared with full length (FL) FGFR2-fusion expressing cells (Figure 1, F–H). Specifically, deletion of D2 of the FGFR2 ECD had a pronounced impact on cell growth and transformation, suggesting that D2 may play a prominent role in the oncogenicity of FGFR2-BICC1. Thus, the ECD is required for full transformation by FGFR2 fusions.

Signaling by FGFR2-WT is initiated by binding of FGF ligands to the D2 and D3 domains leading to receptor dimerization and activation. To test the domain requirement for activity of FGFR2 fusions, we utilized NanoBiT complementation and immunoblotting assays. The D2-, D3-, and D2 + D3–deleted FGFR2 fusions showed significantly impaired dimerization in the presence or absence of FGF10 ligand (Figure 1I). In keeping with the autoinhibitory function of the D1 domain (19), loss of the D1 domain enhanced receptor dimerization. Finally, we assessed the downstream pathway activation of the ECD deletion constructs by immunoblotting. Compared with the FL construct, expression of the D2, D3, and D2 + D3 deletion derivatives showed markedly impaired FGFR2 signaling (reduced p-FGFR (Y653/654), p-FRS2(Y436), and p-ERK(T202/Y204)), whereas the D1 deletion increased FGFR2 signaling output correlating with the observed increase in dimerization (Figure 1, I and J, and Supplemental Figure 1F). Together, these data demonstrate that the FGFR2-fusion ECD is necessary for full transformation of FGFR2 fusions. We further identify an autoinhibitory function of the D1 domain, deletion of which activates ERK leading to diminished viability, consistent with previous observations of activation-dependent lethality we and others observed in BRAF and NRAS mutant setting (20, 21).

### Development of candidate biparatopic antibodies directed against FGFR2.

**Figure 2:**
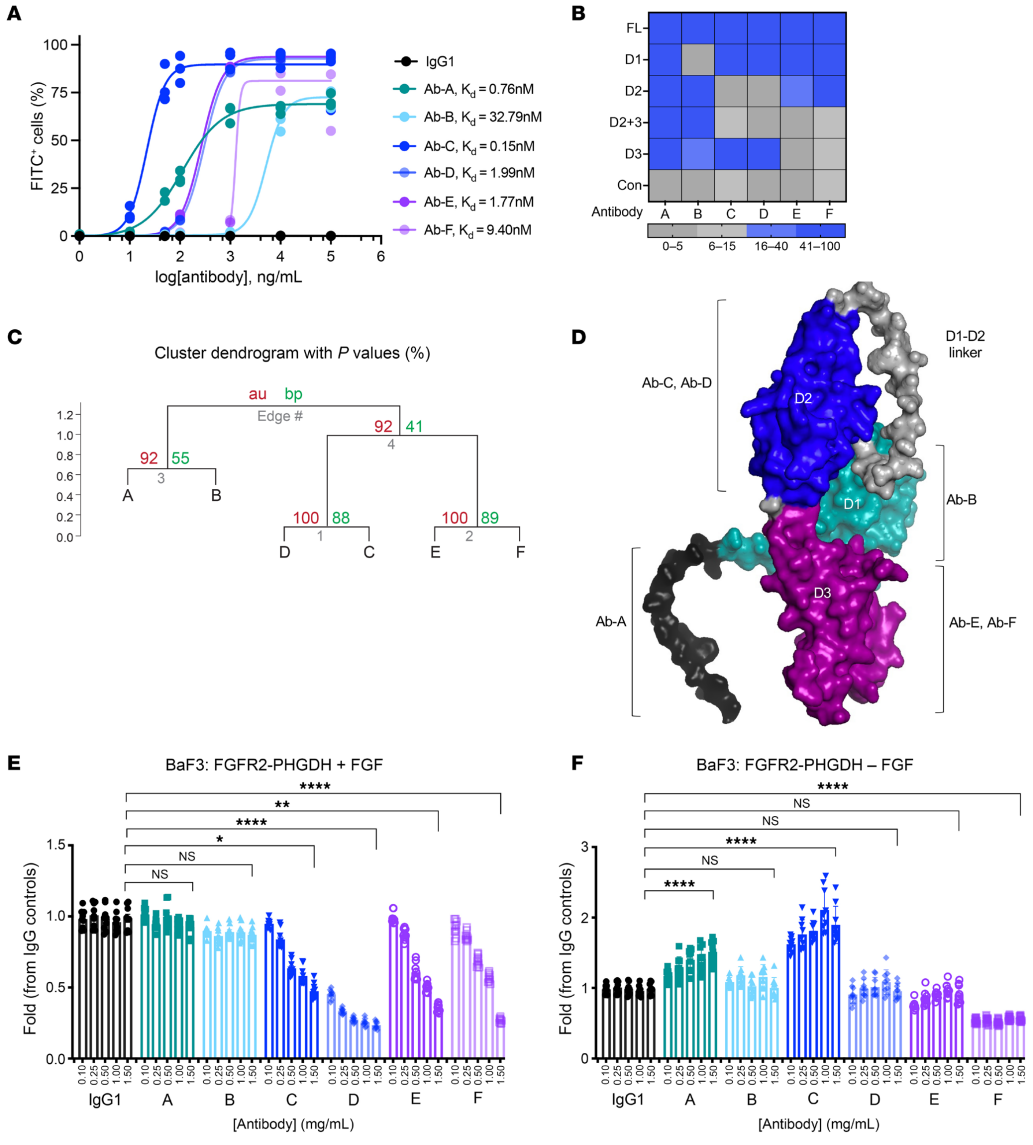
Development of candidate biparatopic antibodies directed against FGFR2. (**A**) Anti-FGFR2 antibodies (Ab-A, Ab-B, Ab-C, Ab-D, Ab-E, and Ab-F) binding to SNU16 cells (FGFR2 amplification) by flow cytometry and their associated apparent Kd values. Anti-hIgG1-FITC secondary antibody was used to detect FGFR2 parental antibodies A–F (*n* = 3). (**B**) Flow cytometry analysis using anti-hIgG1-FITC secondary antibody to detect FGFR2 parental antibodies A–F. Binding epitopes of parental antibodies A–F along the FGFR2 ECD were identified using full-length, D1, D2, D3, and D2+3–deleted FGFR2-BICC1 overexpressing NIH3T3 cell lines shown in Figure 1. (**C**) Epitope binning through cross competition assay. BLI-Octet Epitope clustering diagrams showing cluster dendrogram with au (approximately unbiased) *P* values and bp (bootstrap probability) value (%). Distance represents correlations and cluster method is average. (**D**) α-fold predicted structure of FGFR2 ECD showing D1, D2, D3, and D1-D2 flexible linker as well as 6 FGFR2 parental antibody binding epitopes A–F. (**E** and **F**) Viability of FGFR2-PHGDH–overexpressing BaF3 cells upon treatment with increasing concentrations of antibody A–F in the presence or absence of FGF10 ligand (*n* = 9). All data are mean ± SEM. Data are representative of 1 out of 2 independent experiments. *P < 0.05, **P < 0.01, ***P < 0.001, ****P < 0.0001 by 1-way ANOVA multiple comparisons.

To determine whether biparatopic antibodies can disrupt the function of FGFR2 fusions, we identified and produced 6 optimized FGFR2 antibodies (22–25), including the parental antibody of bemarituzumab, an ADCC-enhanced FGFR2 antibody in phase III trials (26). Available data suggested these antibodies likely bind to distinct epitopes in the ECD of FGFR2b, the primary isoform of FGFR2 fusions expressed in ICC (3). We compared and validated the reported binding epitopes and binding affinities, ascertaining FGFR2 binding by flow cytometry and bio-layer interferometry (BLI) octet analysis. We determined the apparent binding affinities of parental antibodies A–F, finding equilibrium dissociation constants (Kd) ranging from 0.15 nM–32.79 nM (Figure 2A). To validate their binding epitopes, NIH3T3 cells expressing FGFR2-fusion constructs with deletions in D1, D2, D3, or D2 + D3 (Figure 1E) were analyzed by flow cytometry The data showed that antibody A bound to all constructs, antibody B bound to all except the D1-deleted construct, antibodies C and D bound to all but the D2-deleted construct, and antibodies E and F bound to all except the D3-deleted construct (Figure 2B and Supplemental Figure 2A). These data defined the following binding epitopes: antibody B (D1), antibodies C and D (D2), antibodies E and F (D3), and antibody A (outside the D1–D3 domains, likely involving the N-terminus), consistent with prior reports (23). BLI-octet epitope binning analysis by pairwise cross competition corroborated our findings, showing antibodies A and B with unique binding epitopes while antibody C, D and antibody E, F pairs having overlapping epitopes (Figure 2, C and D, and Supplemental Figure 2B).

To determine whether targeting FGFR2-fusion ECDs with anti-FGFR2 antibodies impaired their oncogenic activity, we treated BaF3 cells expressing FGFR2-PHGDH with each FGFR2 antibody. Antibodies against the ligand-binding domain (antibodies C, D, E, and F) inhibited FGF-stimulated growth (Figure 2E), supporting the notion that FGF ligands augment FGFR2-fusion activity and that the ECD is necessary for FGFR2 fusion–driven growth. In the ligand-independent setting, only antibody F inhibited FGFR2-PHGDH–driven BaF3 cell growth (Figure 2F). Antibodies B, D, and E had marginal impacts on cell growth in this setting, while antibodies A and C exhibited agonistic activity and promoted ligand-independent growth (Figure 2F). Consistent with its agonist activity, antibody C increased dimerization of FGFR2-AHCYL1 and FGFR2-BICC1 (Supplemental Figure 2C). As is the case with antibodies against the MET receptor that agonize and dimerize the receptors (14), the ligand-independent growth-promoting effects of antibodies A and C may result from unique binding epitopes eliciting antibody-induced dimerization. In addition, the differential activity of antibodies C and D suggests that they bind to distinct epitopes within the D2 domain.

**Figure 3:**
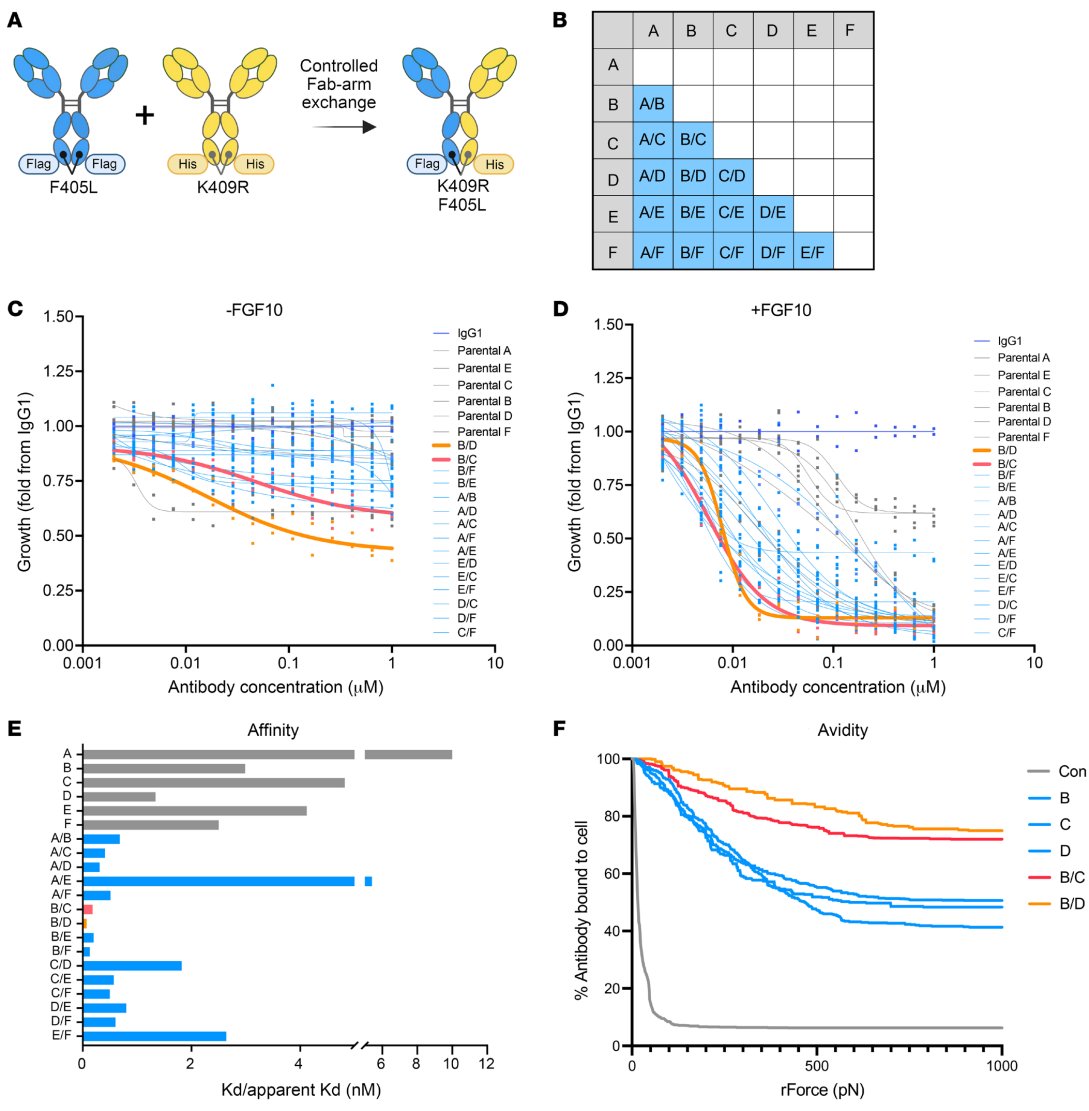
Identification of potent tumor growth–inhibiting biparatopic antibodies via unbiased screening. (**A**) Illustrations showing strategy for biparatopic antibody generation. (**B**) A diagram showing all 15 possible biparatopic antibody pairs that were generated from 6 parental antibodies A–F. (**C** and **D**) Viability of FGFR2-AHCYL1 overexpressing BaF3 cells upon treatment with IgG1, biparatopic antibodies, and their parental antibodies in the absence (**C**) and presence of FGF10 (**D**) (*n* = 2). Data are representative of 1 out of 2 independent experiments. (**E**) Binding affinities (Kd, nM) of parental antibodies (gray) compared with biparatopic antibodies (blue) from MSD-SET assay. Biparatopic antibodies bpAb-B/D and bpAb-B/C showed apparent binding affinities (apparent Kd) of 0.07 nM (orange bar) and 0.18 nM (pink bar), respectively (*n* = 2). Data are representative of 1 independent experiment. (**F**) Representative binding curves illustrating the binding avidity between FGFR2-PHGDH expressing NIH3T3 cells and antibody B, D, C or biparatopic antibody bpAb-B/C and bpAb-B/D via acoustic force spectroscopy (*n* = 4–6). Data are representative of 1 independent experiment.

We next asked whether FGFR2 biparatopic antibodies might have enhanced potency and might avoid ligand-independent agonism. We used controlled Fab-arm exchange to generate full IgG1 FGFR2 antibodies that simultaneously bind 2 different epitopes on the FGFR2 ECD (27). Here, complementary IgG Fc mutations force heterodimer formation between distinct IgG-formatted antibodies while maintaining heavy and light chain pairing. We produced each of the 6 parental antibodies with the reciprocal mutations to create 15 unique biparatopics from all pairwise combinations (Figure 3, A and B). In mass spectrometry analysis each biparatopic antibody showed greater than 95% purity with minimal residual parental antibody (as in Supplemental Figure 3, A and B). In all, we validated the binding affinities as well as binding epitopes of the 6 parental antibodies and generated 15 biparatopic antibodies for further characterization.

### Unbiased screening identifies potent, tumor growth–inhibiting biparatopic antibodies.

We next assessed antiproliferative activity in FGFR2-fusion driven BaF3 cells with or without addition of ligand. Of the 15 biparatopic antibodies tested, 7 (46%) and 11 (73%) outperformed parental antibodies at inhibiting growth of FGFR2-AHCYL1–driven BaF3 cells in the absence or presence of FGF10 ligand, respectively (Figure 3, C and D). A second BaF3 model driven by an FGFR2-PHGDH fusion yielded similar results (Supplemental Figure 3, C and D). Notably, bpAb-B/C and bpAb-B/D were the most potent of the 21 parental and biparatopic antibodies in the viability assays. Importantly, the efficacy of pairwise mixtures of the parental antibodies differed from and did not predict the potency of their respective biparatopic antibodies (Supplemental Figure 3, E and F), suggesting that distinct modes of action are enabled by the biparatopic format.

We next determined the apparent binding affinity of the biparatopic antibodies for FGFR2. Using the MSD-SET assay, we found that 80% (12 out of 15) of biparatopic antibodies, including bpAb-B/C and bpAb-B/D, had marked improvements (greater than 10-fold) in FGFR2 apparent binding affinities compared with their parental antibodies (Figure 3E). The remaining 3 biparatopic antibodies with lower affinities had binding epitopes either within the same ECD subdomain (D2 for bpAb-C/D; D3 for bpAb-E/F) or on subdomains that are the farthest apart (D1 and D3 for bpAb-A/E). These data suggest that the geometry of binding between antibodies and their epitopes plays an important role in achieving high apparent affinity binding. We next determined the binding avidity to FGFR2-expressing cells using acoustic force spectrometry. After binding of antibody-coated beads to FGFR2-PHGDH–expressing NIH3T3 cells on the chip, acoustic force ramp from 0 to 1,000 pN was applied and antibody detachment from cells was observed using real-time fluorescence imaging. bpAb-B/C and bpAb-B/D had markedly enhanced binding avidity compared with parental antibodies B, C, and D, confirming the affinity data (Figure 3F). Finally, we examined the kinetics of antibody association and dissociation using BLI-octet analysis. In addition to their enhanced binding avidity, antibodies bpAb-B/C and bpAb-B/D also exhibited slower off rates and higher apparent affinity (low Kd) compared with their parental antibodies B, C, and D (Supplemental Figure 3, G and H). Both bpAb-B/C and bpAb-B/D contain binding arms against epitope B, a flexible autoinhibitory ECD D1 (Figure 2D). Together, our data demonstrate that the majority of biparatopic antibodies against combinations of selected epitopes on the FGFR2 ECD have enhanced antitumor activity and cellular binding avidity compared with their parental antibodies. Based on these attributes we selected bpAb-B/C and bpAb-B/D for further characterization.

### Biparatopic antibodies show superior inhibition of growth and transformation of FGFR2 fusion driven cholangiocarcinoma cell lines.

**Figure 4:**
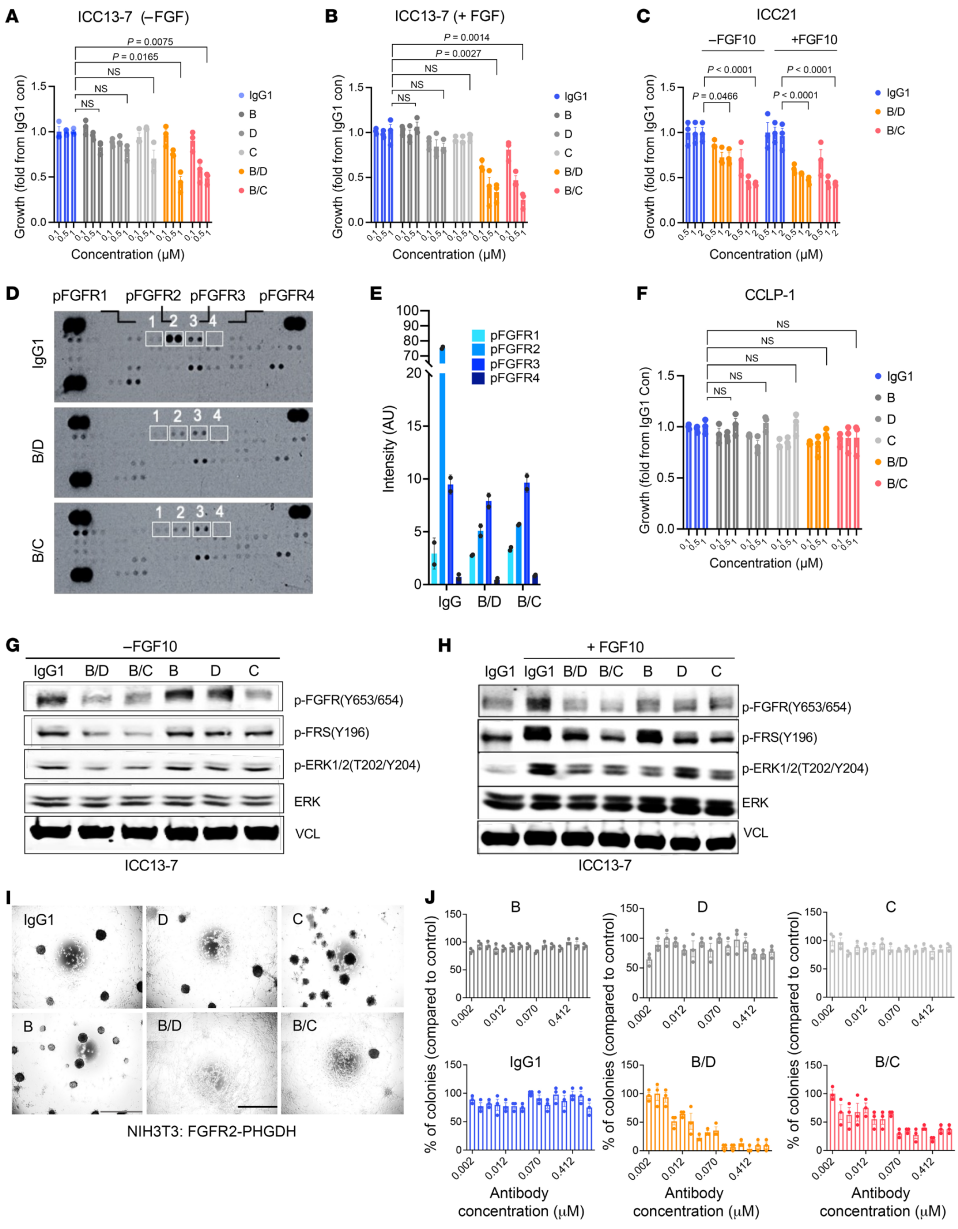
Biparatopic antibodies show superior inhibition of growth and transformation of a FGFR2 fusion–driven cholangiocarcinoma cell line. (**A**–**C**) Viability of cholangiocarcinoma cell line ICC13-7 or ICC21 upon treatment with biparatopic antibodies bpAb-B/C, bpAb-B/D, parental antibodies B, D, C, or IgG1 isotype in the absence (**A** and **C**) or presence (**B** and **C**) of FGF10 at 14 days after seeding (*n* = 3). (**D** and **E**) Proteome profiler human phospho-kinase array demonstrating levels of 43 phosphorylated human kinases in NIH3T3 cells overexpressing FGFR2-PHGDH treated with IgG1, bpAb-B/C, or bpAb-B/D for 5 hours (**D**). (**E**) Quantification of levels of p-FGFR1, p-FGFR2, p-FGFR3, and p-FGFR4 (white boxes) (*n* = 2). (**F**) Viability of CCLP-1 cells upon treatment with biparatopic antibodies bpAb-B/C, bpAb-B/D, parental antibodies B, D, C, or IgG1 isotype control (*n* = 3). (**G** and **H**) Immunoblot of ICC13-7 cells upon 5 hours after treatments with bpAb-B/C, or bpAb-B/D compared to the parental antibodies B, D, C in the absence (**G**) or presence (**H**) of FGF10 ligand. (**I** and **J**) Representative images of focus formation assays of FGFR2-PHGDH–expressing NIH3T3 cells upon treatments with parental antibodies B, D, C, biparatopic antibodies bpAb-B/C and bpAb-B/D, or IgG1 (**I**) as quantified by the number of colonies (**J**) (*n* = 3). Scale bar: 1000 μm. All data are mean ± SEM. Data are representative of 1 out of 2 independent experiments. *P < 0.05, **P < 0.01, ***P < 0.001, ****P < 0.0001 by 1-way ANOVA multiple comparisons.

We investigated the impact of biparatopic FGFR2 antibody candidates bpAb-B/C and bpAb-B/D on 2 patient-derived models of FGFR2 fusion + ICC, ICC13-7 (FGFR inhibitor–sensitive), and ICC21 (partially sensitive) (28). ICC13-7 and ICC21 express the endogenous FGFR2-OPTN and FGFR2-CBX5 fusions, respectively. Correlating with their activity in FGFR2-fusion expressing BaF3 cells, bpAb-B/C and bpAb-B/D have enhanced efficacy at inhibiting growth of ICC13-7 and ICC21 cells in the absence (Figure 4, A and C) and, even greater, in the presence (Figure 4, B and C), of FGF10 compared with the parental antibodies.

To investigate whether cell growth inhibition caused by bpAb-B/C and bpAb-B/D were specific to inhibition of FGFR2 rather than other FGFRs, extracts from NIH3T3 cells expressing FGFR2-PHGDH were profiled using a phospho-RTK array. We found that bpAb-B/C and bpAb-B/D specifically inhibited phosphorylation of FGFR2 but not of FGFR1 or FGFR3 (Figure 4, D and E; minimal FGFR4 phosphorylation was detected in these cells). We also tested FGFR2 specificity using the CCLP-1 ICC cell line, which lacks an FGFR2 fusion and is driven by FGFR1 and FGF20 overexpression (3). Both bpAb-B/C and bpAb-B/D treatments had no significant impact on CCLP-1 cell viability, whereas the IC_50_ for FGFR1-3 inhibitor futibatinib is less than 1.5 nM (3) (Figure 4F). Thus, bpAb-B/C and bpAb-B/D inhibit FGFR2 with high specificity.

We next examined the effects of bpAb-B/C and bpAb-B/D on FGFR2-fusion–mediated signaling. Both bpAb-B/C and bpAb-B/D robustly decreased p-FGFR, p-FRS2, and p-ERK compared with their parental antibodies B, C, or D in a ligand-independent setting (Figure 4G, and Supplemental Figure 4, A, B, and E); additionally, bpAb-B/C and bpAb-B/D blocked FGF10-induced phosphorylation of FGFR, FRS2, and ERK (Figure 4H, and Supplemental Figure 4, A, B, and F). Similarly, bpAb-B/C and bpAb-B/D impaired downstream signaling in NIH3T3 cells expressing FGFR2-PHGDH, including p-FGFR, p-FRS2, p-AKT, and p-ERK (Supplemental Figure 4, C and D). Thus, bpAb-B/C and bpAb-B/D specifically inhibit downstream signaling by constitutively active FGFR2-fusion proteins.

We next assessed the ability of bpAb-B/C and bpAb-B/D to inhibit FGFR2-fusion–driven oncogenic activity via focus formation assays using FGFR2-PHGDH–transformed NIH3T3 fibroblasts (Figure 4I). Cells treated with bpAb-B/C and bpAb-B/D showed a dose-dependent decrease in transformation capacity (reduction in colony formation), whereas the parental antibodies and IgG1-treated control had no effect (Figure 4J). Collectively, these results highlight the specificity of the biparatopic antibodies toward FGFR2 and the marked improvement in the potency of FGFR2 inhibition when compared with bivalent monotopic antibodies.

### Biparatopic antibodies show superior in vivo antitumor activity compared with the parental antibodies.

**Figure 5:**
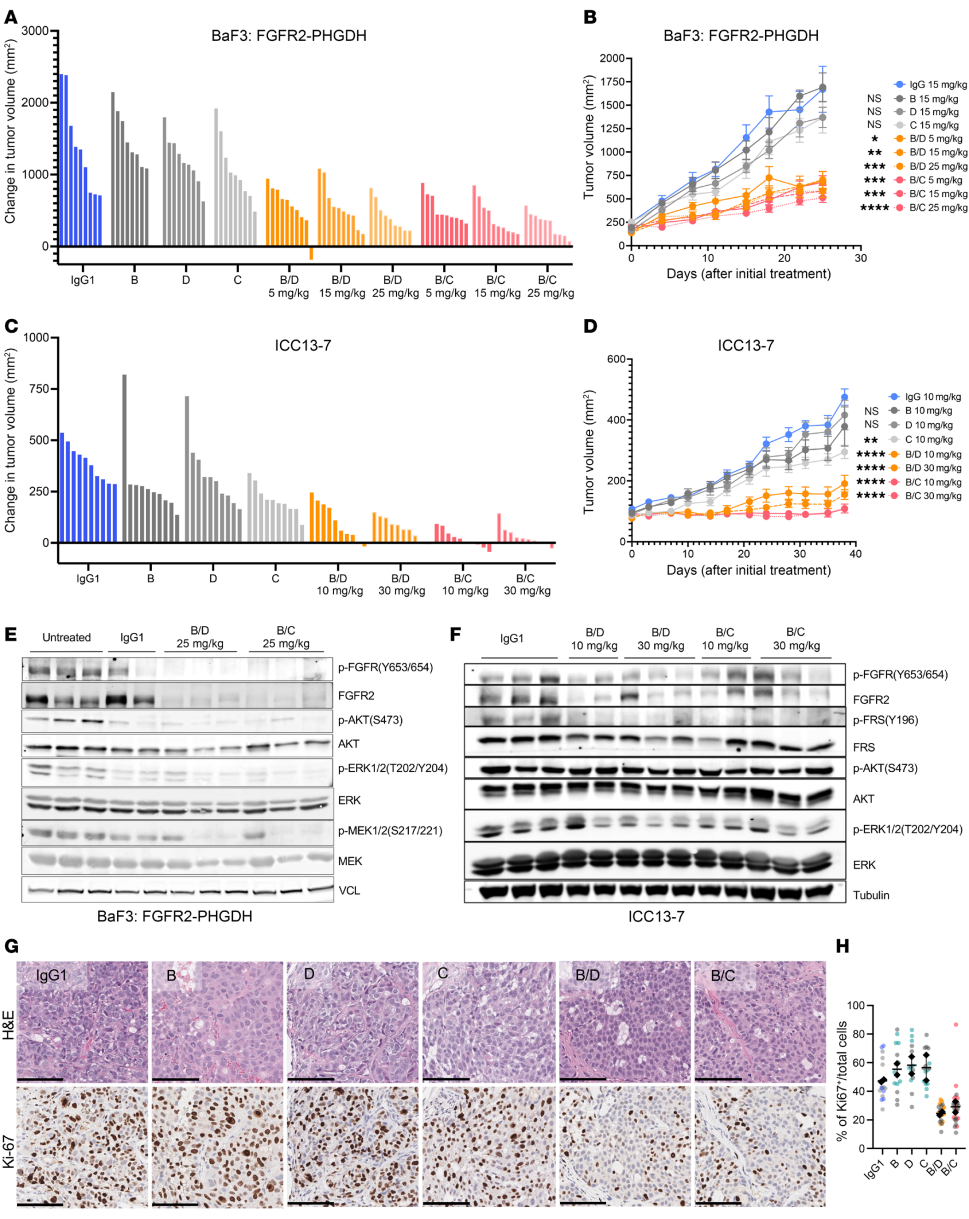
Biparatopic antibodies show superior in vivo antitumor activity compared with the parental antibodies. (**A**–**D**) Tumors of BALB/c scid mice (*n* = 10 per group) harboring BaF3 cells overexpressing FGFR2-PHGDH (**A** and **B**) or ICC13-7 (**C** and **D**) subcutaneous xenografts treated with parental and biparatopic antibodies. Results are represented in the waterfall plot illustrating changes in tumor volume at day 25 (**A** and **B**) or day 38 (**C** and **D**) after initial treatment (**A** and **C**) and as geometric mean of tumor volumes ± SEM every 3–4 days from days 0–25 after initial treatment (**B** and **D**). Data are mean ± SEM across 10 mice. *P < 0.05, **P < 0.01, ***P < 0.001, ****P < 0.0001 by Friedman’s ANOVA multiple comparisons. (**E**) Immunoblot analysis of FGFR2-PHGDH–overexpressing BaF3 cells xenograft tumors harvested 5 hours after the final round of bpAb-B/C, bpAb-B/D, or IgG1 administration at 25 days after initial treatment. (**F**) Immunoblot analysis of ICC13-7 xenograft tumors collected 5 hours after the final round of antibody administration on day 38 after initial treatment. (**G**) Representative images of H&E and IHC staining for proliferation marker Ki-67 in ICC13-7 xenograft tumor samples on the final day of treatment. Scale bars, 100 μm. (**H**) Quantification of the percent of Ki-67–positive nuclei normalized to the total number of nuclei (nuclei counterstain). Data are from 2 biological replicates per treatment group with at least 14 representative images for analysis per group. Data are presented in a superplot where each color represents data points from the same biological sample. Black dots indicate the average values for each biological sample, while black lines represent the overall average for all data points. All data are mean ± SEM. One independent experiment was performed.

We next tested the in vivo efficacy of bpAb-B/C and bpAb-B/D and their parental antibodies against subcutaneous tumors formed by FGFR2-PHGDH–transformed BaF3 cells in SCID mice. At a tumor size of approximately 250mm^3^, mice were randomized into 10 groups with 10 mice per treatment group. The antibodies were administered via intravenous tail vein injections twice per week for 4–6 weeks. Both bpAb-B/C and bpAb-B/D biparatopic antibodies potently suppressed tumor growth at 5, 15, and 25 mg/kg doses, whereas the parental antibodies (administered at 15 mg/kg) showed no antitumor activity (Figure 5, A and B). Pharmacokinetics analysis by ELISA demonstrated dose-proportional increases in the plasma concentration of the biparatopic antibodies, and, furthermore, considerably longer half life compared with small molecule inhibitors, consistent with their larger size (29, 30) (Supplemental Figure 5, A and B).

The biparatopic antibodies also showed prominent in vivo efficacy against xenograft tumors formed by the patient-derived, ICC13-7 cholangiocarcinoma model. While the parental antibodies had only marginal effects on tumor growth, the biparatopics were highly effective at both 10 and 30 mg/kg dose concentrations. Notably, bpAb-B/C showed greatest potency, resulting in tumor stasis at 38 days after treatment (Figure 5, C and D), comparable with the efficacies of clinically used FGFR inhibitors (28, 31). Importantly, bpAb-B/C and bpAb-B/D treatment in both in vivo models led to a marked decrease in total FGFR2 levels and reductions in p-FGFR, p-FRS2, and p-ERK compared with IgG1 control (Figure 5, E and F, and Supplemental Figure 5, C and D). By contrast, the parental antibodies showed limited effect on total FGFR2 levels or on downstream signaling (Supplemental Figure 5, E and F). Consistent with the tumor growth inhibition data, bpAb-B/C and bpAb-B/D markedly decreased tumor cell proliferation (Ki-67 staining) compared with parental antibodies or IgG1 control (Figure 5, G and H). None of the antibody treatments affected mouse body weight (Supplemental Figure 5, G and H). Assessment of antibody tumor distribution by IHC staining showed that bpAb-B/C and bpAb-B/D localized to the cell membrane and exhibited diffuse staining throughout ICC13-7 xenografts (Supplemental Figure 5I), suggesting that biparatopic antibodies penetrate tumor effectively.

To investigate the potential involvement of immune effector functions mediated by biparatopic antibodies in ICC13-7 xenografts, we performed IHC staining for mouse NKp46, a marker for NK cell–mediated antibody dependent cell-mediated cytotoxicity (ADCC) activation (32) and found no significant changes (Supplemental Figure 5, J and K). Similarly, RNA-seq analysis revealed minimal changes in murine gene expression across treatments except for the bpAb-B/C at 10 mg/kg treatment group with only 4 immune-related genes upregulated (Supplemental Figure 5L). We further analyzed the immune system–related gene sets and found no significantly differentially expressed genes observed among treatment groups (Supplemental Figure 5, N–Q). In all cases, tumor growths of matching bpAb-B/C– and bpAb-B/D–treated xenografts were substantially inhibited (Supplemental Figure 5M). Additionally, these antibodies were not potent inducers of NK cell killing of cancer cells (Supplemental Figure 5R), nor robust inducers of NFAT reporters via CD16 (ADC) or CD32a (antibody dependent cellular phagocytosis) in engineered Jurkat cells (Supplemental Figure 5, S and T). Together, these results demonstrate that bpAb-B/C and bpAb-B/D have improved antitumor activity compared with their parental antibodies in vivo, likely driven by receptor downregulation.

### Biparatopic antibodies promote receptor internalization and lysosomal degradation.

**Figure 6:**
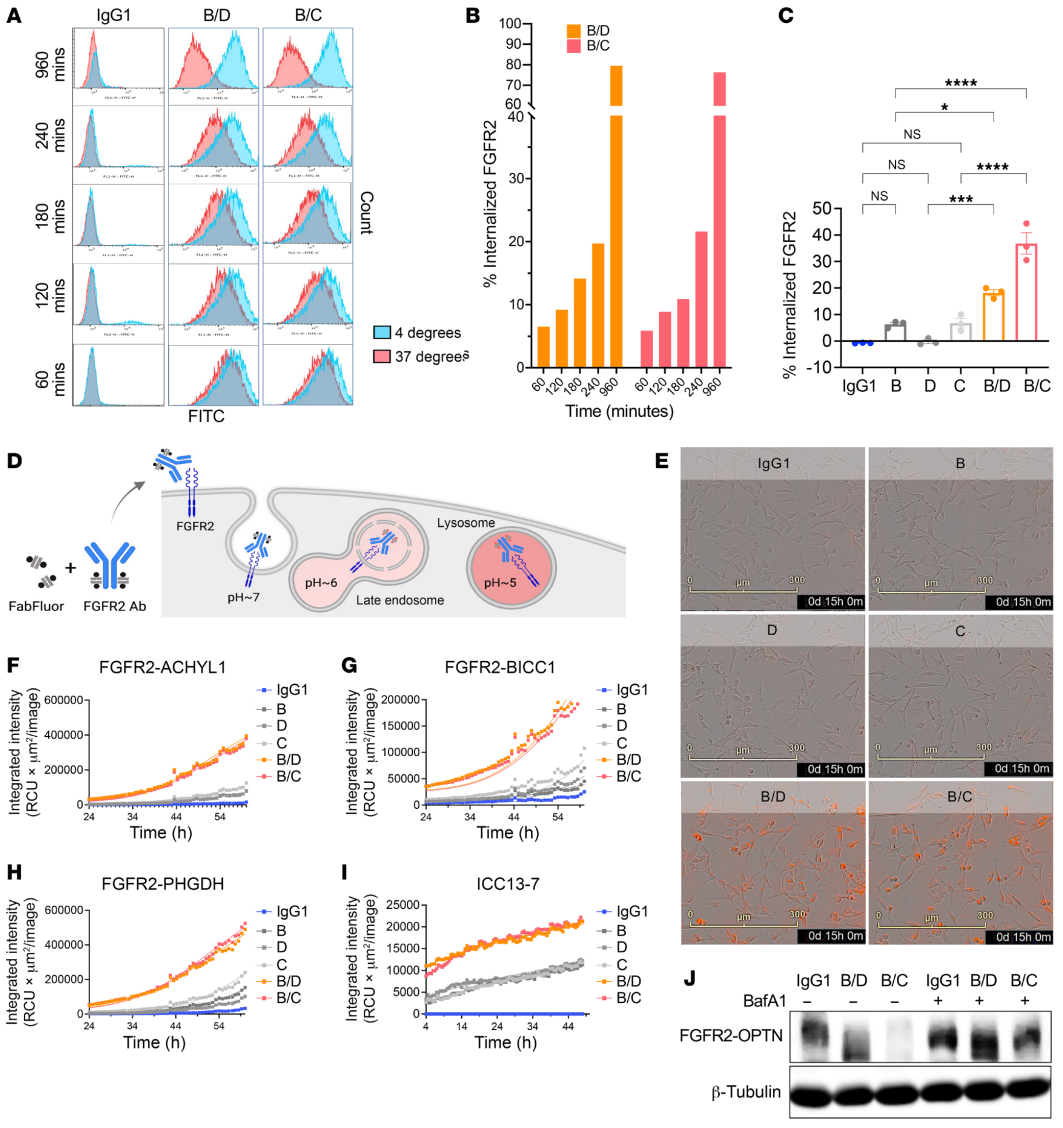
The biparatopic antibodies promote receptor internalization and lysosomal degradation. (**A**) Flow cytometry histograms of surface FGFR2-PHGDH in BaF3 cells at 4°C (blue) and 37°C (red) upon treatment with bpAb-B/C or bpAb-B/D from 60–960 minutes. (**B**) Quantification of the histograms demonstrating the percentage of internalized FGFR2 at 60, 120, 180, 240, and 960 minutes after bpAb-B/C or bpAb-B/D incubation. (**C**) Quantification of histograms showing percent internalized FGFR2 in ICC13-7 cell line at 4°C and 37°C after 5 hours of treatment with parental antibody B, D, C or biparatopic antibodies bpAb-B/C or bpAb-B/D (*n* = 3). Data are mean ± SEM. *P < 0.05, **P < 0.01, ***P < 0.001, ****P < 0.0001 by 1-way ANOVA multiple comparisons. Data are representative of 1 out of 2 independent experiments. (**D**) Illustrations of Fabfluor-pH antibody labeling assay. The pH sensitive dye-based system exploits the acidic environment of the lysosomes to quantify internalization of the labeled antibody. Fluorescent signals that indicate the internalization/degradation events were tracked using Incucyte. (**E**) Representative images of detected fluorophore in NIH3T3 cells expressing FGFR2-PHGDH treated with parental antibody B, D, C, or biparatopic antibody bpAb-B/C and bpAb-B/D at 15 hours after incubation. Scale bars: 300 μm. (**F**–**H**) Quantification of internalization/degradation signals in FGFR2-AHCYL1 (**F**), FGFR2-BICC1 (**G**), and FGFR2-PHGDH (**H**) expressing NIH3T3 cells treated with parental antibodies B, D, C, or biparatopic antibody bpAb-B/C and bpAb-B/D from 24 hours after incubation. Data are representative of 1 out of 2 independent experiments. (**I**) Quantification of internalization/degradation signals in ICC13-7 cells treated with parental antibodies B, D, C, or biparatopic antibody bpAb-B/C and bpAb-B/D at 4 hours after incubation. Data are representative of 1 out of 2 independent experiments. (**J**) Immunoblot of ICC13-7 cells treated with IgG1, bpAb-B/C,or bpAb-B/D antibodies alone or cotreated with bafilomycin A1 (BafA1) for 24 hours. BafA1 was preincubated for 1 hour prior to antibody treatments. Data are representative of 1 independent experiment.

We next explored the potential mechanism for FGFR2 downregulation by the biparatopic antibodies. To determine whether bpAb-B/C and bpAb-B/D promote FGFR2-fusion internalization, we treated FGFR2-PHGDH–expressing BaF3 with bpAb-B/C, bpAb-B/D, or IgG control and then transferred cells to 4°C to block or 37°C to induce internalization. Surface levels of FGFR2 were analyzed by flow cytometry (Figure 6, A and B). Cells treated with bpAb-B/C and bpAb-B/D showed increased internalization from 60 to 960 minutes (from approximately 6% to 80% shift in surface FGFR2) (Figure 6B). The internalization assay was repeated in ICC13-7 cells treated with bpAb-B/C, bpAb-B/D, respective parental antibodies, or IgG control. ICC13-7 cells treated with bpAb-B/C and bpAb-B/D had a significant decrease in surface FGFR2 compared with cells treated with parental antibodies B, C, or D, or IgG1, suggesting that bpAb-B/C and bpAb-B/D enhanced FGFR2 receptor internalization (Figure 6C). Next, we labeled biparatopic and parental antibodies with a Fab fragment conjugated to a pH-sensitive fluorophore (33) and assessed lysosome-mediated induction of fluorescence in FGFR2-PHDGH, FGFR2-AHCYL1, and FGFR2-BICC1–expressing NIH3T3 cells (Figure 6D). Treatment with bpAb-B/C and bpAb-B/D resulted in marked increases in the fluorescent signal compared with the parental antibodies (Figure 6, E–H). Labelling of lysosomes with lysotracker (green) and biparatopic antibodies with Fab-Fluor (red) demonstrated colocalization of the 2 signals, confirming the presence of the antibodies in the lysosomes (Supplemental Figure 6A). Consistent with results in FGFR2 fusion–expressing NIH3T3 cells, treatment of the ICC13-7 cholangiocarcinoma cell line with bpAb-B/C and bpAb-B/D led to increases in fluorescent signals compared with parental antibodies (Figure 6I). In addition, bpAb-B/C and bpAb-B/D showed enhanced receptor internalization and degradation compared with parental antibodies as well as parental antibody mixtures, confirming the unique mechanism of action of biparatopic antibodies beyond antibody combinations (Supplemental Figure 6C).

To investigate whether the observed increase in FGFR2 internalization is triggered by the intermolecular binding of antibodies creating a large complex, as shown in previous work (17, 34), we performed size exclusion chromatography coupled with multiangle light scattering (SEC-MALS), to determine the mass of the antibody and its complexes. Upon increasing the ratio of antigen (FGFR2 ECD) to the biparatopic antibody bpAb-B/C (ECD:Ab) from 1:1, 3:1, and 5:1, SEC-MALS data showed absolute masses consistent with higher-order complexes (Supplemental Figure 6B, see predicted complexes). These results suggest that the bpAb-B/C biparatopic antibodies bind to FGFR2 receptors in trans, likely creating larger antibody-receptor complexes and leading to more rapid internalization.

To determine whether the internalization and receptor downregulation are mediated by lysosomal degradation, we suppressed lysosome acidification and catabolism using the vacuolar-type H+–ATPase inhibitor bafilomycin A1 (BafA1). BafA1 treatment rescued bpAb-B/C- or bpAb-B/D-induced FGFR2-OPTN downregulation in ICC13-7 compared with IgG1-treated control (Figure 6J and Supplemental Figure 6D). Together, these data demonstrate that bpAb-B/C- and bpAb-B/D-induce FGFR2-fusion internalization, trafficking, and lysosomal-mediated degradation to decrease FGFR2 fusion–driven activity and growth. Notably, this mode of action induced by the biparatopic antibodies as shown in our work and others (17, 35–37), does not require cotargeting of lysosome-targeting receptors, membrane E3 ligases, or autophagy signaling molecules, as seen in the development of LYTAC, AbTAC, or AUTAC systems (38).

### Biparatopic antibodies potentiate the efficacy of FGFR inhibitors.

**Figure 7:**
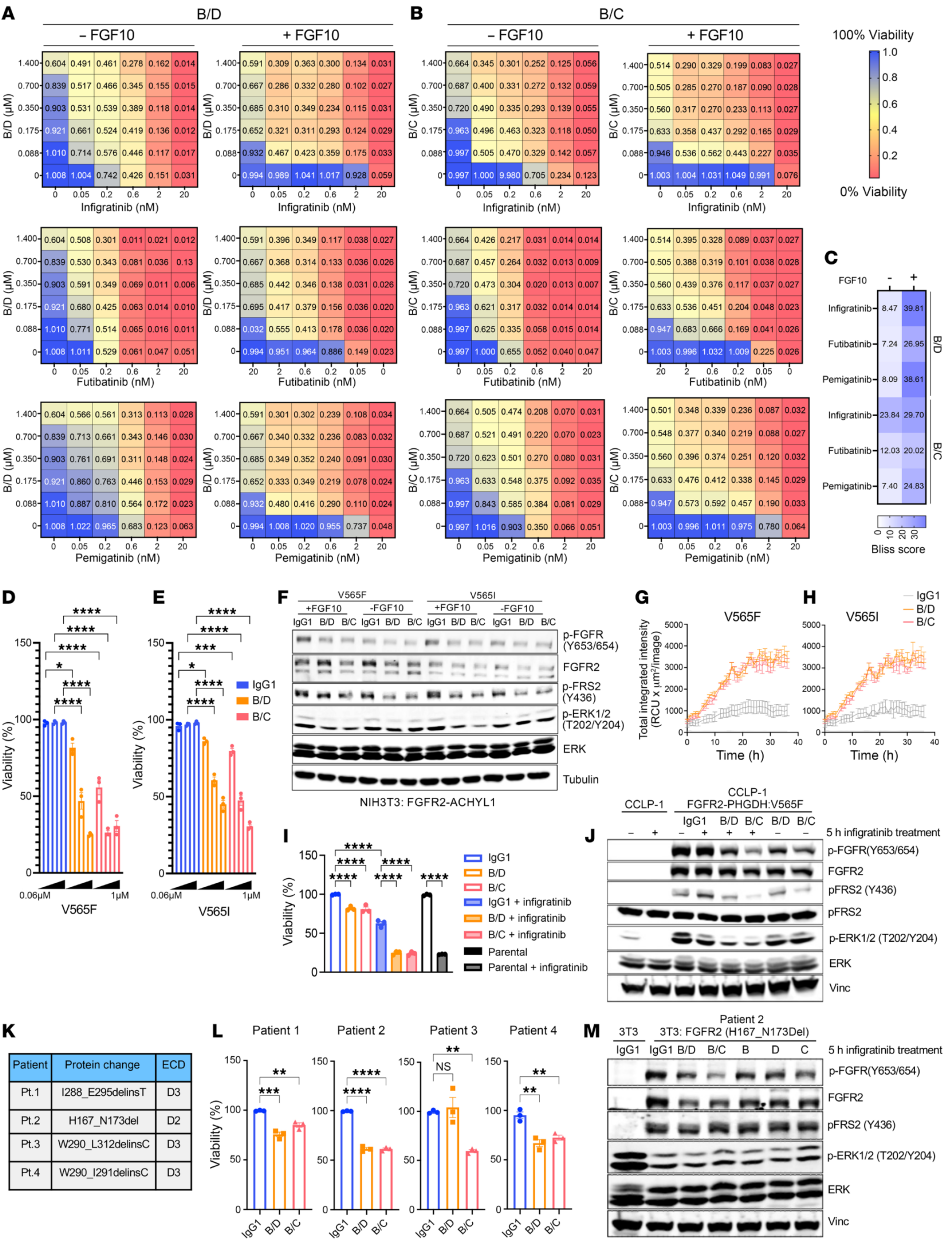
Combinations of biparatopic antibodies with FGFR inhibitors. (**A** and **B**) Biparatopic antibody B/D (**A**) or B/C (**B**) with Infigratinib, Futibatinib, or Pemigatinib combination dose response matrices in the presence of absence of FGF10. 1 = 100% viability and 0= 0% viability after indicated treatment. (**C**) Heatmap showing Bliss scores calculated from dose response matrices using SynergyFinder (39) application for drug combination analysis. (**D** and **E**) Viability of NIH3T3 cells stably expressed FGFR2-AHCYL1 with V565I or V565F mutations treated with bpAb-B/D, bpAb-B/C, or IgG1 (*n* = 3). (**F**) Immunoblot analysis of NIH3T3 cells stably expressing FGFR2-AHCYL1 with V565I or V565F treatment with bpAb-B/D, bpAb-B/C, or IgG1 for 5 hours (*n* = 3). (**G** and **H**) Quantification of internalization/degradation signals in FGFR2-AHCYL1 with V565I or V565F–expressing NIH3T3 cells treated with biparatopic antibody bpAb-B/C, bpAb-B/D, or IgG1 from 0–38 hours after incubation. (**I**) Viability of CCLP-1 cells stably expressed FGFR2–PHGDH fusion with V565F mutation upon treatment with IgG1, bpAb-B/D, or bpAb-B/C alone or in combination with Infigratinib (percentage compared with IgG1 treated control) (*n* = 3). (**J**) Immunoblot analysis of CCLP-1 cell line expressing FGFR2-PHGDH with V565F mutation upon treatment with IgG1, bpAb-B/C, bpAb-B/D, IgG1+Infigratinib, bpAb-B/C + Infigratinib, or bpAb-B/D + Infigratinib for 5 hours. (**K**) Deletion mutations derived from 4 different patients and the respective FGFR2 ECD. (**L**) Viability of 4 patient-derived N-terminus oncogenic mutants upon treatments with IgG1, bpAb-B/C, or bpAb-B/D as indicated (percentage viability compared with IgG1) (*n* = 3). (**M**) Immunoblot of NIH-3T3 cells bearing an FGFR2 H167_N173 in-frame deletion allele (patient 2) after treatment with IgG, bpAb-B/C, bpAb-B/D, or the relevant parental antibodies for 5 hours. All data are mean ± SEM. Data are representative of 2 independent experiments. *P < 0.05, **P < 0.01, ***P < 0.001, ****P < 0.0001 by 1-way ANOVA with multiple comparisons.

Given the specificity of FGFR2 antibodies and the potency of FGFR1-3 kinase inhibitors, combining 2 distinct treatment modalities might result in cooperativity specific to FGFR2 while sparing FGFR1 and 3, leading to more potent FGFR2 inhibition. To test whether bpAb-B/C and bpAb-B/D synergize with FGFRi, FGFR2-PHGDH–expressing BaF3 cells were treated in a titration matrix of bpAb-B/C or bpAb-B/D in combinations with approved FGFRi infigratinib, futibatinib, and pemigatinib. The Bliss model was then applied to determine the degree of synergy (39). Bliss scores of 0–10 generally indicate additive interactions, while scores greater than 10 demonstrate synergistic interactions. In the absence of FGF10, combination of bpAb-B/D with infigratinib, pemigatinib, or futibatinib as well as combination of bpAb-B/C with futibatinib or pemigatinib moderately enhanced growth inhibition (Figure 7, A and B). Synergy between bpAb-B/C and infigratinib in a ligand-independent setting was striking, with a Bliss score of greater than 20 (Figure 7, B and C). In the presence of FGF10, cotreatments of bpAb-B/C or bpAb-B/D with infigratinib, futibatinib, and pemigatinib all enhanced growth suppression compared with treatment with single agents (Figure 7, A–C). In accordance with the dose-response, all Bliss values were well above 10 in the ligand-dependent context (Figure 7C). These data highlight the potential of the biparatopic antibodies to boost the activity of FGFR inhibitors both in the presence and absence of ligand.

Diverse secondary FGFR2 kinase domain mutations drive clinical resistance to each of each FGFR TKI studied to date (3, 40, 41). Given the intracellular location of the kinase domain, we hypothesized that the biparatopic antibodies might remain active against these mutations. To test this hypothesis, we selected the gatekeeper mutations V565I and V565F, which are common mechanisms of resistance to the approved FGFR inhibitors. NIH3T3 cells that stably expressed FGFR2-AHCYL1 with a V565I or V565F mutation were resistant to infigratinib (Supplemental Figure 7A) but were sensitive to bpAb-B/C and bpAb-B/D, showing inhibition of both growth (Figure 7, D and E) and downstream signaling, as evidenced by levels of p-FGFR, p-FRS2, and p-ERK1/2 (Figure 7F and Supplemental Figure 7B). Moreover, bpAb-B/C or bpAb-B/D–induced lysosomal degradation of the FGFR2 fusion in these cells as assayed by anti-Fc Fab fragment conjugated pH-sensitive fluorophore (Figure 7, G and H), similar to that observed in NIH3T3 cells expressing the initial FGFR2 fusions (Figure 6, F–H). Given the complexity of resistance mechanisms in patient tumors, which may implicate multiple oncogenes and bypass mechanisms, we modeled the efficacy of our antibodies in the FGFR1-dependent cholangiocarcinoma cell line, CCLP-1, stably transduced to express the FGFR2-PHGDH-WT or FGFR2-PHGDH-V565F alleles (Supplemental Figure 7, C and D). CCLP-1 parental cells as well as CCLP-1 cells expressing FGFR2-PHGDH WT were sensitive (IC_50_ < 2 nM), while FGFR2-PHGDH V565F cells were resistant (IC_50_ > 2,000 nM) to infigratinib (Supplemental Figure 7E). To determine the dose of infigratinib to use in combination studies (in order to suppress the concurrent FGFR1 activity), we determined the infigratinib concentration that sensitized cells expressing FGFR2-PHGDH-WT but not FGFR2-PHGDH-V565F (0.15 μM). Treatment with bpAb-B/C or bpAb-B/D in combination with infigratinib significantly suppressed growth of V565F resistant mutants and resensitized the CCLP-1 resistant cells to infigratinib, indicating robust suppression of the introduced FGFR2 resistance allele (Figure 7I). In addition, cotreatments of infigratinib and bpAb-B/C or bpAb-B/D decreased levels of FGFR2, p-FGFR, p-FRS2, and p-ERK1/2 (Figure 7J and Supplemental Figure 7F). These results support the use of bpAb-B/C and bpAb-B/D to overcome secondary FGFR2 kinase domain mutations.

In addition to FGFR2 rearrangements, a recent study revealed that activating in-frame FGFR2 ECD deletions occur in approximately 3% of patients with ICC. Patients with these FGFR2 ECD deletions responded well to FGFRi treatments, suggesting that these ECD mutations are oncogenic drivers (42). Since these mutations are located in the ECD, it is possible that they might lack sensitivity to our biparatopic antibodies. To determine whether bpAb-B/C or bpAb-B/D have activity against oncogenic FGFR2 ECD in-frame–deletion mutations, we engineered NIH3T3 cells to stably express 4 patient-derived FGFR2 ECD-deletion mutations (Figure 7K). Compared with NIH3T3 cells expressing FGFR2-WT, cells expressing deletion mutations had increased transformation capacities and receptor dimerization as analyzed by soft-agar assay and NanoBiT assays, respectively (Supplemental Figure 7, G–K). In addition, the ECD mutants had elevated FGFR2 downstream phosphorylation; p-FGFR, p-FRS2, and p-ERK1/2, which was blocked by infigratinib, confirming their FGFR2 dependency (Supplemental Figure 7, L and M). While bpAb-B/C or bpAb-B/D had moderate activities against patient 1– and 3–derived mutants, both bpAb-B/C and bpAb-B/D effectively inhibited growth of patient-2 and -4 variants (Figure 7L). These results correlated with the decrease in levels of FGFR2, p-FGFR, p-FRS2, and p-ERK1/2 for the H167_N173Del (patient 2) variant (Figure 7M and Supplemental Figure 7O). Importantly, levels of FGFR2 decreased upon bpAb-B/C and bpAb-B/D treatments, suggesting that receptor internalization and degradation mediate the observed growth inhibition (Figure 7M and Supplemental Figure 7O). Crucially, mutations found in patients 1–4 are predicted to alter the 3-dimensional structure of FGFR2 D2 and D3 domains (42) and may consequently affect the binding affinities of bpAb-B/C and bpAb-B/D with D1 and D2 binding arms. Nevertheless, the fact that bpAb-B/C and bpAb-B/D remain effective against patient 2 and 4 variants suggest that as long as the binding avidities of D1 and D2 binders are sufficient to establish intermolecular interaction and trigger internalization, the bpAb-B/C and bpAb-B/D should be effective. These data demonstrate that bpAb-B/C and bpAb-B/D have activities against intracellular kinase domain mutations and specific patient-derived FGFR2 ECD oncogenic deletions. Together with the observed synergy, these data support the notion of combining FGFR1-3 inhibitors with FGFR2 biparatopic antibodies.

## Discussion

In this study, we established that the FGFR2 ECD is required for the oncogenic activity of FGFR2 fusions. A series of monospecific antibodies against FGFR2, however, were largely ineffective at blocking downstream signaling. Accordingly, we systematically generated biparatopic antibodies against a diverse combination of epitopes that span 3 domains on the FGFR2 ECD. Through unbiased phenotypic screening using cancer growth inhibition as a functional readout, we selected 2 biparatopic antibody candidates that achieved highest efficacy in vitro and confirmed their therapeutic activities in FGFR2 fusion ICC xenograft models in vivo. The antibodies had synergistic combination activity with FGFR2 TKIs and had activity against gatekeeper kinase mutations as well as N-terminal oncogenic FGFR2 alterations in the ECD. Overall, our work highlights the therapeutic potential of these antibodies in ICC and presents a framework for the development of biparatopic antibodies more broadly.

A variety of modes of action of biparatopic antibodies might contribute to their efficacy. Upon binding to its target, the biparatopic antibody could (a) exert agonistic activity by mimicking the ligand-induced receptor activation (43), (b) act as a true ligand antagonist, blocking the ligand interaction and downstream signaling activation, or (c) induce receptor internalization and degradation through intermolecular crosslinking and complex formation. Critically, only the latter mode of action can inhibit ligand-independent receptor activation and sustainably downregulate signaling pathway to reduce tumor growth. In this work, we have shown mechanistically that the abilities of bpAb-B/C and B/D to effectively inhibit ligand-independent FGFR2 fusion activation are likely mediated through enhanced receptor internalization and lysosome-mediated receptor degradation, which results in tumor growth inhibition in vivo.

Recent advances have been made in the field of targeted protein degradation utilizing endo-lysosomal pathways, such as lysosome-targeting chimeras (LYTACs) and antibody-based PROTAC (AbTAC) platforms. Despite their promises for eliminating soluble proteins, the success of these platforms at targeting membrane receptors relies on the endogenous trafficking kinetics of specific RTKs, lysosome targeting receptors, or transmembrane E3 ligases involved, as well as their expression and colocalization (44, 45). Moreover, such antibodies require further modifications beyond the standard IgG format. Biparatopic antibodies, on the other hand, can be systematically designed against receptors such that the specific epitope combinations can promote receptor binding, trafficking, and degradation of target receptors (17, 35–37). If such antibodies can achieve comparable target degradation, they would be accompanied by the advantages of a standard IgG format, including long half life, high specificity, ability to recruit effector functions, and low immunogenicity (46). Thus, the rational engineering and screening of biparatopic antibody platforms may provide a simple yet powerful approach to target a broad range of receptor oncogenes.

Acquired secondary mutations in the FGFR2 kinase domain are an important mechanism of resistance to FGFR TKIs. Although next-generation covalent FGFR TKIs with broader spectrum activity against these mutations have been developed, on-target resistance remains a major limitation to monotherapy with these agents (3). We provide proof-of-concept data that biparatopic antibodies bpAb-B/C and bpAb-B/D targeting the FGFR2 ECD can overcome various kinase domain resistance in FGFR2 fusions. Indeed, previous studies have leveraged antibody or antibody combinations to overcome acquired resistance in other cancer settings, such as in the case of EGFR (47, 48). Thus, biparatopic antibodies with high activity and low toxicity have the therapeutic potential to target various forms of RTK resistance to small molecule kinase inhibitors.

We and others have shown that dual inhibition of oncogenes using 2 targeted agents having nonoverlapping patterns of cross resistance can delay or prevent the occurrence of on-target resistance (49, 50). Specifically, dual targeting of BCR-ABL oncogene with a combination of allosteric and catalytic ABL inhibitors acting at distinct sites are noncross resistant and eradicate CML tumors in preclinical models (50). Similarly, based on the observed synergy between bpAb-B/C and bpAb-B/D and FGFR inhibitors (Figure 7) we speculate that combination treatments of FGFR2 biparatopic antibodies and pan-FGFR inhibitors might delay or prevent the emergence of acquired resistance. A considerable advantage of highly active antibodies is the relative ease of combining such agents with small molecule inhibitors, as it has often been difficult to create well-tolerated combinations of targeted agents.

In all, our work has uncovered potent FGFR2 biparatopic antibodies as potential targeted treatment for FGFR2-driven ICC. Our results demonstrated that the engineering of biparatopic antibodies has the potential to lead to more effective and targeted treatments for a wide range of cancers.

## Methods

### Sex as a biological variable

Our study exclusively examined female mice because the female mice tend to engage in less aggressive behavior including fighting, compared with males. Similar phenotypes are reported in FGFR2-driven models in both sexes.

### Generation of DNA constructs and cell lines

*FGFR2-AHCYL1*(2), *FGFR2-BICC1*(2), and *FGFR2-PHGDH*(3) sequences were previously described as referenced. *FGFR2-AHCYL1* and *FGFR2-BICC1* constructs were synthesized (Genscript) and cloned into MSCV vector (addgene: #24828). *FGFR2* ECD with Ig subdomain deletions were generated based on *FGFR2-BICC* full-length sequence without AA37(Glu)-AA126(Asp) in Ig1 (D1), AA154(Pro)-AA247(Asp) in Ig2 (D23), AA250(Glu)-AA361(Gln), and AA154(Pro)-AA361(Gln) in Ig2-3 (D2 + D3) deletion constructs. All the mutant constructs were cloned into pBabe-puro-gateway via Gateway cloning strategy (addgene: #51070). All construct maps were sequence validated and aligned using SnapGene software.

To generate isogenic cell lines expressing FGFR2 fusions, retrovirus was generated by transfecting Platinum-E (Plat-E) retroviral packaging cell line (Cell Biolabs). For FGFR2 ECD WT and mutants, NIH3T3 (ATCC) and HEK-293T cells (ATCC) were transiently transfected with *FGFR2-BICC1* or its variants. Six parental antibodies and anti-human IgG1-FITC (Jackson Laboratories, #709-545-098) were used as primary and secondary antibodies, respectively, to validate the Ig-specific deletion mutants. Analysis was done using FlowJo v.10.8 software. ICC13-7 and CCLP-1 cholangiocarcinoma patient-derived cell lines were provided in-house and were authenticated via STR profiling.

### Biparatopic antibody design and generation

6 Parental antibody sequences were synthesized from the referenced sequences (Supplemental Table 1). To generate biparatopic antibodies, controlled Fab arm exchange reactions were performed where F405L and K409R-containing antibodies were mixed in an equimolar ratio according to the protocol (27). Immediately following the incubation period, the antibodies were buffer exchanged into PBS using a PD-10 desalting column (GE Healthcare) to remove the 2-MEA. To assess the quality and concentration of the bispecific antibodies, SDS-PAGE, SEC-HPLC, and mass spectrometry analysis were performed.

### Dimerization assay

For NanoBiT constructs, *FGFR2-WT*, *FGFR2-AHCYL1*, and *FGFR2-BICC1* were C-terminally tagged with Small BiT or Large BiT derived from NanoLuc (Promega). Full-length sequences were cloned into a pLenti and pLX304 retroviral vectors with puromycin and blasticidin selection markers, respectively. HEK293T cells were stably or transient transfected using TransIT-LT1 Transfection Reagent. Then, 24–30 hours after transfection, Nanoluc substrate (Nano-Glo Live Cell, Promega, N2011) was added the mixture was incubated at 37°C for 15 minutes, according to the manufacturer protocol. The luciferase activity was measured by EnVision plate reader (PerkinElmer).

### IHC

Tumors were surgically removed and placed in 10% neutral buffered formalin for 24 hours and followed by 70% ethanol until paraffin embedded. IHC was performed by Histowiz. Antibodies, anti-Ki67 (Abcam, ab15580), anti-IgG1 (Abcam, ab109489), and anti-mNKp46 (R&D, AF2225) were used at 1:100 dilution and hematoxylin solution was used for counterstaining.

### Immunoblotting

Cell lysates in RIPA buffer (50 mM Tris pH 7.4, 150 mM NaCl, 1% NP-40, 0.5% sodium deoxycholate and 0.1% SDS) were resolved on 8% or 4%–20% Tris-Glycine gels and transferred to PVDF membranes (Novex). The following antibodies were used as primary antibodies at 1:1,000 dilution and were obtained from Cell Signaling Technologies:

AKT (Catalog 2920), pAKT (S473) (Catalog 4060), ERK1/2 (Catalog 4695), pERK1/2 (T202/Y204) (Catalog 9106), pFGFR (Y653/654) (Catalog 3471), pFRS2(Y436) (Catalog 3861), pFRS2(Y196) (Catalog 3864), GAPDH (Catalog 97166), MEK1/2 (Catalog 4694), pMEK1/2 (S217/221) (Catalog 9154), and Tubulin (Catalog 3873); and from Genscript: FGFR2 (parental antibody E); Abcam: FRS2(Catalog ab183492); and Sigma-Aldrich: Vinculin (Catalog V9131).

### Transformation assays

#### Focus formation assay.

NIH3T3 stably expressing FGFR2 fusions were plated at 5 × 10^5^cells per well in 6-well plate in triplicate. Cells were grown for 7–10 days, plates were imaged, and the number of foci were blindly counted.

#### Soft agar colony formation assays.

NIH3T3 cells stably expressing patient-derived oncogenic FGFR2 variants were plated at 1 × 10^4^ cells per well in 6-well plates with 0.5% Select Agar (Thermo Fisher Catalog 30391049). Cells were cultured for 2–3 weeks, colonies were imaged, and colony numbers were determined using ImageJ and Prism software.

#### BaF3 transformation assay.

BaF3 cells (Creative Bioarray) were resuspended in RPMI media + 10% FBS with 0% IL-3. Cells were seeded at 20,000 cells per well in 6-well plate and were split every 3 days. For each split, Cell-titer Glo was used to measure the cell viability compared with original seeding density and the new seeding density was determined. Cumulative population doublings were calculated at each split from log_2_(current density/previous density/split) over the period of 15–20 days. All antibodies were added to a final concentration of 2 μM and were replaced every 3 days during each passage.

### Binding affinity and epitope binning assays

#### Meso Scale Discovery-Solution Equilibrium Titration.

Measurements were performed according to the previously published protocol (51). Briefly, in a 96 well assay plate, a constant concentration of antibody is incubated with titrating concentrations of antigen in an assay buffer PBS 1x pH7.4, 0.1% BSA (Sigma-Aldrich) w/v, 0.02% P20 (Thermo Fisher). Once the antibody-antigen interaction is reached, the free antibody is transferred and quantified by allowing it to incubate on an antigen-coated meso scale discovery (MSD) plate (PN: L15XA-3). Then, subsequent detection with an ECL-labeled secondary antibody was performed. Experiments were performed as independent duplicates.

#### BLI-Octet.

Binding kinetics (ka, kd) and affinity (kd) were measured in an Octet system RED96e at 25°C with shaking at 1,000 rpm using 1× kinetic buffer (Sartorius, PN: 18-1105). Antibodies were captured by Anti-Human Fc capture biosensor (AHC) (Sartorius, PN: 18-5060) for 300 seconds at 0.5 μg/mL. hFGFR2 ECD 22-378 His-tag (SinoBiological; PN: 16485-H08H) was used as an analyte, with 7 2-fold dilutions from 100 nM using DFx2. Association and dissociation of the analyte to the captured antibody was monitored for 300 seconds and 600 seconds, respectively. Data were analyzed using the Octet Data Analysis software HT 12.0. Sensorgrams were fitted to a 1:1 binding model where kinetic rate ka and kd were globally fitted.

#### Epitope binning.

Epitope binning experiments were performed in an Octet system RED96e at 25°C with shaking at 1,000 rpm using 1× kinetic buffer (Sartorius, PN: 18-1105). To perform an in-tandem epitope binning experiment, biotinylated hFGFR2 ECD AA22-AA378 His-tag (SinoBiological, PN: 16485-H08H) was captured on streptavidin sensor (SA) (Sartorius, PN: 18-5020) for 300s at 1 μg/mL concentration. hFGFR2 was biotinylated using Abcam Biotinylation Kit (PN: ab201796). The cycle starts with the capturing of biotinylated ligand followed by a “primary” antibody (Ab1) binding step where Ab1 interaction is monitored for 600 seconds at 333 nM concentration. Shortly after, a “competing” antibody (Ab2) interaction was monitored for 300 seconds at 333 nM concentration. All antibodies are used at a concentration greater than 10 × Kd to ensure ligand saturation. Data were blindly analyzed using the Octet Data Analysis software HT 12.0 and R Studio “pvclust” according to Octet Application note n.16.

### Avidity measurement

NIH3T3 cells expressing FGFR2-PHGDH were resuspended at a concentration of 8.0 × 10^7^ cells/mL and seeded on z-Movi (LUMICKS Inc) microfluidic chips that were coated with Poly L-Lysine (Sigma-Aldrich, P4707). Z-Movi chips seeded with 3T3 cells were placed in a 37°C dry incubator for at least 2 hours for attachment. 20 μL of antibody-on-beads were flowed onto the z-Movi chip and incubated with the target 3T3 cells for 30 seconds. Following incubation, an acoustic force ramp from 0–1,000 pN over 2:30 minutes was applied within the z-Movi chip and antibody-on-bead detachment was observed using real-time fluorescence imaging on the z-Movi system. Each z-Movi chip was used to sequentially flow in negative control, parental antibody pair, and corresponding biparatopic antibody–coated beads. Replicates were performed on different z-Movi chips with randomized run orders for antibody conditions. Avidity experiments were processed using proprietary Oceon software.

### Flow cytometry

#### Apparent affinity analysis.

1 × 10^6^ of NIH3T3 cells expressing full-length and FGFR2-BICC1 variants (D1, D2, D3, or D2+D3 deletion variants), SNU-16 cells, or parental BaF3 cells (negative control) per tube were incubated with parental antibody A-F at final concentration of 10 μg/mL (NIH3T3) or at serial dilutions of 0, 1, 10, 50, and 100 ng/mL, and 1 and 10 mg/mL (SNU-16) in 1 × PBS (Mg^2+^ free) for 1.5 hours at room temperature (52). Cells were washed 3 times with FACS buffer (1 × PBS, 1% BSA, 5% FBS) and incubated with goat anti-human IgG Alexa Fluor 488 (Jackson ImmunoResearch, Catalog 109-545-098) secondary antibody for 30 minutes, washed, and analyzed on a SA3800 Spectral Analyzer (Sony Biotechnology). Data were analyzed using FlowJo v.10 software and fit in GraphPad Prism 9 using a ligand-binding quadratic equation to obtain KD values.

#### Antibody internalization assay.

7.5 × 10^5^ of BaF3 cells expressing FGFR2-PHGDH were distributed in each tube for each condition. All antibodies were added to wells at a final concentration of 5 μg/mL in serum-free RPMI media and incubated for 1 hour on ice. After washing to remove excess antibodies, cells were transferred to 4°C or 37°C for 1, 2, 3, 4, and 16 hours, then washed 3 times with FACS buffer. Surface FGFR2-bound parental or biparatopic antibodies were detected with goat anti-human IgG Alexa Fluor 488 secondary antibody and analyzed on a CytoFLEX S (Beckman Counter). The geometric mean of signal per sample was determined using FlowJo v.10 software.

#### Fabfluor receptor degradation.

NIH3T3 or ICC13-7 cells were seeded at 7,500 cells per well in 96 well-plate (Corning, Catalog 3595). Red Incucyte Fabfluor-pH Antibody Label reagents (Sartorius, Catalog 4722) (33) with stock concentrations at 0.5 mg/mL were mixed and incubated with each antibody at 1:3 molar ratio of antibody:Fabfluor label for 30 minutes at 37°C. Antibody-Fabfluor label mix were added to the cells at 4 μg/mL final concentration. Images were taken by Incucyte at original magnification ×20 every 30 minutes for up to 72 hours. Analysis was done using Incucyte Basic Analyzer with Top-Hat background subtraction. Red Total Integrated Intensity Per Well (RCU/OCU × μm^2^/well) was quantified as a readout using Incucyte software v2019B.

### Growth inhibition assay

Engineered BaF3 cells expressing FGFR2-PHGDH and FGFR2-AHCYL1 cells were seeded at 7,500 cells/well in 0% IL3, RPMI + 10% FBS media in 96 well-plates (Corning, Catalog 3904). Parental antibodies, biparatopic antibodies, or IgG1 control (Bio X Cell, Catalog BP0297) were added 24 hours after seeding at 15 serial concentrations ranging from 0 to 1 μM. For viability assay in the presence of FGF10, FGF10 (R&D Systems, Catalog 345-FG-025) were added 4 hours after the antibody treatment at a final concentration of 100 ng/mL. Viability was determined using CellTiter-Glo 2.0 (Promega) at day 5 after treatment, according to the manufacturer’s instructions.

### ADCC and ADCC activity assays

For *NK cell killing assay,* ICC13-7 were seeded into 96-well black-clear bottom plates (Corning) at 5,000 cells per well. IncuCyte CytoLight Rapid Green Reagent (Essen BioScience, Cat 4705) was added to each well at a concentration of 330 nM for cytoplasmic labeling, and were cells incubated overnight. Engineered NK-92 cells (53) were added to each well in 50 μL of MyeloCult H5100 medium (STEMCELL) with 12.5% heat-inactivated horse serum (Gibco) and 100 units/mL human recombinant IL-2 (PeproTech, Cat AF-200-02). FGFR2 biparatopic antibodies or an IgG control were added in 50 μL of the same medium, containing IncuCyte Annexin V Red (Essen BioScience, Cat 4641, 1:500) and was imaged using IncuCyte S3 (Essen BioScience). For ADCC and ADCP reporter assays, ICC13-7 were seeded at 5,000 cells per well with Jurkat-NFAT-hCD16 (ADCC) and Jurkat-NFAT-hCD32 cells (ADCP) (InvivoGen, Cat jktl-nfat-cd16, jktl-nfat-cd32) at 20,000 cells for 24 hours, QUAnti-Luc 4 Reagent was added, and the plate were analyzed in EnVision.

### Mouse xenograft experiments

A total of 5 × 10^6^ of BaF3 cells expressing FGFR2-PHGDH or 3 × 10^6^ of ICC13-7 cells in a total volume of 200 μL (100 μL Matrigel + 100 μL PBS) were subcutaneous implanted in the right flank of 7–9 week-old female BALB/c scid mice (Jackson Laboratory, strain 001803). At a tumor size of approximately 250 mm^3^ (BaF3) or approximately 150 mm^3^ (ICC13-7), mice were randomized into 10 groups, 10 mice per treatment group. Biparatopic antibodies, parental antibodies, or IgG1 (Bio X Cell, Catalog BP0297) were IV administered twice per week and tumor sizes were measured by caliper every 3–4 days for 25 days (BaF3) and 38 days (ICC13-7). Tumor volume was calculated by the modified ellipsoidal formula: V = 0.523 × (L × W^2^) where L = the greatest longitudinal diameter and W = the greatest transverse diameter (width). 1-way ANOVA multiple comparisons (Friedman’s ANOVA multiple comparisons) statistical analysis was used to compare tumor sizes among all paired groups.

### RNA sequencing analysis

Tumors were surgically removed, flash frozen in liquid nitrogen, and processed for RNA sequencing (Azenta). A combined human-mouse genome reference was constructed and RNA-seq reads from samples were aligned to this integrated genome using STAR aligner (54). Feature Counts was used to quantify reads mapped specifically to mouse-derived genes, providing gene-level counts. For differential expression analysis (DEG), edgeR package was used (55). After obtaining raw *P* values for each gene, we applied FDR correction to control for multiple testing, resulting in a list of significant DEGs with adjusted *P* values. To estimate overall ADCC, ADCP, and CDC pathway activity, we selected 5 GO terms: 0002228, 0001788, 0002431, 0002281, 0002430. The overall activity score was calculated by taking a weighted sum of the gene expression values within each GO term (assigning equal weights of 1 to each gene) and dividing by the total sum of weights. IgG1 group was used as a reference and *t* tests were conducted to determine whether any GO term activity score in different treatment groups differed significantly from this control group. We applied FDR correction to *P* value to adjust for multiple comparisons, resulting in adjusted *P* values.

### ELISA assay

Blood samples were collected from the submandibular veins of mice at 1, 24, and 72 hours after the last dose of the treatment before the harvest. Levels of plasma antibody were measured with the Human IgG Total ELISA Kit (Sigma-Aldrich) per manufacturer’s instructions. The absorbance was measured with EnVision (PerkinElmer).

### Phospho-receptor tyrosine kinase profiling

Protein was prepared per protocol (Human Phospho-RTK Array Kit, Catalog ARY001B): Cells were starved of FBS and treated with antibodies (1 μM) for 5 hours. Cells were harvested in lysis buffer provided in kit with protease and phosphatase inhibitors added before use. Membranes were exposed to X-ray film (Fuji) for multiple exposure times and dots were mapped using reference spots provided and analyzed for relative intensity using ImageJ.

### Statistics

All statistical analyses were performed using GraphPad Prism 9.0 or 10.0. Data are reported as mean ± SEM. 1-way ANOVA multiple comparisons was used to calculate *P* values for comparisons of 3 or more groups. Friedman’s ANOVA multiple comparisons were used to compare between treatment groups in xenograft experiments. Samples analyzed from in vivo experiments were randomly selected with no exclusion criteria. *P* values of less than 0.05 were considered significant. Statistical parameters can be found in the figure legends.

### Study approval

All in vivo experiments were conducted under protocol 0121-09-16-1 approved by the Broad Institute’s Institutional Animal Care and Use Committee (IACUC).

### Data availability

RNA sequencing data was deposited with GEO accession number: GSE281992. The unedited blots are provided in the supplemental material. Values used for graphs in figures and reported means are provided in the Supporting Data Values file.

## Author contributions

SC and WRS conceived, designed, and analyzed the experiments and wrote the manuscript. SC, SO, DTF, JJK, FPR, TYS, YCC, and MC performed antibody validations, in vitro activity assays, and mechanistic validation experiments. AS and SC characterized antibody binding epitopes and affinities. JJK, SC, DJR, and LC performed in vivo xenografts experiments. DK provided antibody-antigen structural insights. DTF, JJK, AA, RD, YYT, and YH processed and analyzed xenograft-derived samples. NB provided ICC models and critical insights into ICC biology and FGFR inhibitors. All authors reviewed and edited the manuscript.

## Supplementary Material

Supplemental data

Unedited blot and gel images

Supporting data values

## Version 1 02/27/2025

In-Press Preview

## Version 2 04/15/2025

Electronic publication
