# Supplementary material for: Identification of potent biparatopic antibodies targeting FGFR2 fusion–driven cholangiocarcinoma
Source: J Clin Invest. 2025 Feb 27;135(8):e182417. doi: 10.1172/JCI182417 (PMC11996885; doi:10.1172/JCI182417)
Supplement: Supplemental data [file jci-135-182417-s162.pdf]

Supplemental Figure. 1

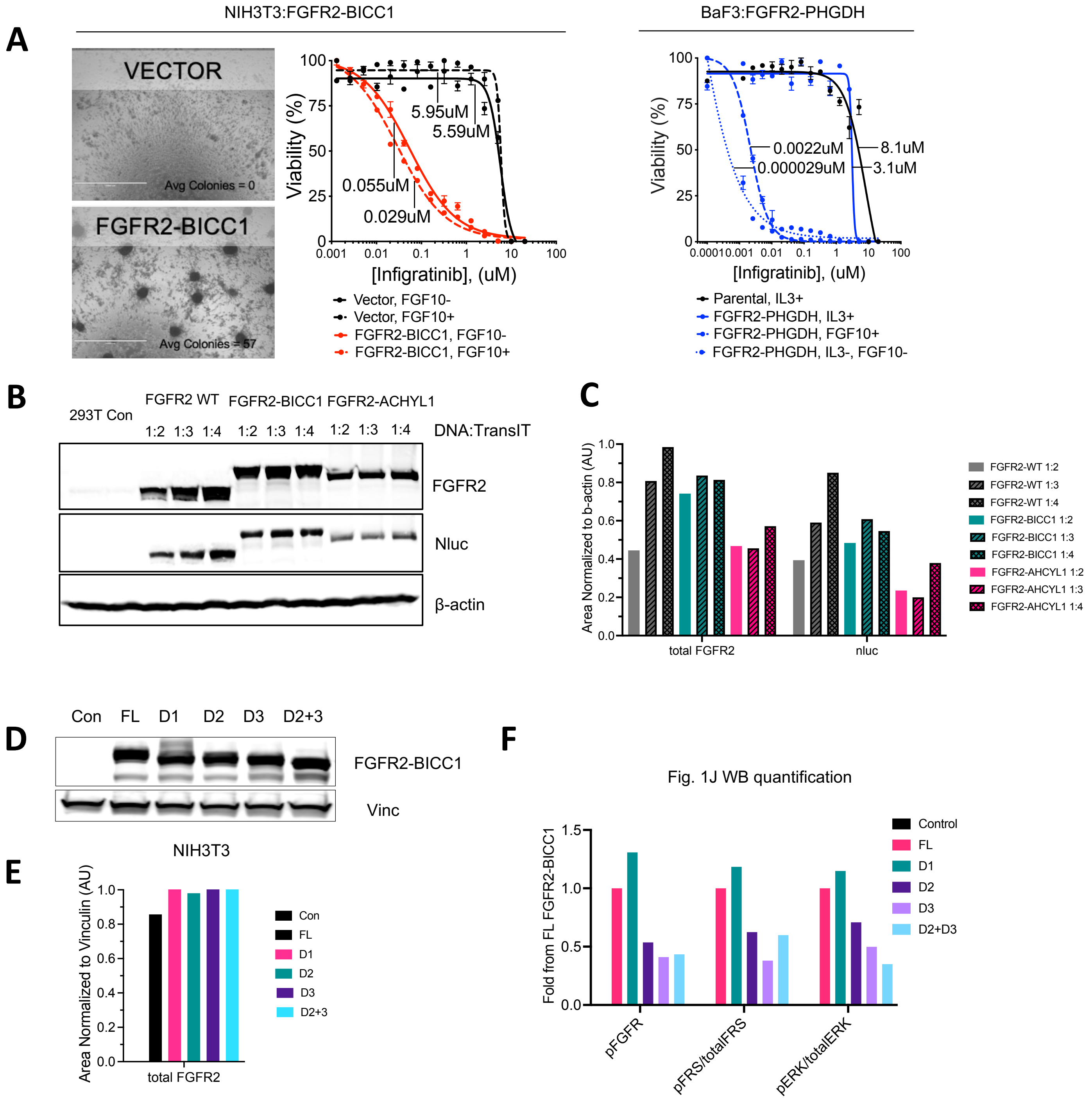

Supplemental Figure 1: The extracellular domain is necessary for full transformation by FGFR2-fusions

(**A**) Focus formation assay of FGFR2-BICC1 expressing NIH3T3 cells and its sensitivity to infigratinib; IC50s are indicated (left). Transformation assay of FGFR2-PHGDH expressing BaF3 cells and their sensitivity to infigratinib with indicated IC50s (right).

(**B-C**) Immunoblot analysis of FGFR2-WT, FGFR2-ACHYL1, and FGFR2-BICC1 with small and Large BiT expression levels in HEK293 cells using various viral titers (**B**) and their quantifications (**C**).

(**D-E**) Immunoblot image of full-length as well as D1, D2, D3, and D2+3 deleted FGFR2-BICC1 overexpressing NIH3T3 (**D**) and its quantification (**E**).

(**F**) Quantification of Fig. 1J immunoblot showing downstream FGFR2 signaling in con, FL or in D1, D2, D3, or D2+D3 domain-deleted ECD.

Supplemental Figure. 2

A

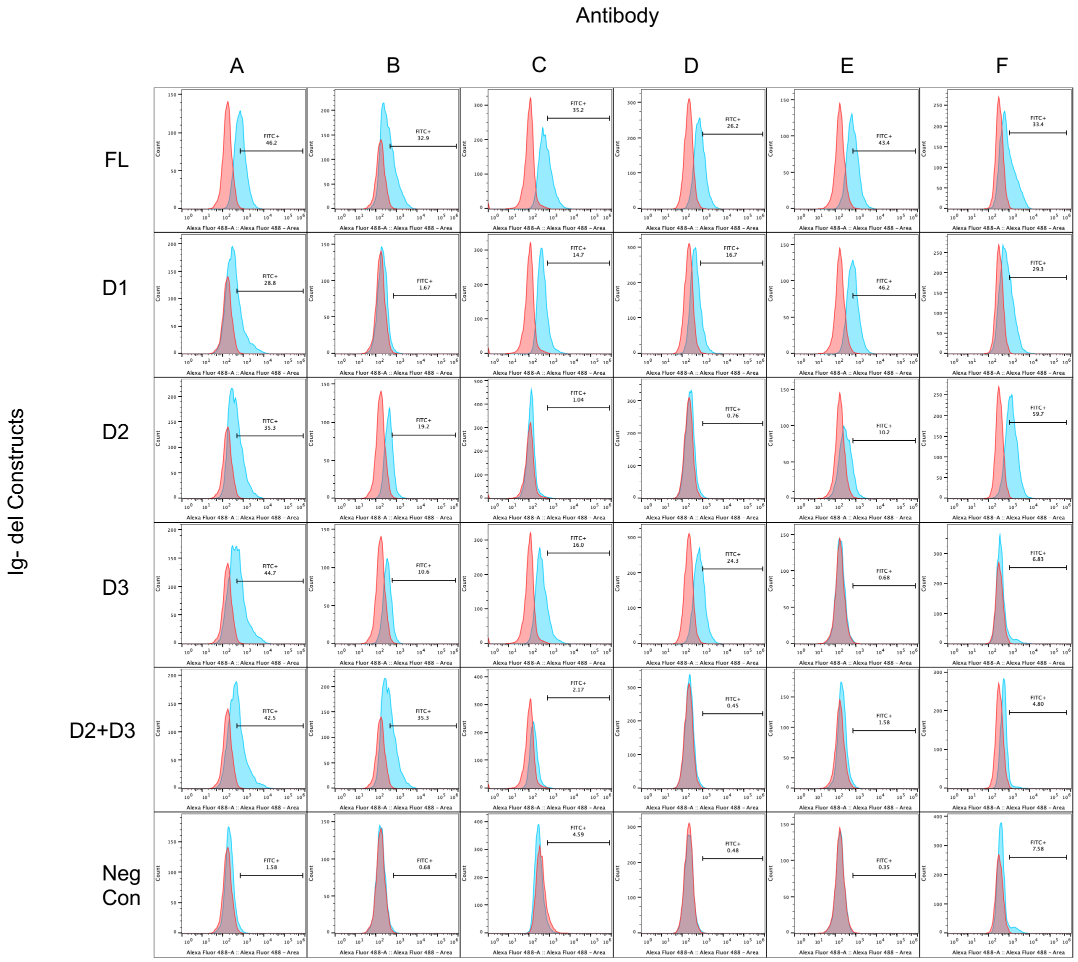

% FITC pos (norm to max binding group)

Antibody/ Deletion construct	A	B	C	D	E	F
FL	100	93.2011331	100	100	93.9393939	55.9463987
D1	62.3376623	4.73087819	41.7613636	63.740458	100	49.078727
D2	76.4069264	54.3909348	2.95454545	2.90076336	22.0779221	100
D2+3	91.991342	100	6.16477273	1.71755725	3.41991342	8.04020101
D3	96.7532468	30.0283286	45.4545455	92.7480916	1.47186147	10.6867672
Con	3.41991342	1.92634561	4.51704545	1.83206107	0.75757576	4.32160804

B

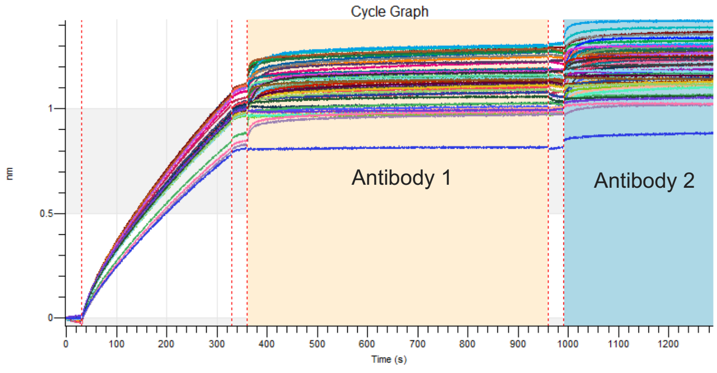

		Antibody2 (competing antibody)					
	Ab#	A	B	D	C	E	F
Antibody 1 (saturating antibody)	A	0.0373	0.1781	0.1294	0.1293	0.1024	0.1059
	B	0.0398	0.0159	0.1145	0.1163	0.0830	0.0884
	D	0.0514	0.1640	0.0107	0.0258	0.0519	0.0826
	C	0.0340	0.1249	0.0031	0.0321	0.0648	0.0672
	E	0.0508	0.1651	0.0965	0.1139	0.0396	0.0435
	F	0.0480	0.1669	0.1085	0.1215	0.0204	0.0252

Epitope binning competition matrix
(BLI data Input for RStudio pvclust)

C

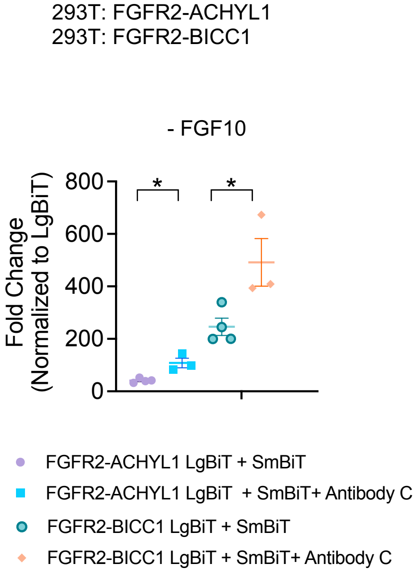

Supplemental Figure 2: Development of candidate biparatopic antibodies directed against FGFR2

(A) Flow cytometry histogram analysis of NIH3T3 cells overexpressing FL, D1, D2, D3, or D2+D3 deleted FGFR2 upon treatment with IgG1 control (red) or parental antibodies A-F (blue). Anti-human Fc Alexa-488 was used as a secondary antibody. Table demonstrates binding (% of FITC+ cells) compared to max binding (100%) of that particular antibody to FGFR2 expressing NIH3T3 cells.

(B) Sensorgram images of data generated by a Bio-Layer Interferometry (BLI) epitope binning experiment. Antibody 1 are saturating antibodies that were added first and antibody 2 are competing antibodies that were subsequently added. The table shows raw data matrix used to generate binning in figure 2C on RStudio.

(C) NanoBiT receptor dimerization assay of FGFR2-BICC1 and FGFR2-ACHYL1 overexpressing NIH3T3 cells with antibodies C at 3uM in the absence of FGF ligand. All data are mean ± SEM. ns=not significant, *$P < 0.05$, **$P < 0.01$, ***$P < 0.001$, ****$P < 0.0001$ by One-way ANOVA multiple comparisons.

Supplemental Figure. 3

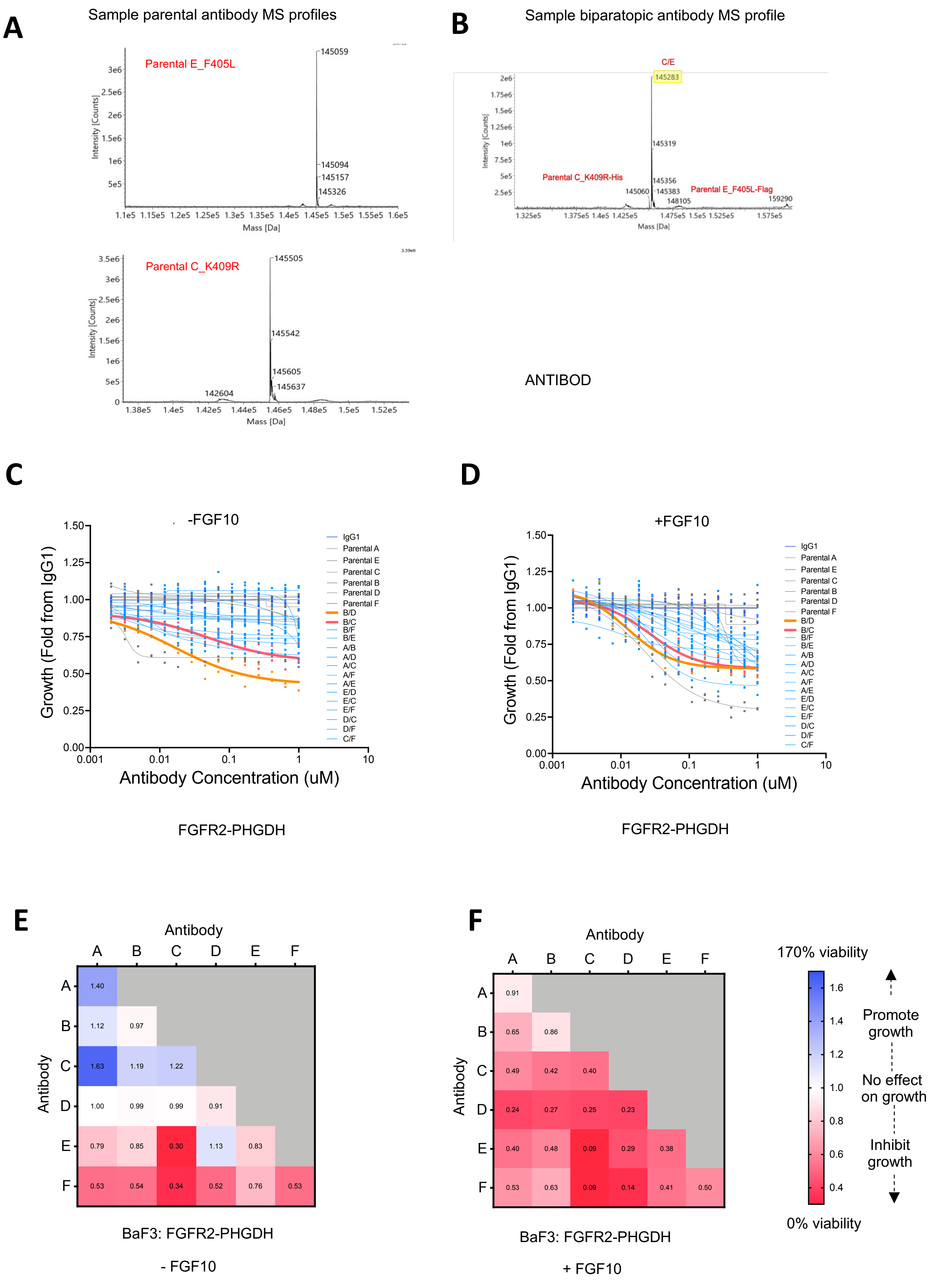

G

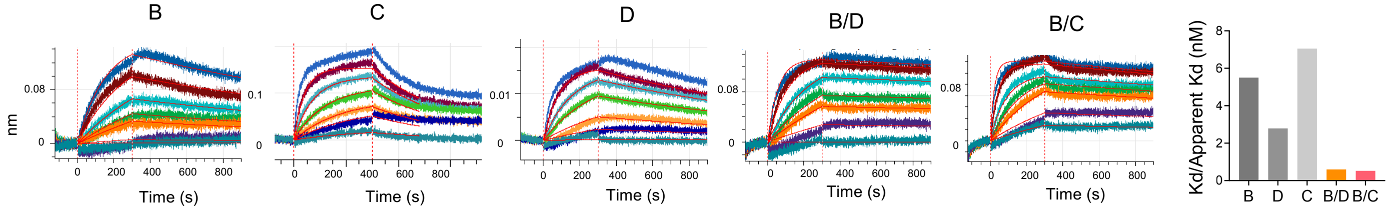

H

Antibody	Kd (nM)	SD	ka (1/Ms)	SD	koff (1/s)	SD	Note
B	5.5	3.46	5.31E+04	1.41E+04	3.16E-04	2.61E-04	n=2
C	7.05	0.68	4.17E+05	6.46E+04	2.92E-03	6.08E-05	n=3
D	2.79	0.27	2.82E+05	4.33E+04	7.41E-04	9.35E-05	n=3
B/D	0.6	n/a	1.77E+05	n/a	1.06E-04	n/a	n=1
B/C	0.52	n/a	4.51E+05	n/a	2.35E-04	n/a	n=1

Supplemental Figure 3: Identification of potent tumor growth-inhibiting biparatopic antibodies via unbiased screening

(**A**) Representative mass spectrometry data of the parental antibodies E (top) and C (bottom) bearing the indicated complementary mutations.

(**B**) Representative mass spectrometry data of products from the least efficient reaction with low yield (C/E duobody product at ~145282 peak) and high parental antibody contaminants (Parental C at ~145060 peak and Parental E at ~145505 peak (top). Mass spectrometry data showing products from the most efficient reaction with highest yield (C/E duobody product at ~145282 peak) and minimal parental antibody contaminants (Parental C at ~145060 peak and Parental E at ~145505 peak (bottom).

(**C-D**) Viability of FGFR2-PHGDH overexpressing BaF3 cells upon treatment with IgG1 control, biparatopic antibodies, and their parental antibodies at increasing concentrations in the absence (**C**) and presence of FGF10 (**D**).

(**E-F**) Viability of FGFR2-PHDGH expressing BaF3 cells upon treatment with parental antibody mixtures each at a concentration of 3uM in the absence (**E**) or presence of FGF10 (**F**) (average fold from IgG1 treated controls). Values < 1 indicate growth inhibition, values ~ 1 indicate no significant impact on growth, and values >1 indicate growth promoting effects upon treatment with indicated parental antibody mixtures.

(**G**) Representative binding sensorgrams illustrating the binding kinetics between FGFR2 ECD and antibody B, D, C or biparatopic antibody bpAb-B/C and bpAb-B/D via BLI-Octet. Kd values were determined based on the association and dissociation curves.

Supplemental Figure. 4

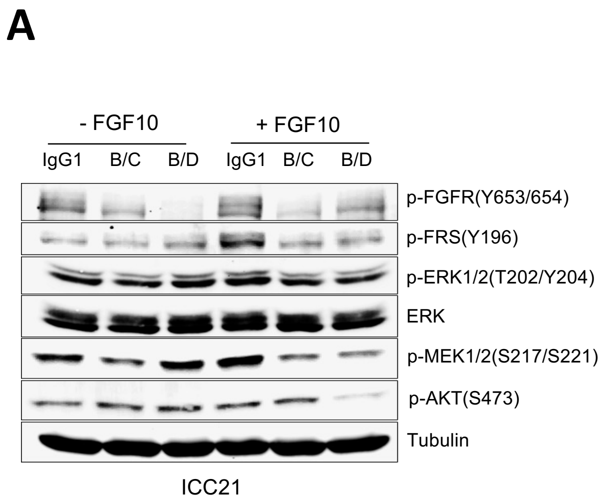

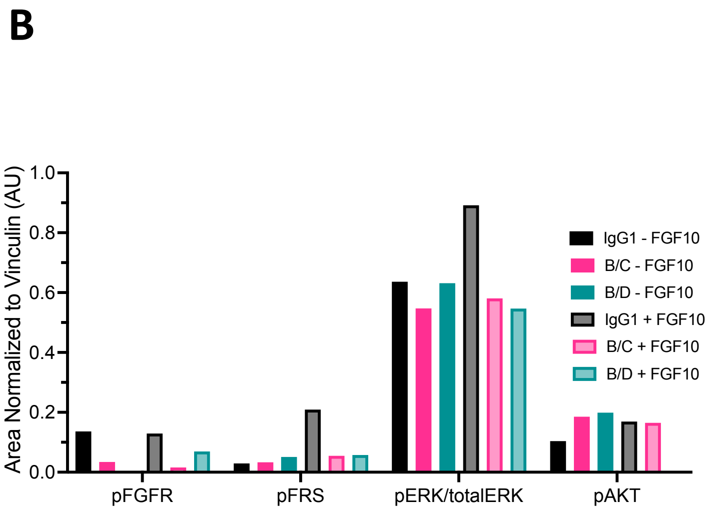

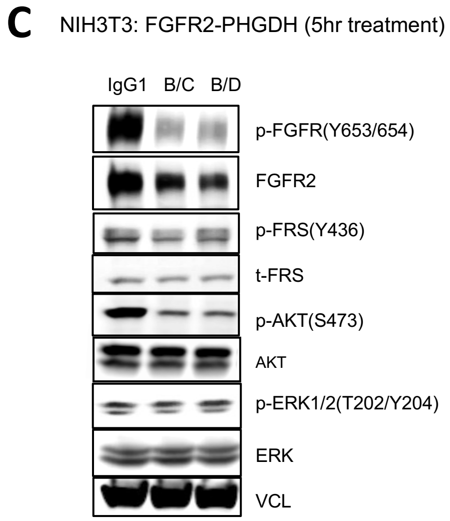

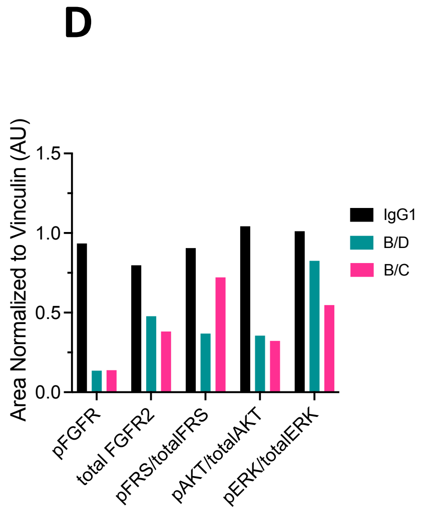

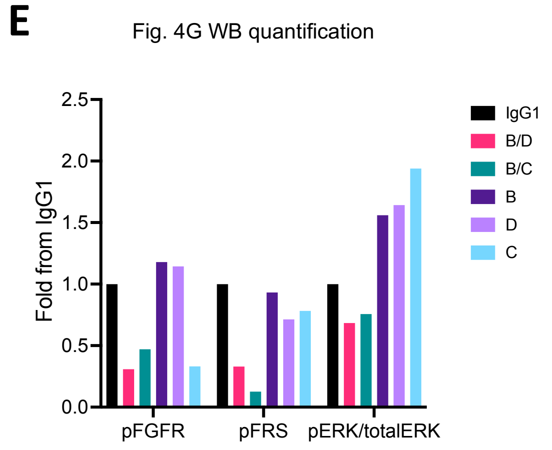

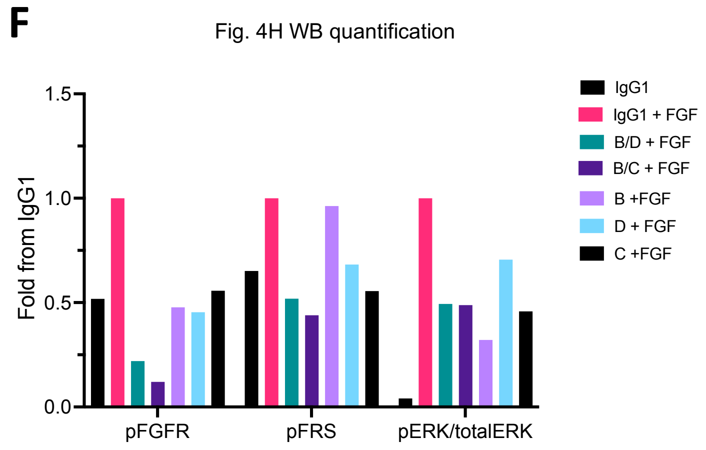

Supplemental Figure 4: Biparatopic antibodies show superior inhibition of growth and transformation of a FGFR2 fusion-driven cholangiocarcinoma cell line.

(**A-B**) Immunoblot of ICC21 upon 5hrs after treatments with bpAb-B/C, or bpAb-B/D compared to the parental antibodies B, D, C in the absence or presence of FGF10 ligand (**A**) and its quantification (**B**).

(**C-D**) Immunoblot analysis of FGFR2-PGHDH expressing NIH3T3 cells upon treatment with bpAb-B/C, bpAb-B/D, or IgG1 control (**C**) and its quantification (**D**).

(**E-F**) Quantification of immunoblot in Fig. 4G (**E**) and Fig. 4H (**F**), showing ICC13-7 cells upon 5 h treatments with bpAb-B/C, or bpAb-B/D compared to the parental antibodies B, D, C in the absence (**E**) or presence (**F**) of FGF10 ligand

Supplemental Figure. 5

A

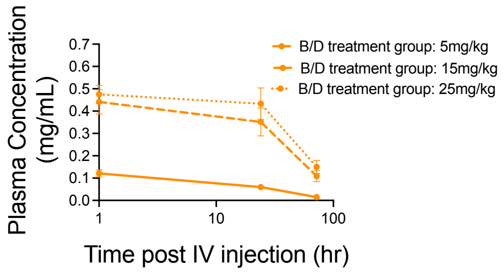

B

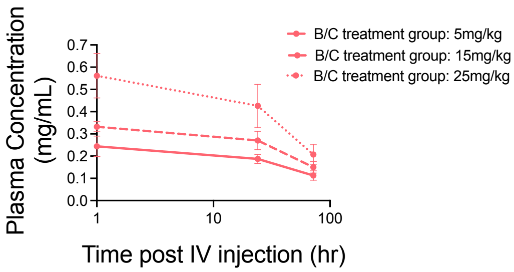

	5mg/kg	15mg/kg	25mg/kg
Half-life (h)	23.2	50.1	55.8

	5mg/kg	15mg/kg	25mg/kg
Half-life (h)	56.6	65.2	55.6

C

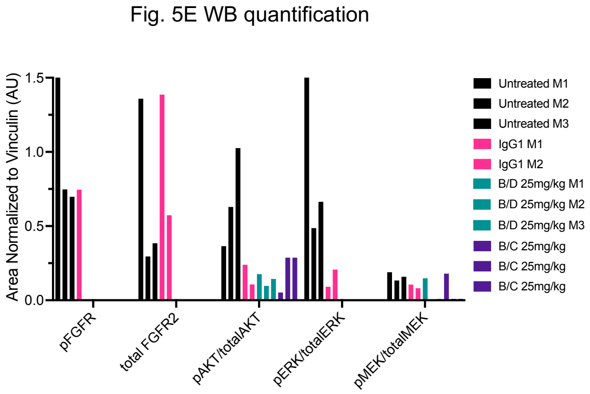

D

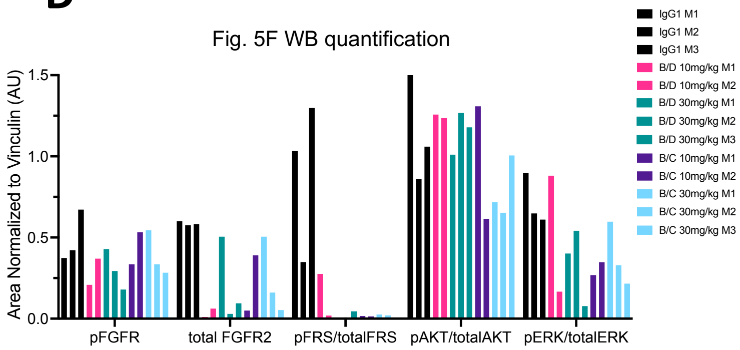

E

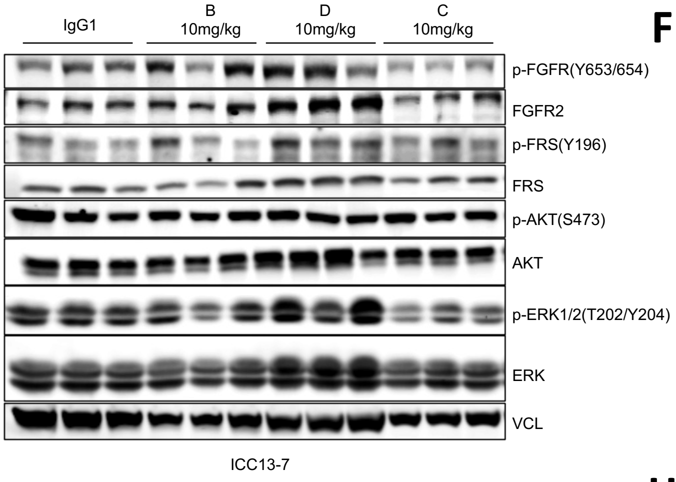

F

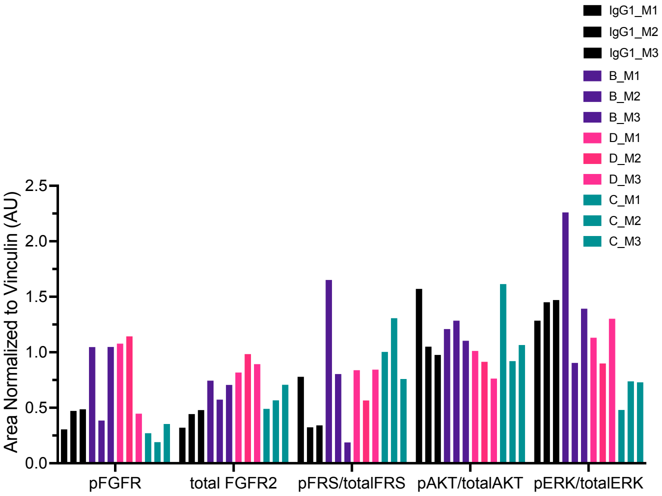

G

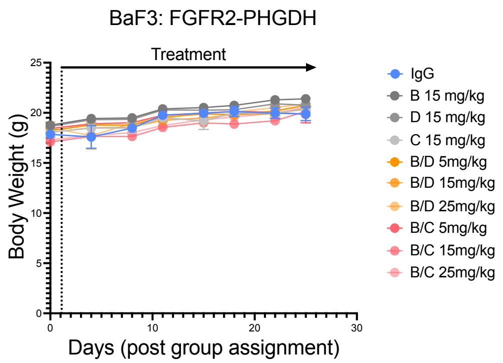

H

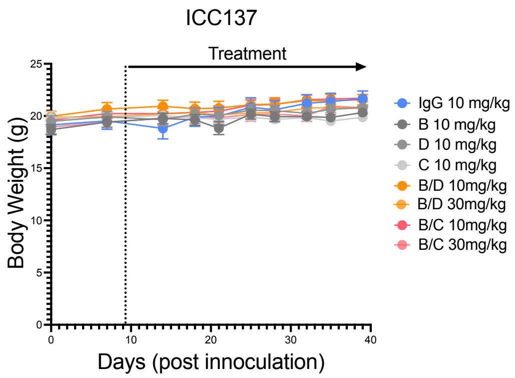

I

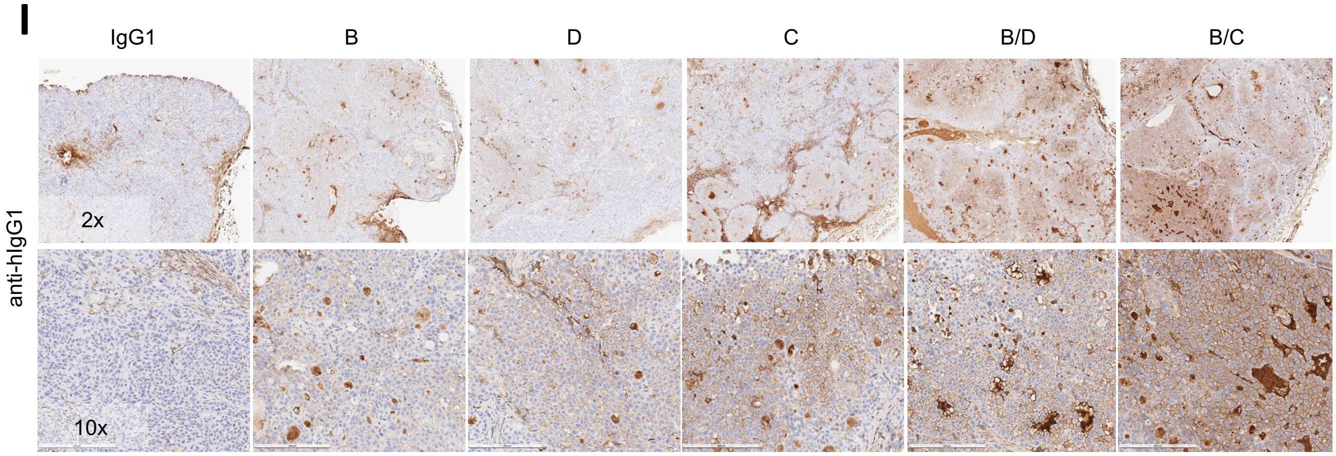
IgG1
B
D
C
B/D
B/C
anti-hIgG1
2x
10x

J

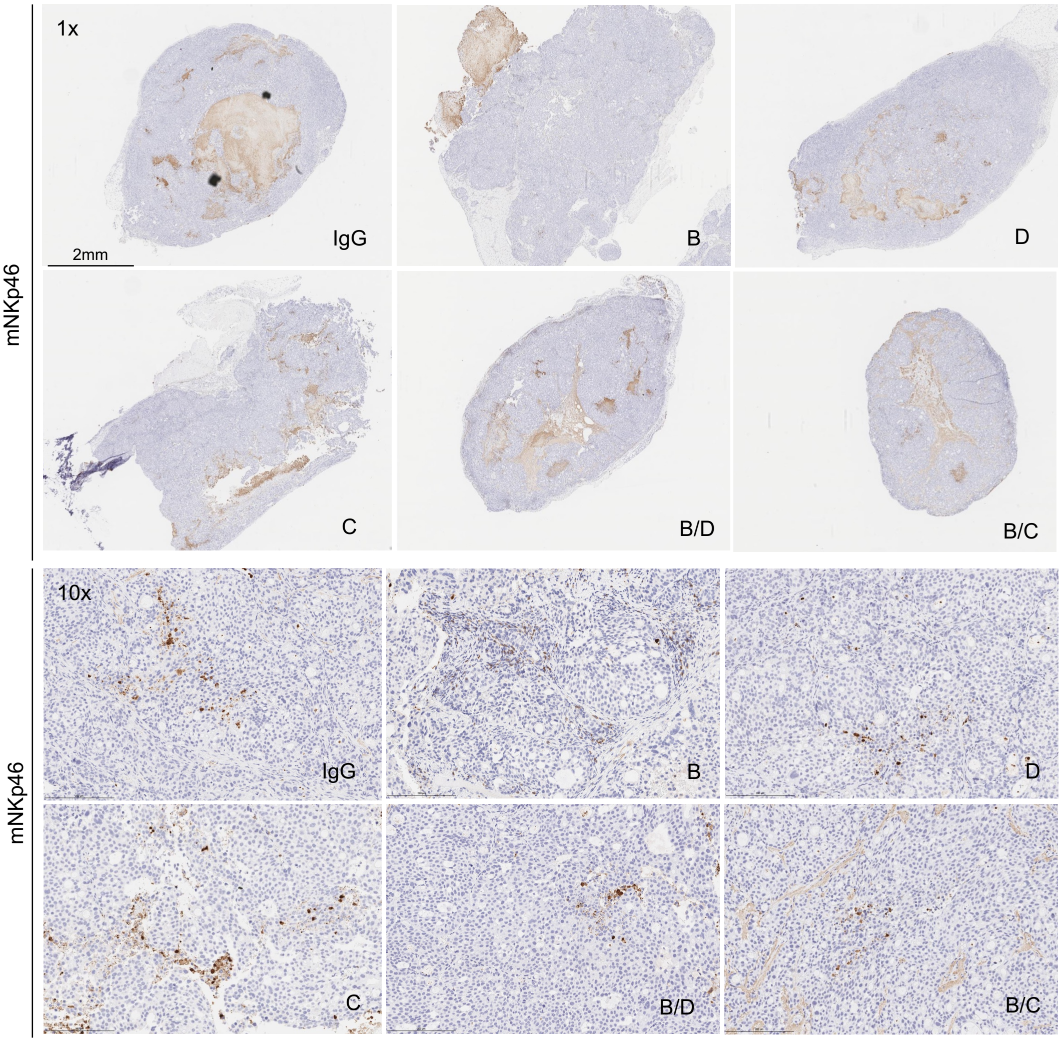
mNKp46
1x
2mm
IgG
B
D
C
B/D
B/C
mNKp46
10x
IgG
B
D
C
B/D
B/C

K

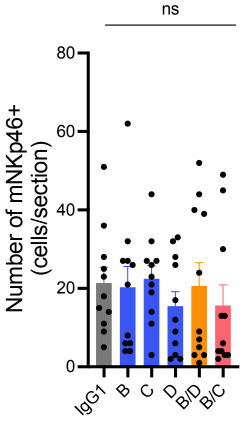
ns
Number of mNKp46+
(cells/section)
80
60
40
20
0
IgG1
B
C
D
B/D
B/C

L

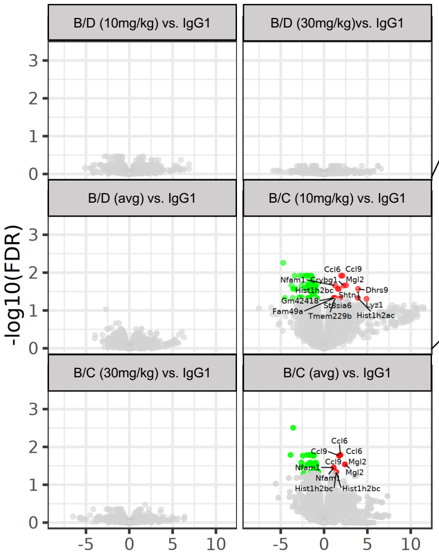

Gene		Gene Ontology: Biogical Process
CCL6	C-C motif chemokine ligand 6(Ccl6)	Chemokine mediated - signaling pahtway
CCL9	C-C motif chemokine ligand 9(Ccl9)	Chemokine mediated - signaling pahtway
NFAM1	Nfat activating molecule with ITAM motif 1(Nfam1)	Immune response regulating cell surface receptor signaling pathway
MGL2	macrophage galactose N-acetyl-galactosamine specific lectin 2(Mgl2)	Adaptive immune response
DHRS9	dehydrogenase/reductase 9(Dhrs9)	
CRYBG1	crystallin beta-gamma domain containing 1(Crybg1)	
LYZ1	lysozyme 1(Lyz1)	
ST8SIA6	ST8 alpha-N-acetyl-neuraminide alpha-2,8-sialyltransferase 6(St8sia6)	
TMEM229B	transmembrane protein 229B(Tmem229b)	

M

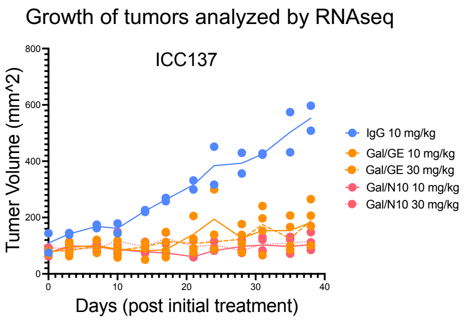

N

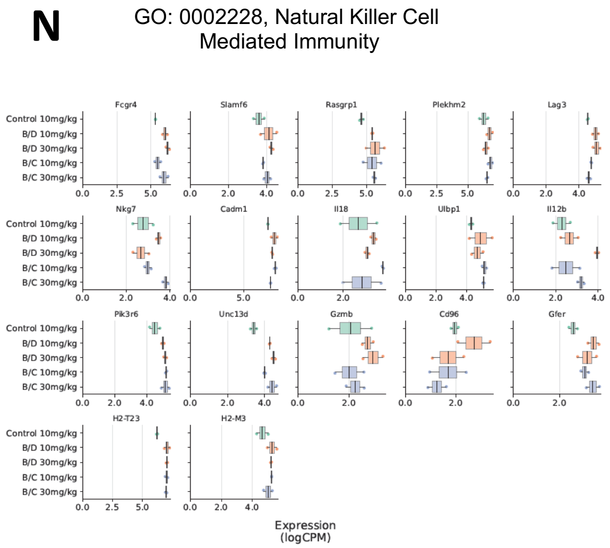

O

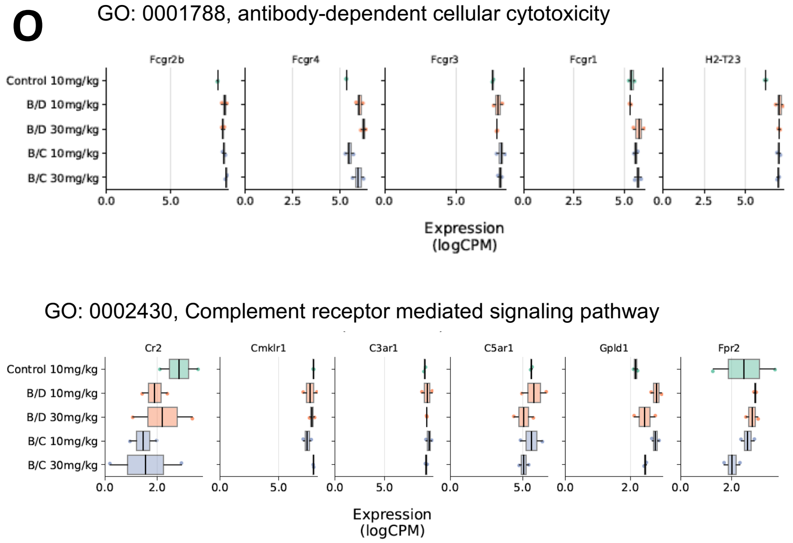

P

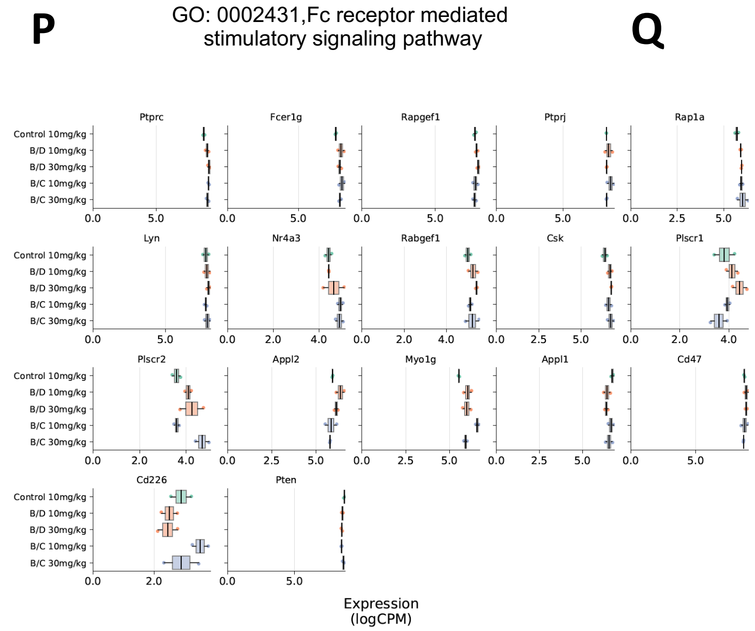

Q

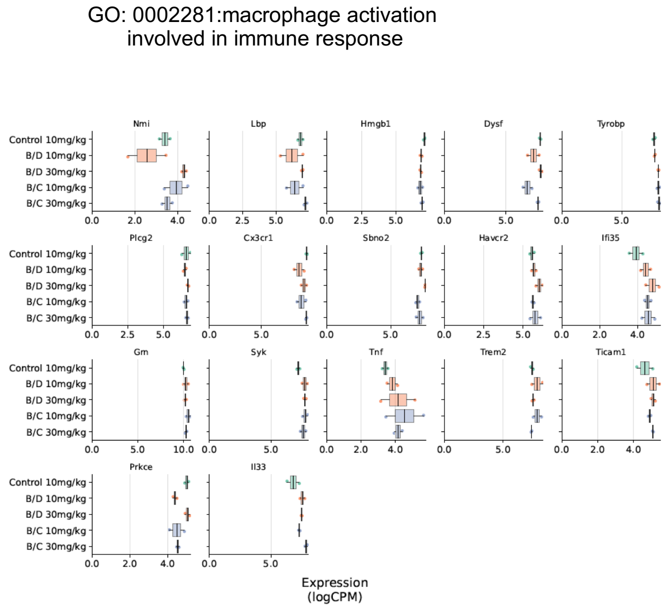

R

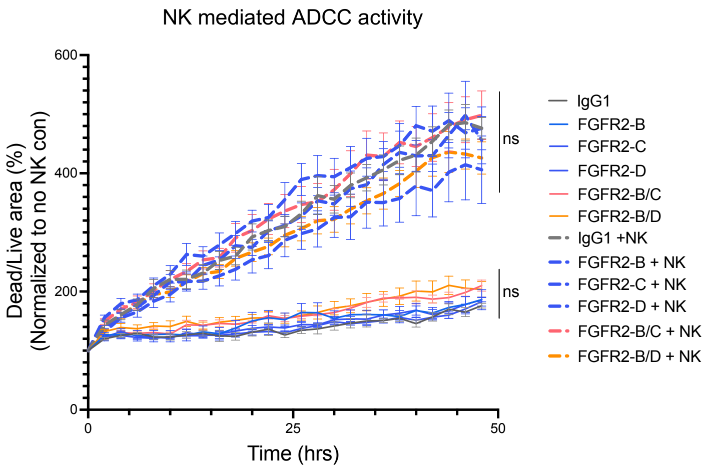
NK mediated ADCC activity
Dead/Live area (%)
(Normalized to no NK con)
600
400
200
0
0
25
50
Time (hrs)
ns
ns
IgG1
FGFR2-B
FGFR2-C
FGFR2-D
FGFR2-B/C
FGFR2-B/D
IgG1 +NK
FGFR2-B + NK
FGFR2-C + NK
FGFR2-D + NK
FGFR2-B/C + NK
FGFR2-B/D + NK

S

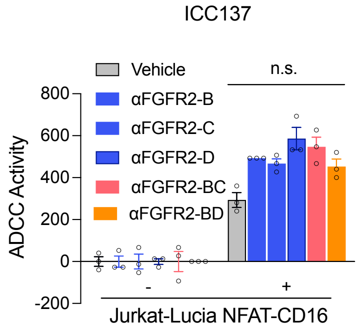
ICC137
Vehicle
αFGFR2-B
αFGFR2-C
αFGFR2-D
αFGFR2-BC
αFGFR2-BD
n.s.
ADCC Activity
800
600
400
200
0
-200
-
+
Jurkat-Lucia NFAT-CD16

T

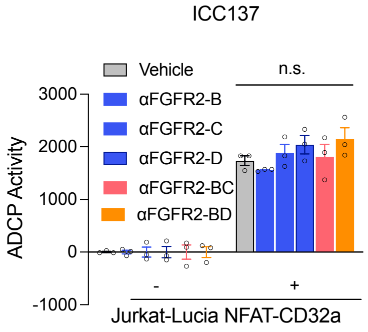
ICC137
Vehicle
αFGFR2-B
αFGFR2-C
αFGFR2-D
αFGFR2-BC
αFGFR2-BD
n.s.
ADCP Activity
3000
2000
1000
0
-1000
-
+
Jurkat-Lucia NFAT-CD32a

Supplemental Figure 5: Biparatopic antibodies show superior in vivo anti-tumor activity compared to the parental antibodies

(**A-B**) Plasma concentration–time profile of the biparatopic antibodies bpAb-B/D (**A**) and bpAb-B/C (**B**) in BALB/c scid mice with FGFR2-PGHDH expressing BaF3 xenografts treated at 5 mg/kg, 15 mg/kg, and 25 mg/kg. The plasma was collected at 1 h, 24 h, and 72 h post final administration of antibodies via intravenous tail vein injection. Data are mean ± SEM.

(**C-D**) Quantification of immunoblots in Figure 5E (**C**) and Figure 5F (**D**).

(**E-F**) Immunoblot analysis of ICC13-7 xenograft tumors (**E**) and its quantification (**F**). Tumors were harvested 5hrs after the final round of parental antibody B, D, C, or IgG1 administration at day 38 post initial treatment.

(**G-H**) Body weight (g) of BALB/c scid mice with FGFR2-PGHDH expressing BaF3 (**G**) or ICC13-7 (**H**) xenografts treated with parental antibody B, D, C, bpAb-B/C, bpAb-B/D, or IgG1 measured twice per week over the course of 25 days (BaF3) or 39 days (ICC13-7).

(**I**) Representative images of IHC staining for human IgG1 in ICC13-7 tumor xenografts treated with IgG1, parental antibodies B, D, C, biparatopic antibodies bpAb-B/C, bpAb-B/D at 10mg/kg. Images were taken at high magnification (10x, bottom panel) and low magnification (2x, top panel). Tissues were harvested at 5hr after final antibody administration. Scale bars, 200um.

(**J-K**) Representative images at 1x (upper panel) and 10x (lower panel) of IHC staining for mouse NKp46 in ICC13-7 tumor xenografts treated with IgG1, parental antibodies B, D, C, at 10mg/kg and biparatopic antibodies bpAb-B/C, bpAb-B/D at 10 and 30 mg/kg (**J**). Quantification of mNKp46+ staining per images (n>10 per treatment) (**K**).

(**L**) Differential expressed murine genes in bpAb-B/C or bpAb-B/D, compared to IgG1 treatment control. Genes that are significantly upregulated (red) or downregulated (green) with LogFC>0.5 and FDR<0.05 are shown. Upregulated genes involved in immune functions are shown in the table.

(**M**) Tumors of BALB/c scid mice harboring ICC13-7 subcutaneous xenografts treated with parental and biparatopic antibodies used for RNA sequencing analysis.

(**N-Q**) Differential expressed genes of bpAb-B/C or bpAb-B/D, compared to IgG1treated tumors according to each gene sets. Gene Ontology sets include NK cells mediated immunity, ADCC, Fc receptor mediated stimulatory signaling pathway, macrophage activation involved in immune response, and complement receptor mediated signaling pathway.

(**R**) NK killing assay showing % dead/live cells area curves for 48 h of B, D, C, bpAb-B/C, bpAb-B/D, or IgG1 treated ICC13-7 cells with or without NK cells using incucyte.

(**S-T**) Jurkat-Lucia cells overexpressing CD16 or CD32a with NFAT reporter used to assess the ADCC (**S**) or ADCP (**T**) respectively in B, D, C, bpAb-B/C, bpAb-B/D, or IgG1 treated ICC13-7 cells.

All data are mean ± SEM. Data are representative of one out of two independent experiments. ns=not significant, *P < 0.05, **P < 0.01, ***P < 0.001, ****P < 0.0001 by One-way ANOVA multiple comparisons.

Supplemental Figure. 6

A

IgG1 B/D

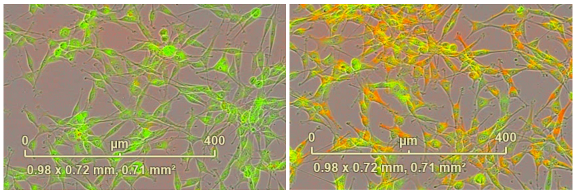

Green: Lysotracker
Red: Incucyte Fabfluor Red

NIH3T3: FGFR2-PHGDH

B

1:1

SEC-MALS

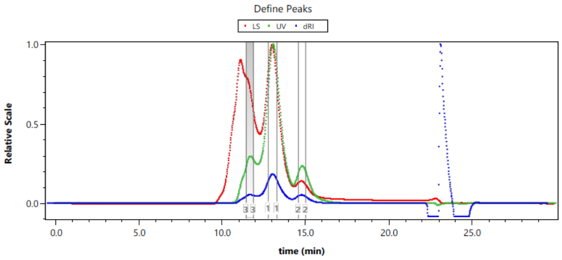

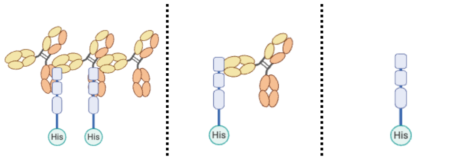

Peaks	#3	#1	#2
Size (kD)	579.1	219.8	117.2
Ab: ECD	~3:2	~1:1	~0:1, 0:2
	(453:124=577)	(151:62 =213)	(62, 124)

1:3

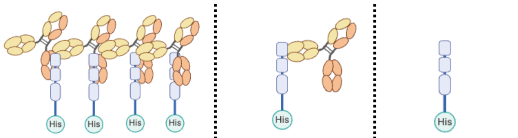

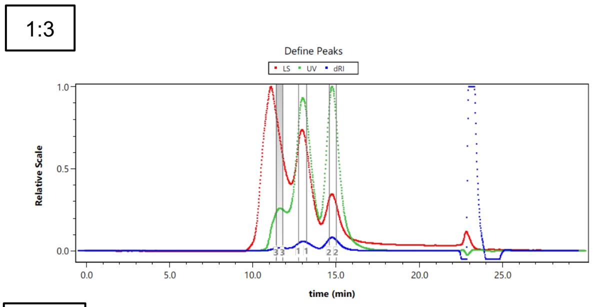

Peaks	#3	#1	#2
Size (kD)	845.6	249.6	84.5
Ab: ECD	4:4	~1:1	~0:1
	(604:250=854)	(151:62=213)	(62.5)

1:5

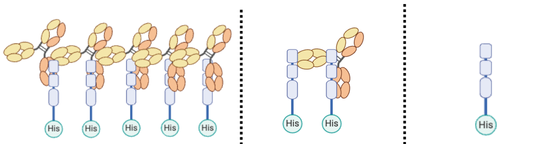

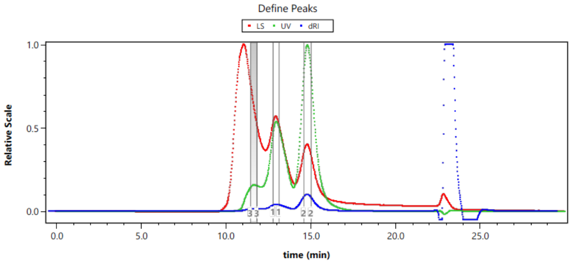

Peaks	#3	#1	#2
Size (kD)	1092	292.5	81.51
Ab: ECD	5:5	~1:2	~0:1
	(735:375=1065)	(151:124=275)	(62.5)

C

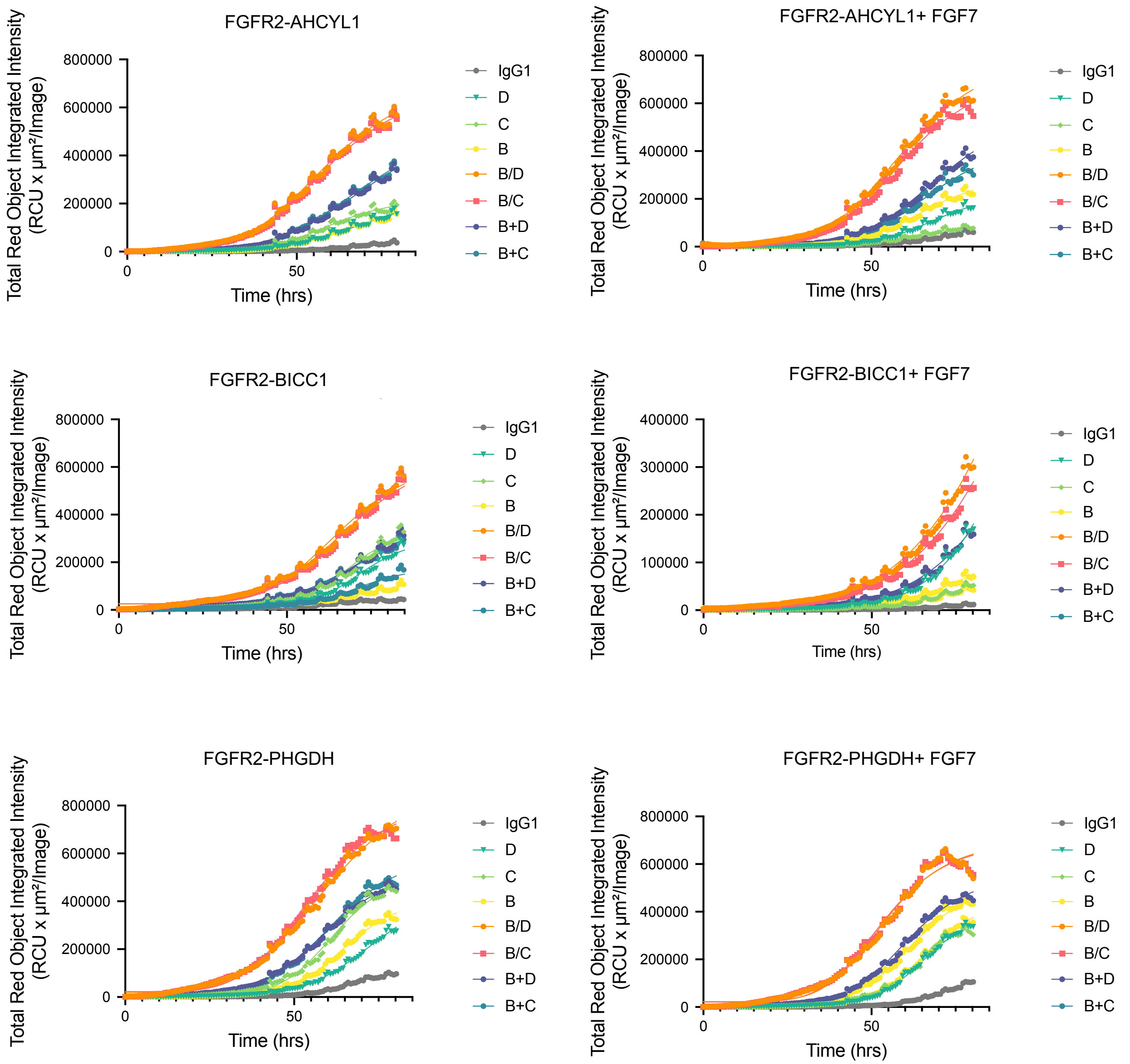

Fig. 6J WB quantification

D

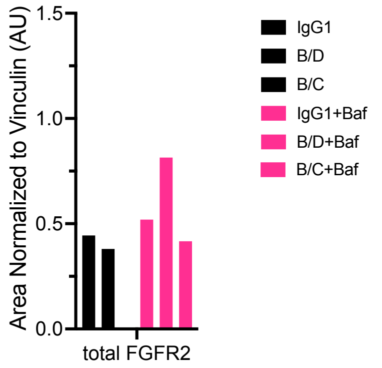

Supplemental Figure 6: The biparatopic antibodies promote receptor internalization and lysosomal degradation

(**A**) Representative images of NIH3T3 cells expressing FGFR2-PHGDH incubated with Fabfluor-pH labeled bpAb-B/D (red) and lysotracker labeled lysosomes (green). Colocalization between Fab fluorophore and lysotracker was shown in yellow.

(**B**) SEC-MALS analysis of antibody: ECD complexes with increasing ratio 1:1, 1:3, 1:5. As ECD increases, the sizes of complex formed increases as illustrated by the cartoon models above the table.

(**C**) Quantification of internalization/degradation signals in FGFR2-ACHYL1, FGFR2-BICC1, FGFR2-PHGDH expressing NIH3T3 cells treated with parental antibodies B, D, C, biparatopic antibody bpAb-B/C, bpAb-B/D, B+D or B+C from 0-80 h post incubation with or without ligand FGF7.

(**D**) Quantification of immunoblot in Figure 6J.

Supplemental Figure. 7

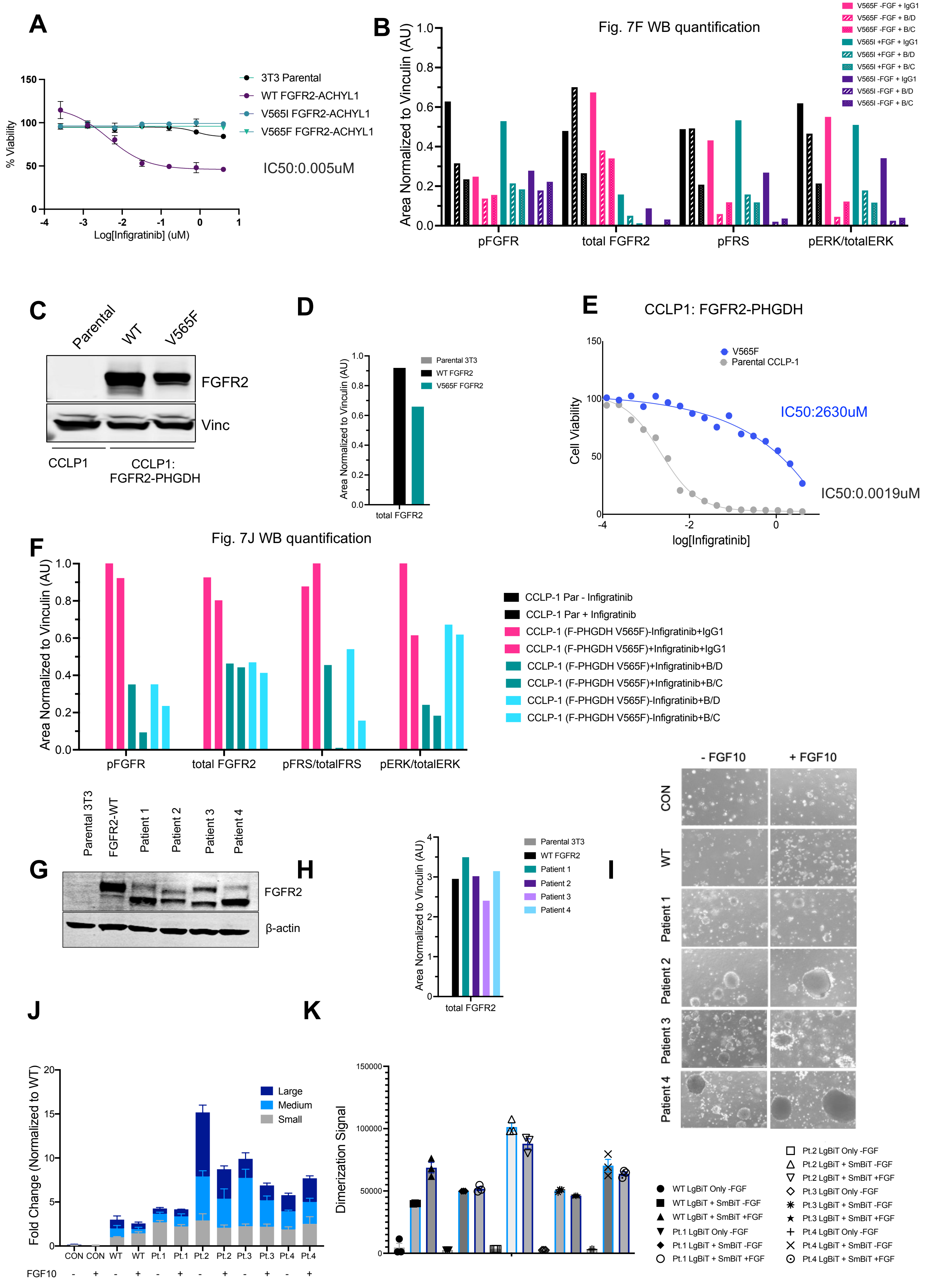

L

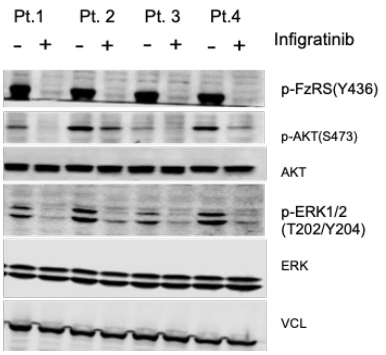

M

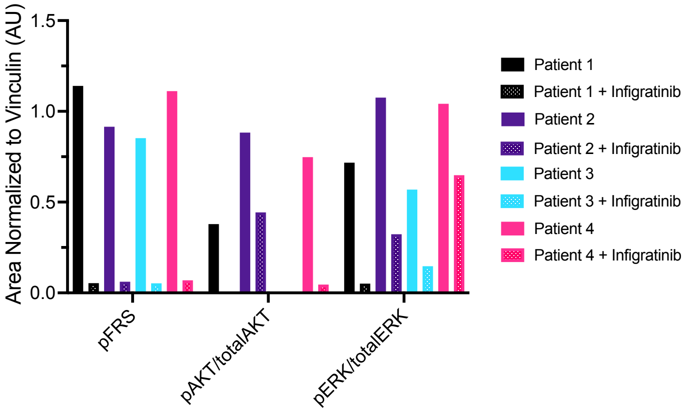

O

Fig. 7M WB quantification

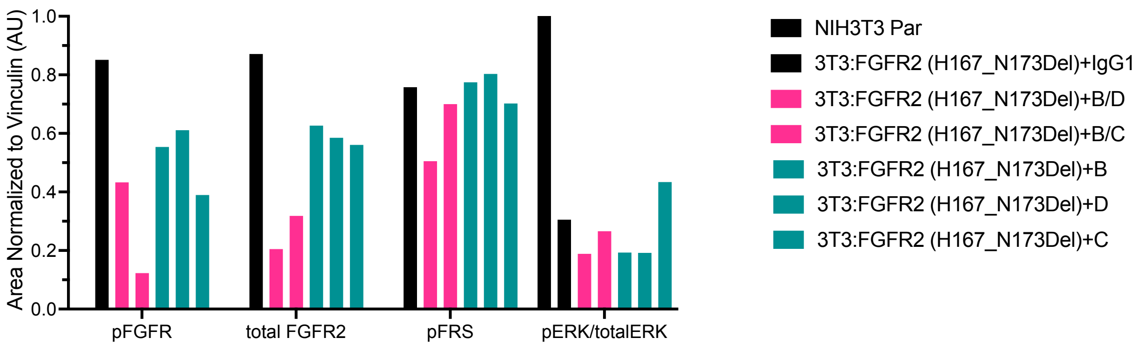

P

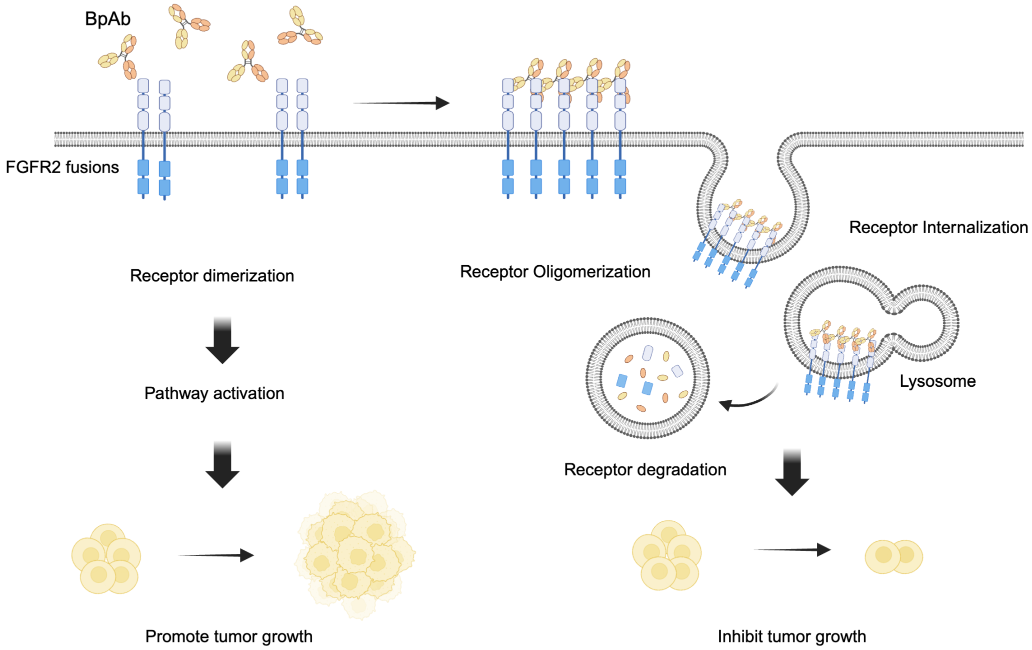

Supplemental Figure 7: Combinations of biparatopic antibodies with FGFR inhibitors

(**A**) IC50 values of NIH3T3 cells expressing FGFR2-ACHYL1 with kinase mutation V565F or V565I upon treatment with Infigratinib.

(**B**) Quantification of immunoblot in Figure 7F.

(**C-D**) Immunoblot analysis of CCLP-1 cell line expressing FGFR2-PHGDH WT, or resistant mutation V565F (**C**) and its quantification (**D**).

(E) IC50 values of CCLP-1 cells expressing FGFR2-PHGDH with kinase mutation V565F upon treatment with Infigratinib from 0-4uM.

(**F**) Quantification of immunoblot in Figure 7J.

(**G-H**) Immunoblot analysis showing levels of FGFR2 in NIH3T3 cell line expressing FGFR2-WT, or oncogenic FGFR2 mutants derived from patient 1, 2, 3, and 4 (**G**) and its quantification (**H**).

(**I-J**) Representative images of soft agar assays showing higher number of colonies in patient 1-4 derived - mutations overexpressing NIH3T3 cells compared to parental cells and FGFR2-WT overexpressing cell line controls. (**I**) Quantification of number of transforming colonies (relative to that of FGFR2-WT) in patient-derived mutations overexpressing cells. Number of colonies were quantified and categorized based on their sizes (**J**).

(**K**) NanoBiT data showing increased FGFR2 receptor dimerization signals in HEK293T cells expressing patient-derived mutation constructs compared to WT controls at base line.

(**L-M**) Immunoblot analysis showing phosphorylation levels of FGFR2 downstream effectors in patient 1-4 derived FGFR2 ECD deletion mutants with and without treatment with FGFR inhibitor infigratinib at 0.5uM (**L**) and its quantification (**M**).

(**O**) Quantification of immunoblot in Figure 7M.

(**P**) Illustrative representation of the proposed mechanism of action for our biparatopic antibody. In FGFR2 driven tumors, oncogenic FGFR2 spontaneous dimerize and activate the downstream pathway. Biparatopic antibodies bind FGF receptor intermolecularly crosslinking receptors to form large complexes which then trigger internalization and degradation of oncogenic FGFR2 to inhibit tumor growth.

Supplementary Table. 1

Antibody	VH	VL
A	EVQLLESGGGLVQPGGSLRLSCAASGFTFSSYAMSWVRQAPGKGLEWVSAISGSGTSTYYADSVKGRFTISRDNSKNTLYLQMNSLRAEDTAVYYCARVRYNWNHGDWFDPWGQGTLVTVSS	QSVLTQPPSASGTPGQRVTISCSGSSSNIGNNYVSWYQQLPGTAPKLLIYENYNRPAGVPDRFSGSKSGTSASLAISGLRSEDEADYYCSSWDDSLNYWVFGGGTKLTVLG
B	QIQLVQSGPELKKPGETVKISCKASGYTFTDFGMNWMKQAPGKGFKWMGWINTSTGESTYADDFKGRFAFSLETSASTAYLQINNLKNEDMATYFCARNSYYGGSYGYWGQGTTLTVSS	DIVMSQSPSSLAVSVGEKVTMKCKSSQSLLYSSNQKNYLAWYQQKPGQSPKLLIYWASTRESGVPDRFTGSGSGTDFTLTISSVKAEDLAVYYCQQYYSYPWTFGGGTKLEIK
C	QVQLVESGGGLVKPGGSLRLSCAASGFTFSSYALSWVRQAPGKGLEWVGRIRSKIDGGTTDYAAPVKGRFTISRDDSKNTLYLQMNSLKTEDTAVYYCARDRSPSDSSAFAIWGQGTLVTVSS	DIELTQPPSVSVSPGQTASITCSGDNLGSQYVDWYQQKPGQAPVLVIYDDNDRPSGIPERFSGSNSGNTATLTISGTQAEDEADYYCQSWDSLSVVFGGGTKLTVLG
D	EVQLVESGGGLVQPGGSLRLSCAASGFPFTSTGISWVRQAPGKGLEWVGRTHLGDGSTNYADSVKGRFTISADTSKNTAYLQMNSLRAEDTAVYYCARTYGIYDTYDMYTEYVMDYWGQGTLVTVSS	DIQMTQSPSSLSASVGDRVTITCRASQDVDTSLAWYKQKPGKAPKLLIYSASFLYSGVPSRFSGSGSGTDFTLTISSLQPEDFATYYCQQSTGHPQTFGQGTKVEIK
E	QVQLVQSGAEVKKPGSSVKVSCKASGYIFTTYNVHWVRQAPGQGLEWIGSIYPDNGDTSYNQNFKGRATITADKSTSTAYMELSSLRSEDTAVYYCARGDFAYWGQGTLVTVSS	DIQMTQSPSSLSASVGDRVTITCKASQGVSNDYAWYQQKPGKAPKLLIYSASYRYTGVPSRFSGSGSGTDFTFTISSLQPEDIATYYCQQHSTTPYTFGQGTKLEIK
F	QVQLVQSGAEVKKPGESLKISCKGSGYSFTNYYIHWVRQMPGKGLEWMGAIYPDNSDTTYSPSFQGQVTISADKSISTAYLQWSSLKASDTAMYYCARGADIWGQGTLVTVSS	DIQMTQSPSSLSASVGDRVTITCRASQDIDPYLSNWYQQKPGKAPKLLIYDASNLQSGVPSRFSGSGSGTDFTLTISSLQPEDFATYYCQQTTSHPYTFGQGTKVEIK
