# Supplementary material for: Identification of potent biparatopic antibodies targeting FGFR2 fusion–driven cholangiocarcinoma
Source: J Clin Invest. 2025 Feb 27;135(8):e182417. doi: 10.1172/JCI182417 (PMC11996885; doi:10.1172/JCI182417)
Supplement: Unedited blot and gel images [file jci-135-182417-s163.pdf]

Full unedited gel for Figure 1J

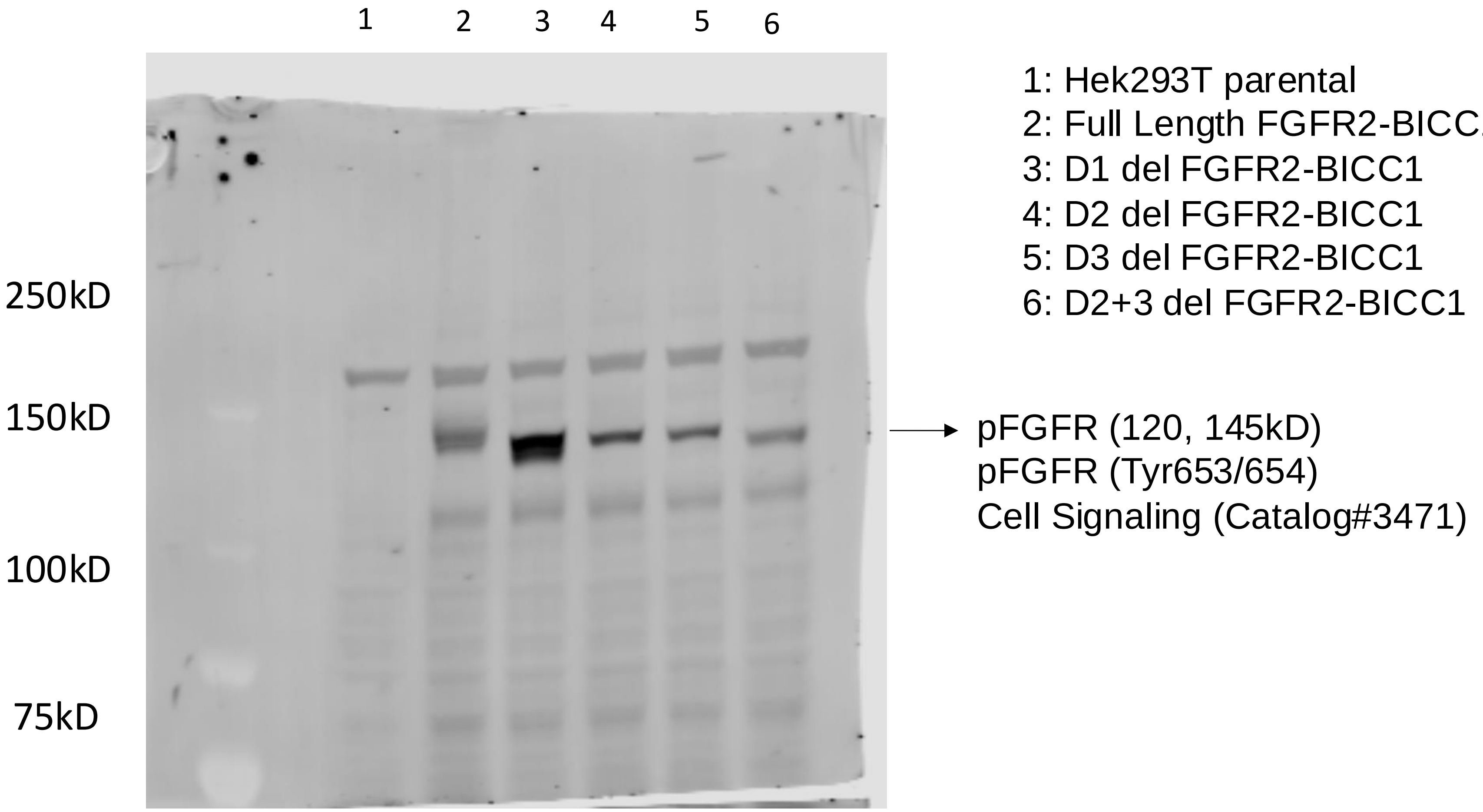

1: Hek293T parental
2: Full Length FGFR2-BICC1
3: D1 del FGFR2-BICC1
4: D2 del FGFR2-BICC1
5: D3 del FGFR2-BICC1
6: D2+3 del FGFR2-BICC1

Full unedited gel for Figure 1J

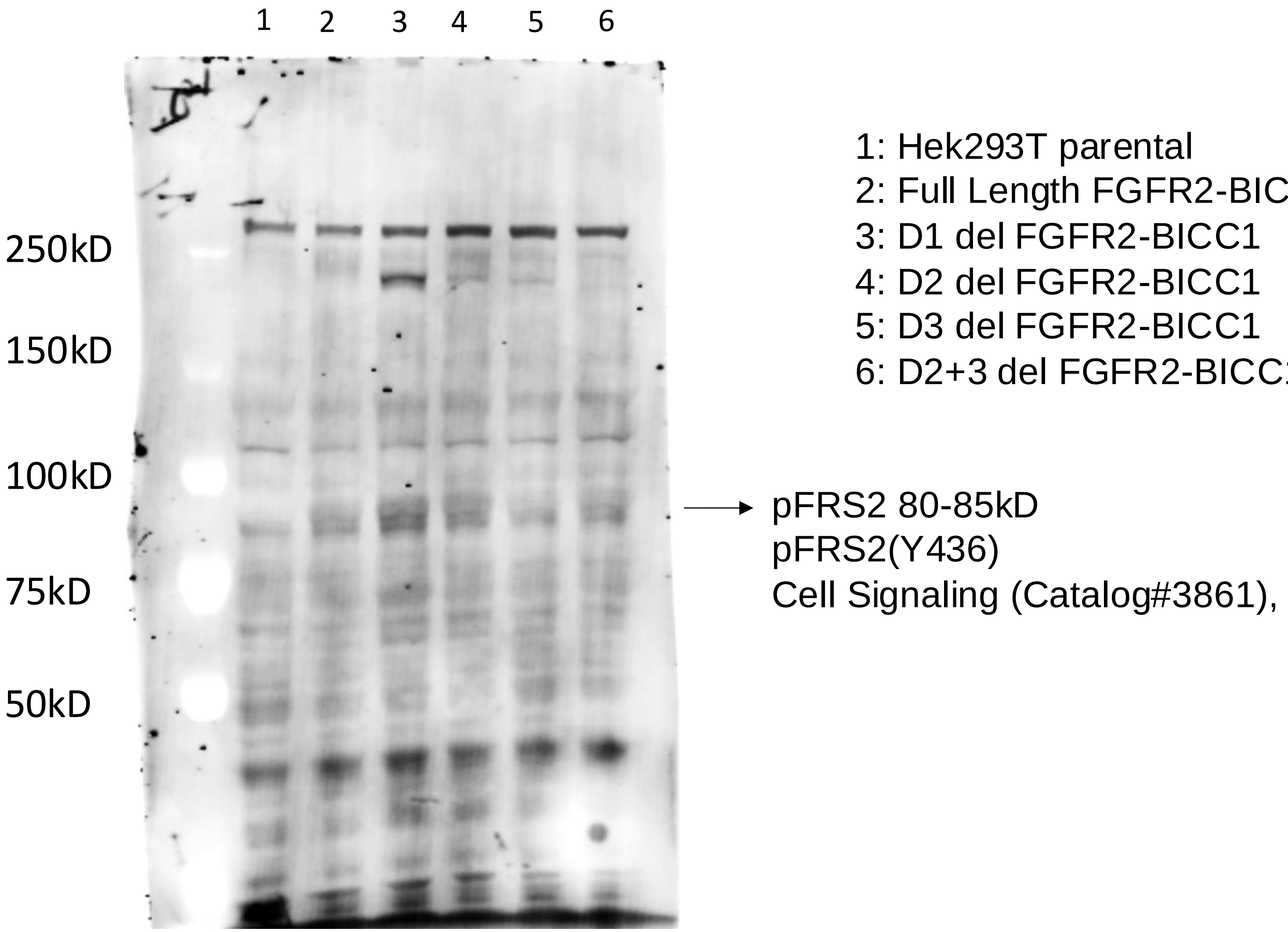

1: Hek293T parental
2: Full Length FGFR2-BICC1
3: D1 del FGFR2-BICC1
4: D2 del FGFR2-BICC1
5: D3 del FGFR2-BICC1
6: D2+3 del FGFR2-BICC1

Full unedited gel for Figure 1J

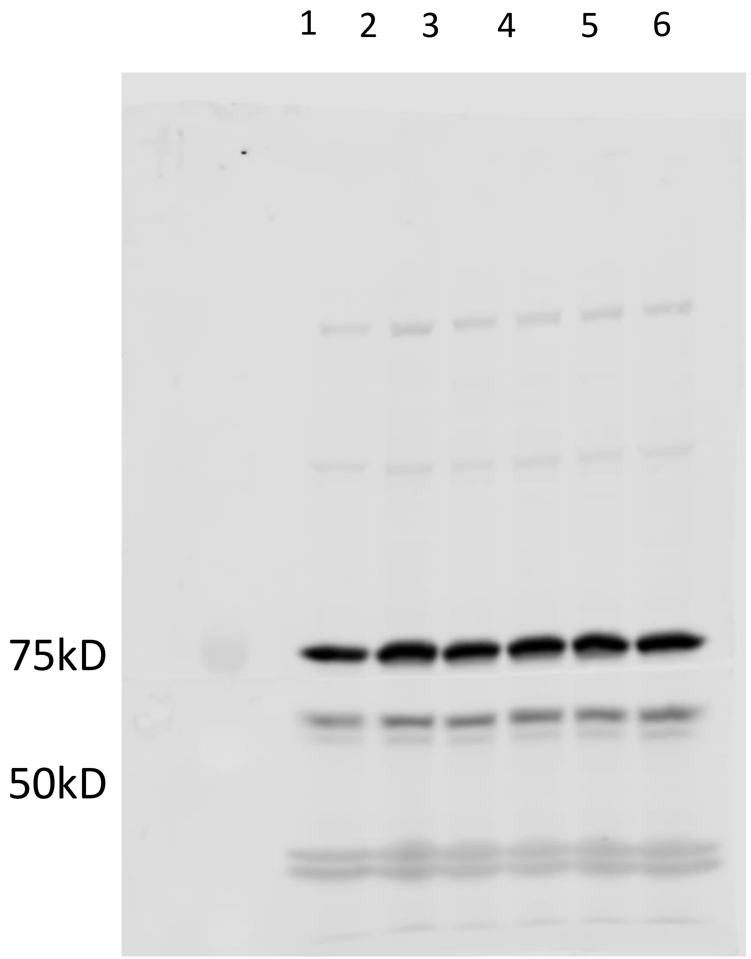

1: Hek293T parental
2: Full Length FGFR2-BICC1
3: D1 del FGFR2-BICC1
4: D2 del FGFR2-BICC1
5: D3 del FGFR2-BICC1
6: D2+3 del FGFR2-BICC1

tFRS2 80-85kD
FRS2(Abcam, Catalog#ab183492)

Full unedited gel for Figure 1J

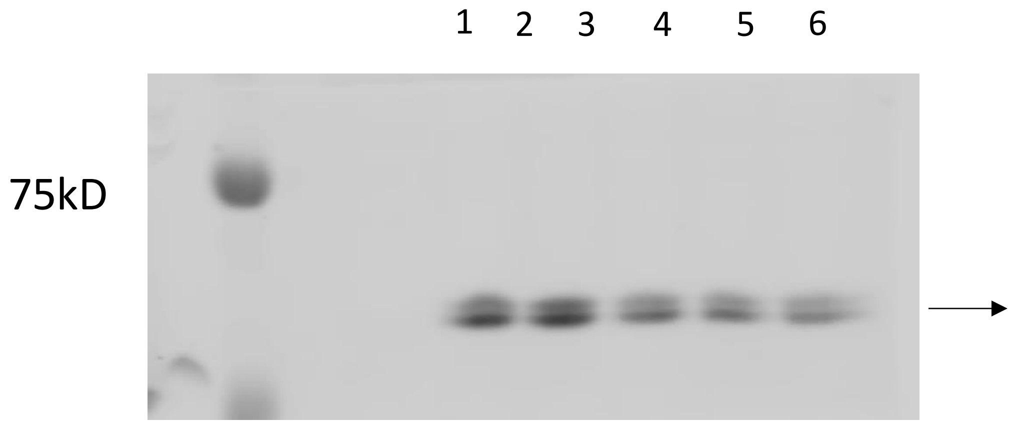

Phospho-p44/42 MAPK (Erk1/2) 45kD
(Thr202/Tyr204)
Cell Signaling Catalog#9106

1: Hek293T parental
2: Full Length FGFR2-BICC1
3: D1 del FGFR2-BICC1
4: D2 del FGFR2-BICC1
5: D3 del FGFR2-BICC1
6: D2+3 del FGFR2-BICC1

Full unedited gel for Figure 1J

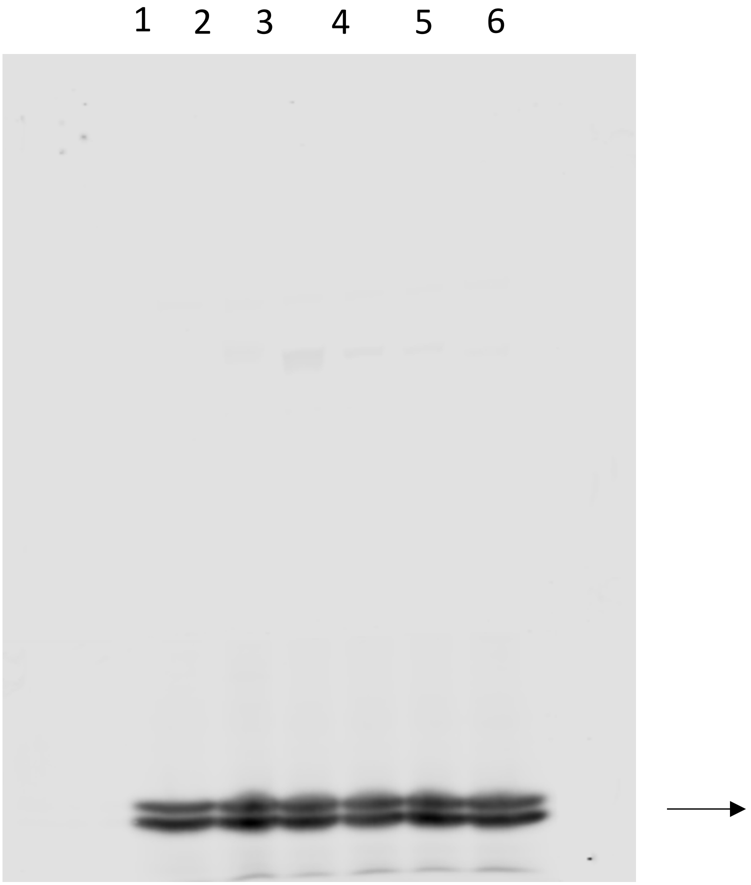

1: Hek293T parental
2: Full Length FGFR2-BICC1
3: D1 del FGFR2-BICC1
4: D2 del FGFR2-BICC1
5: D3 del FGFR2-BICC1
6: D2+3 del FGFR2-BICC1

p44/42 MAPK (Erk1/2) 42, 44kD
Cell Signaling Catalog#4695

Full unedited gel for Figure 1J

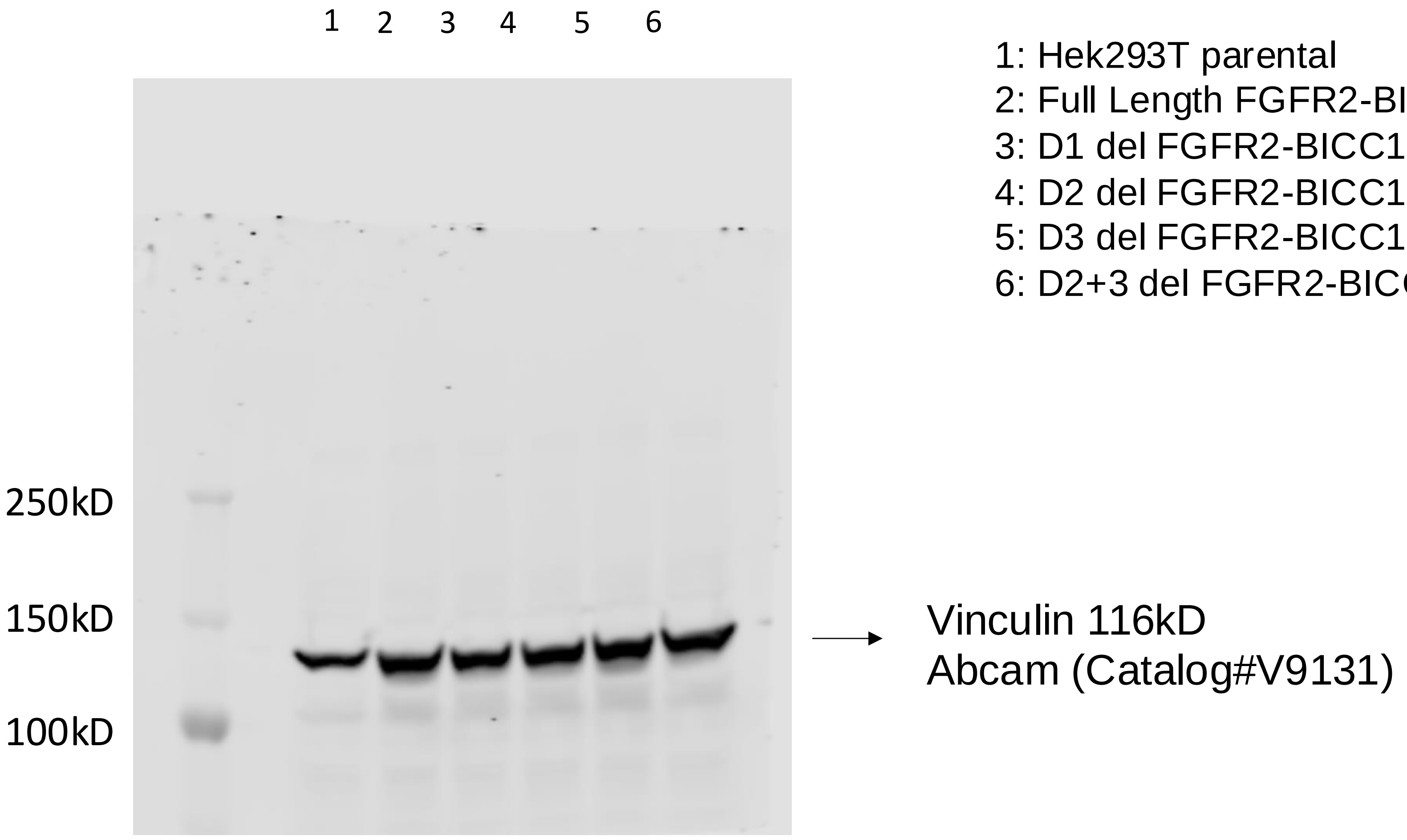

1: Hek293T parental
2: Full Length FGFR2-BICC1
3: D1 del FGFR2-BICC1
4: D2 del FGFR2-BICC1
5: D3 del FGFR2-BICC1
6: D2+3 del FGFR2-BICC1

Full unedited gel for Figure S1B

1: NIH3T3 parental
2: NIH3T3 parental
3: pLenti-CMV-FGFR2-LgBiT expressing 3T3 (DNA:TrasnIT=1:2)
4: pLenti-CMV-FGFR2-LgBiT expressing 3T3(DNA:TrasnIT=1:3)
5: pLenti-CMV-FGFR2-LgBiT expressing 3T3 (DNA:TrasnIT=1:4)
6: pLenti-CMV-FGFR2-ACHYL1-LgBiT expressing 3T3 (DNA:TrasnIT=1:2)
7: pLenti-CMV-FGFR2-ACHYL1-LgBiT expressing 3T3(DNA:TrasnIT=1:3)
8: pLenti-CMV-FGFR2-ACHYL1-LgBiT expressing 3T3 (DNA:TrasnIT=1:4)
9: pLenti-CMV-FGFR2-BICC1-LgBiT expressing 3T3 (DNA:TrasnIT=1:2)
10: pLenti-CMV-FGFR2-BICC1 expressing 3T3(DNA:TrasnIT=1:3)
11: pLenti-CMV-FGFR2-BICC1 expressing 3T3 (DNA:TrasnIT=1:4)

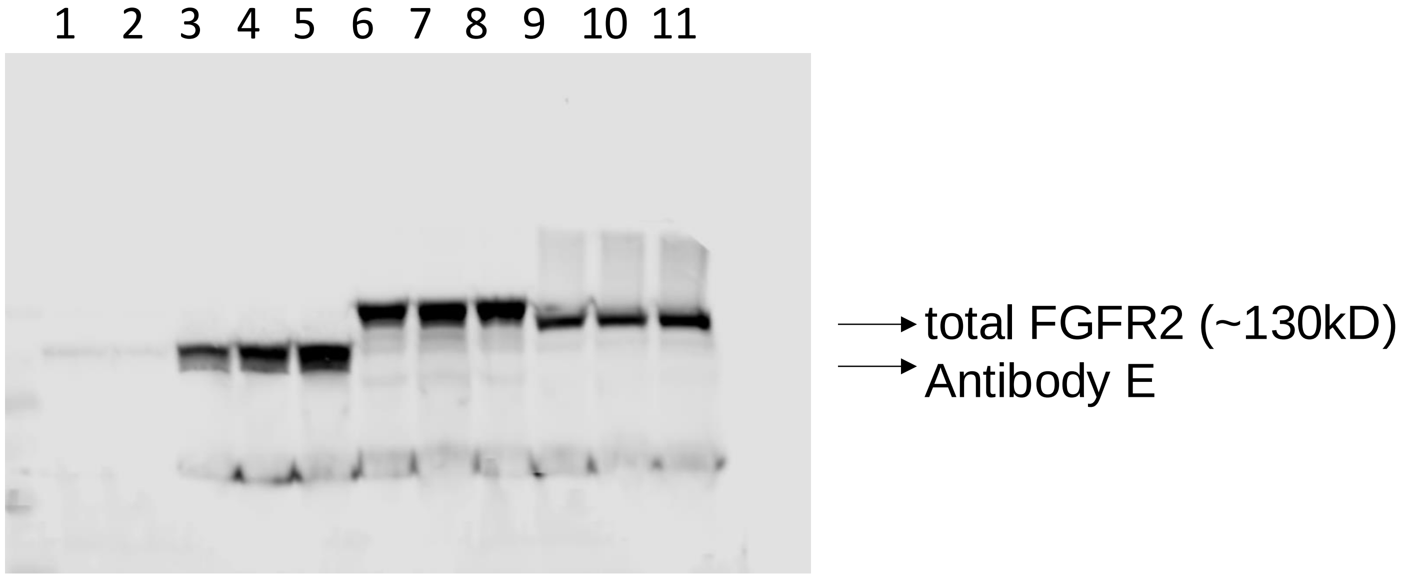

Full unedited gel for Figure S1B

1: NIH3T3 parental
2: NIH3T3 parental
3: pLenti-CMV-FGFR2-LgBiT expressing 3T3 (DNA:TrasnIT=1:2)
4: pLenti-CMV-FGFR2-LgBiT expressing 3T3(DNA:TrasnIT=1:3)
5: pLenti-CMV-FGFR2-LgBiT expressing 3T3 (DNA:TrasnIT=1:4)
6: pLenti-CMV-FGFR2-ACHYL1-LgBiT expressing 3T3 (DNA:TrasnIT=1:2)
7: pLenti-CMV-FGFR2-ACHYL1-LgBiT expressing 3T3(DNA:TrasnIT=1:3)
8: pLenti-CMV-FGFR2-ACHYL1-LgBiT expressing 3T3 (DNA:TrasnIT=1:4)
9: pLenti-CMV-FGFR2-BICC1-LgBiT expressing 3T3 (DNA:TrasnIT=1:2)
10: pLenti-CMV-FGFR2-BICC1 expressing 3T3(DNA:TrasnIT=1:3)
11: pLenti-CMV-FGFR2-BICC1 expressing 3T3 (DNA:TrasnIT=1:4)

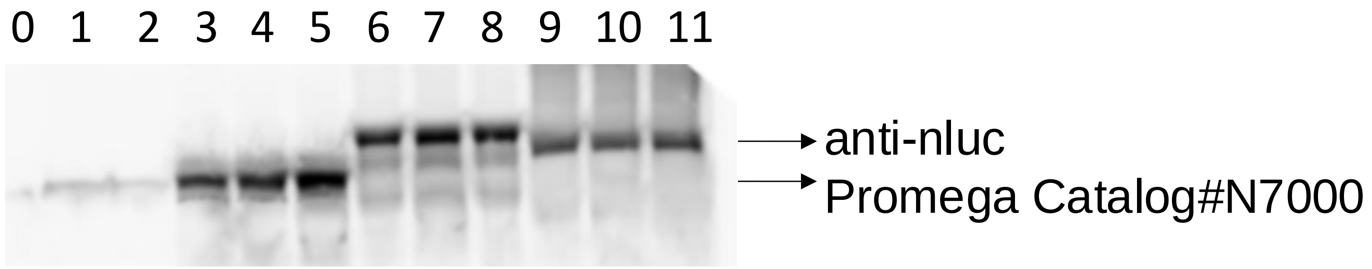

Full unedited gel for Figure S1B

1: NIH3T3 parental
2: NIH3T3 parental
3: pLenti-CMV-FGFR2-LgBiT expressing 3T3 (DNA:TrasnIT=1:2)
4: pLenti-CMV-FGFR2-LgBiT expressing 3T3(DNA:TrasnIT=1:3)
5: pLenti-CMV-FGFR2-LgBiT expressing 3T3 (DNA:TrasnIT=1:4)
6: pLenti-CMV-FGFR2-ACHYL1-LgBiT expressing 3T3 (DNA:TrasnIT=1:2)
7: pLenti-CMV-FGFR2-ACHYL1-LgBiT expressing 3T3(DNA:TrasnIT=1:3)
8: pLenti-CMV-FGFR2-ACHYL1-LgBiT expressing 3T3 (DNA:TrasnIT=1:4)
9: pLenti-CMV-FGFR2-BICC1-LgBiT expressing 3T3 (DNA:TrasnIT=1:2)
10: pLenti-CMV-FGFR2-BICC1 expressing 3T3(DNA:TrasnIT=1:3)
11: pLenti-CMV-FGFR2-BICC1 expressing 3T3 (DNA:TrasnIT=1:4)

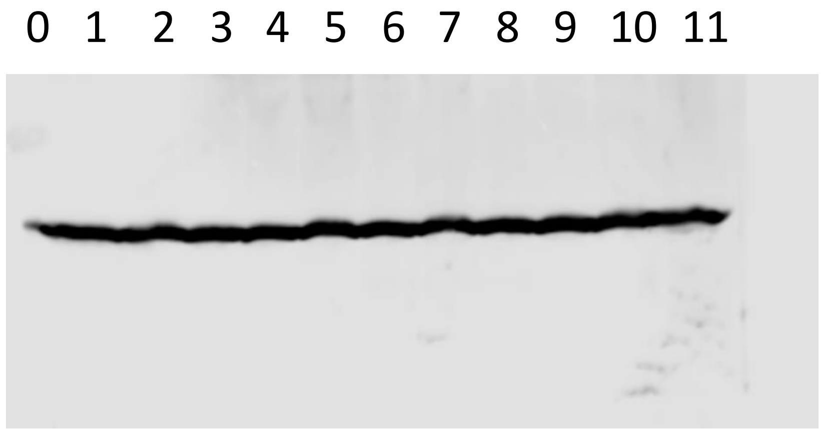

α-Tubulin, 52kD
Cell Signaling Catalog #3873

Full unedited gel for Figure S1D

1: Parental NIH3T3
2: NIH3T3: FGFR2-BICC1 FL
3: NIH3T3: FGFR2-BICC1 with D1 deletion
4: NIH3T3: FGFR2-BICC1 with D2 deletion
5: NIH3T3: FGFR2-BICC1 with D3 deletion
6: NIH3T3: FGFR2-BICC1 with D2+3 deletion

1 2 3 4 5 6

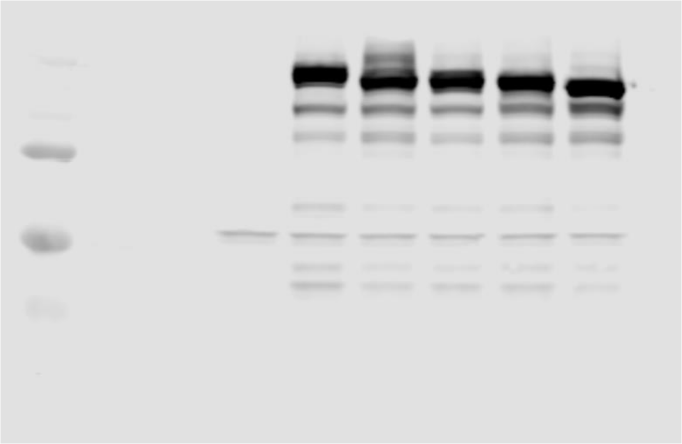

anti-nluc (FGFR2-BICC1)
Promega Catalog#N7000

Full unedited gel for Figure S1D

1: Parental NIH3T3
2: NIH3T3: FGFR2-BICC1 FL
3: NIH3T3: FGFR2-BICC1 with D1 deletion
4: NIH3T3: FGFR2-BICC1 with D2 deletion
5: NIH3T3: FGFR2-BICC1 with D3 deletion
6: NIH3T3: FGFR2-BICC1 with D2+3 deletion

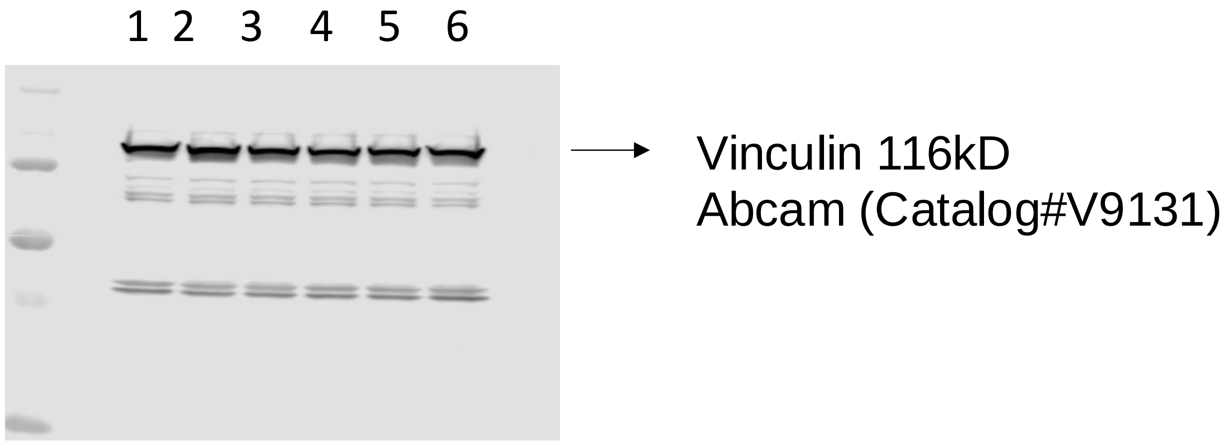

Full unedited gel for Figure 4G

0: ICC13-7 treated with PBS
1: ICC13-7 treated with IgG1 control
2: ICC13-7 treated with BpAb B/D
3: ICC13-7 treated with BpAb B/C
4: ICC13-7 treated with antibody B
5: ICC13-7 treated with antibody D
6: ICC13-7 treated with antibody C

0 1 2 3 4 5 6

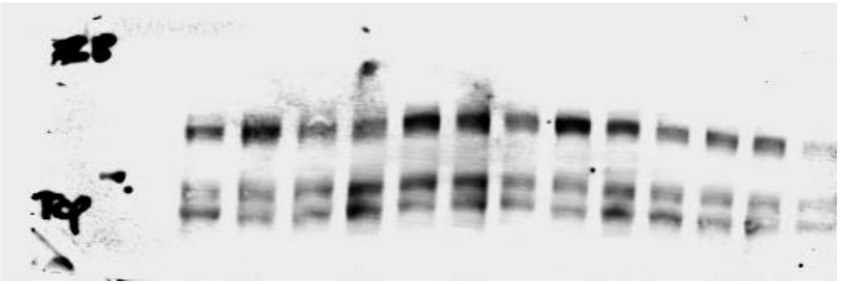

100kD

pFGFR (120, 145kD)
pFGFR (Tyr653/654)
Cell Signaling (Catalog#3471)

Full unedited gel for Figure 4G

0: ICC13-7 treated with PBS
1: ICC13-7 treated with IgG1 control
2: ICC13-7 treated with BpAb B/D
3: ICC13-7 treated with BpAb B/C
4: ICC13-7 treated with antibody B
5: ICC13-7 treated with antibody D
6: ICC13-7 treated with antibody C

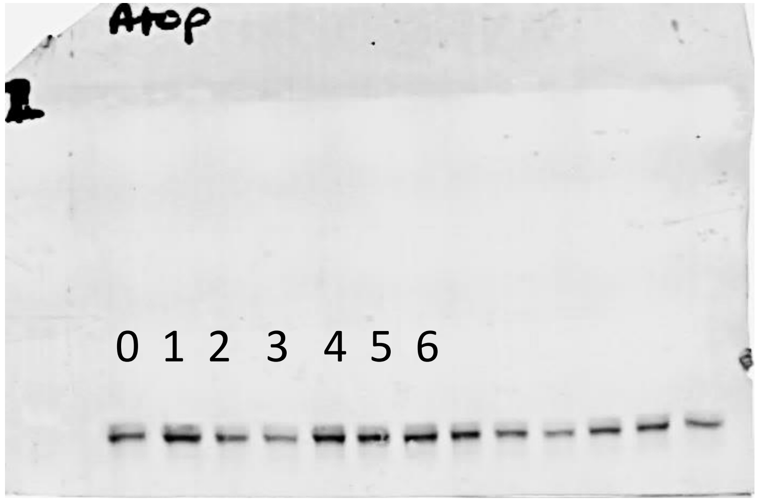

75kD

pFRS2 80-85kD
pFRS2(Y436)
Cell Signaling (Catalog#3861),

Full unedited gel for Figure 4G

0: ICC13-7 treated with PBS
1: ICC13-7 treated with IgG1 control
2: ICC13-7 treated with BpAb B/D
3: ICC13-7 treated with BpAb B/C
4: ICC13-7 treated with antibody B
5: ICC13-7 treated with antibody D
6: ICC13-7 treated with antibody C

0 1 2 3 4 5 6

75kD

55kD

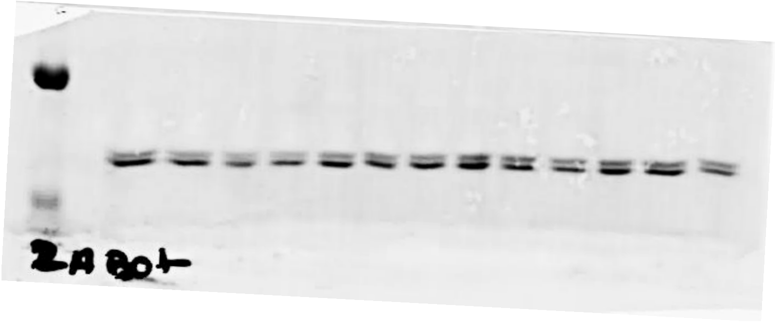

Phospho-p44/42 MAPK (Erk1/2) 45kD (Thr202/Tyr204)
Cell Signaling Catalog#9106

Full unedited gel for Figure 4G

0: ICC13-7 treated with PBS
1: ICC13-7 treated with IgG1 control
2: ICC13-7 treated with BpAb B/D
3: ICC13-7 treated with BpAb B/C
4: ICC13-7 treated with antibody B
5: ICC13-7 treated with antibody D
6: ICC13-7 treated with antibody C

0 1 2 3 4 5 6

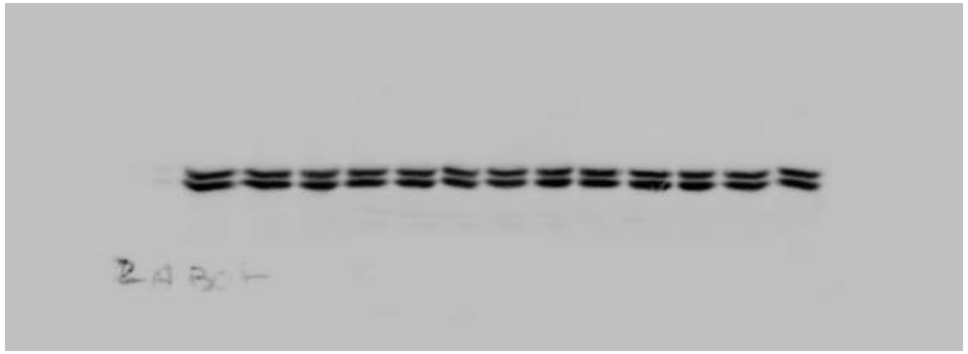

→ p44/42 MAPK (Erk1/2) 42, 44kD
Cell Signaling Catalog#4695

Full unedited gel for Figure 4G

0: ICC13-7 treated with PBS
1: ICC13-7 treated with IgG1 control
2: ICC13-7 treated with BpAb B/D
3: ICC13-7 treated with BpAb B/C
4: ICC13-7 treated with antibody B
5: ICC13-7 treated with antibody D
6: ICC13-7 treated with antibody C

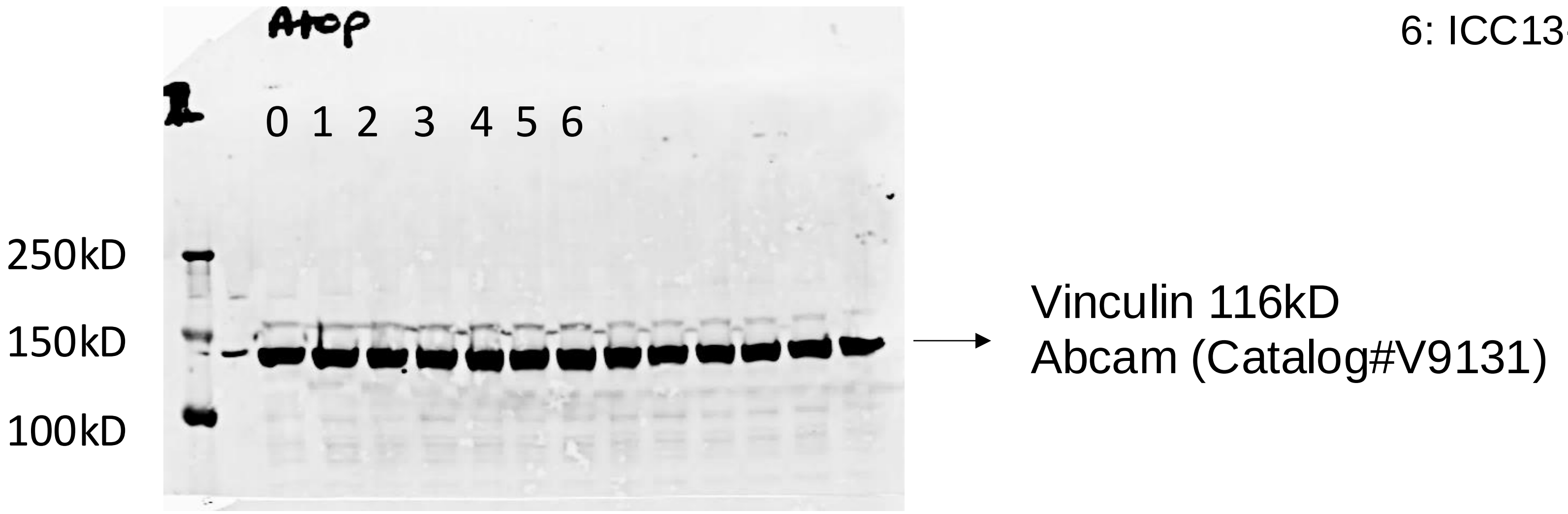

Full unedited gel for Figure 4H

1: ICC13-7 treated with IgG1 control
2: ICC13-7 treated with IgG1 control + FGF10
3: ICC13-7 treated with BpAb B/D + FGF10
4: ICC13-7 treated with BpAb B/C + FGF10
5: ICC13-7 treated with antibody B + FGF10
6: ICC13-7 treated with antibody D + FGF10
7: ICC13-7 treated with antibody C + FGF10

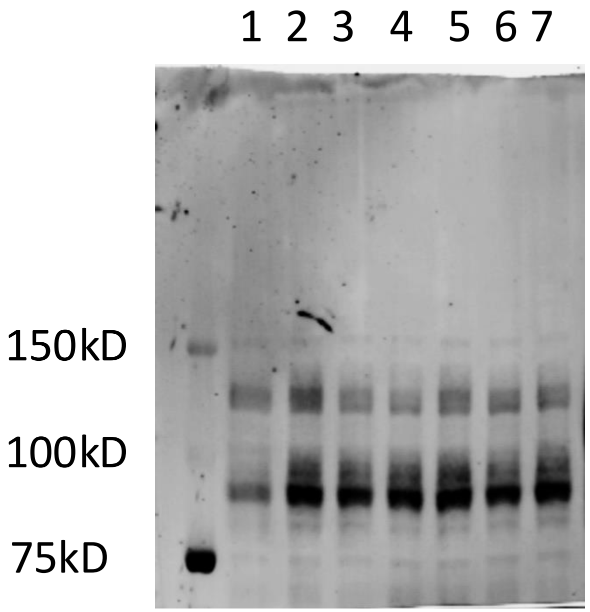

pFGFR (120, 145kD)
pFGFR (Tyr653/654)
Cell Signaling (Catalog#3471)

Full unedited gel for Figure 4H

1: ICC13-7 treated with IgG1 control
2: ICC13-7 treated with IgG1 control + FGF10
3: ICC13-7 treated with BpAb B/D + FGF10
4: ICC13-7 treated with BpAb B/C + FGF10
5: ICC13-7 treated with antibody B + FGF10
6: ICC13-7 treated with antibody D + FGF10
7: ICC13-7 treated with antibody C + FGF10

1 2 3 4 5 6 7

100kD

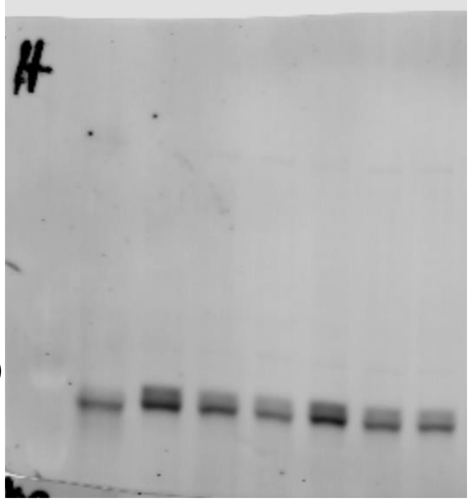

pFRS2 80-85kD
pFRS2(Y436)
Cell Signaling (Catalog#3861),

Full unedited gel for Figure 4H

1: ICC13-7 treated with IgG1 control
2: ICC13-7 treated with IgG1 control + FGF10
3: ICC13-7 treated with BpAb B/D + FGF10
4: ICC13-7 treated with BpAb B/C + FGF10
5: ICC13-7 treated with antibody B + FGF10
6: ICC13-7 treated with antibody D + FGF10
7: ICC13-7 treated with antibody C + FGF10

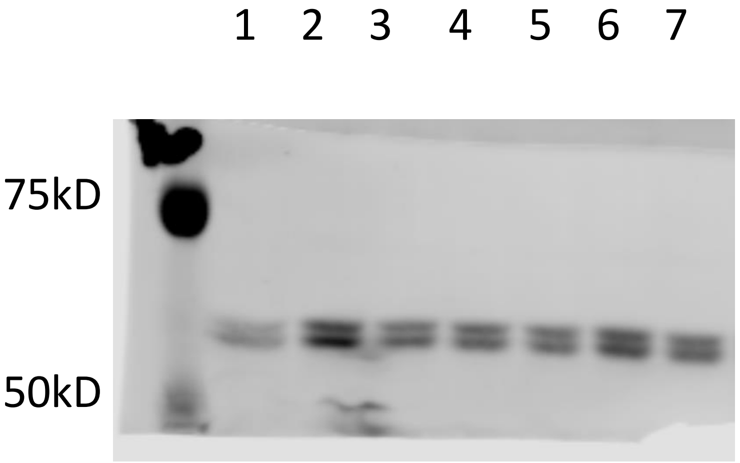

Phospho-p44/42 MAPK (Erk1/2) 45kD
(Thr202/Tyr204)
Cell Signaling Catalog#9106

Full unedited gel for Figure 4H

1: ICC13-7 treated with IgG1 control
2: ICC13-7 treated with IgG1 control + FGF10
3: ICC13-7 treated with BpAb B/D + FGF10
4: ICC13-7 treated with BpAb B/C + FGF10
5: ICC13-7 treated with antibody B + FGF10
6: ICC13-7 treated with antibody D + FGF10
7: ICC13-7 treated with antibody C + FGF10

1 2 3 4 5 6 7

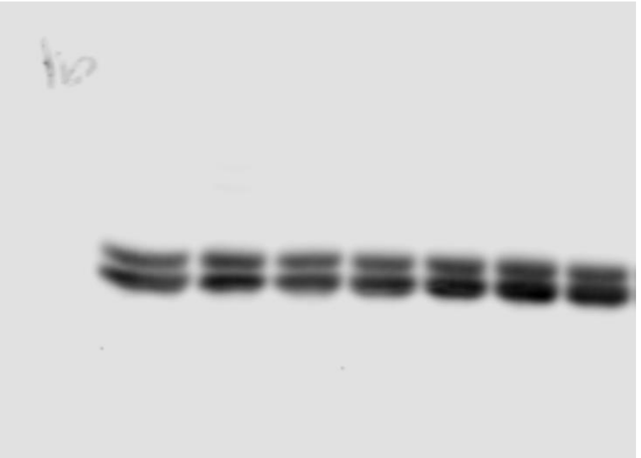

p44/42 MAPK (Erk1/2) 42, 44kD
Cell Signaling Catalog#4695

Full unedited gel for Figure 4H

1: ICC13-7 treated with IgG1 control
2: ICC13-7 treated with IgG1 control + FGF10
3: ICC13-7 treated with BpAb B/D + FGF10
4: ICC13-7 treated with BpAb B/C + FGF10
5: ICC13-7 treated with antibody B + FGF10
6: ICC13-7 treated with antibody D + FGF10
7: ICC13-7 treated with antibody C + FGF10

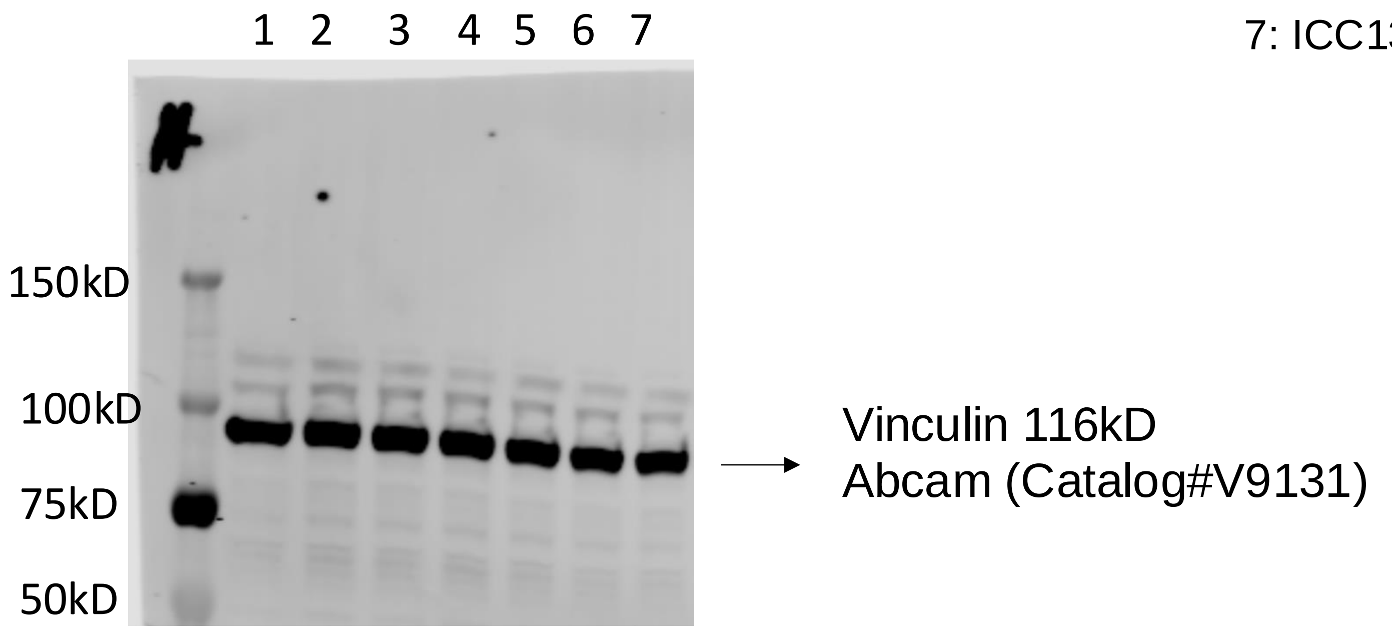

Full unedited gel for Figure S4A

1: ICC21 treated with IgG1 control
2: ICC21 treated with BpAb B/C
3: ICC21 treated with BpAb B/D
4: ICC21 treated with IgG1 control +FGF10
5: ICC21 treated with BpAb B/C + FGF10
6: ICC21 treated with BpAb B/D + FGF10

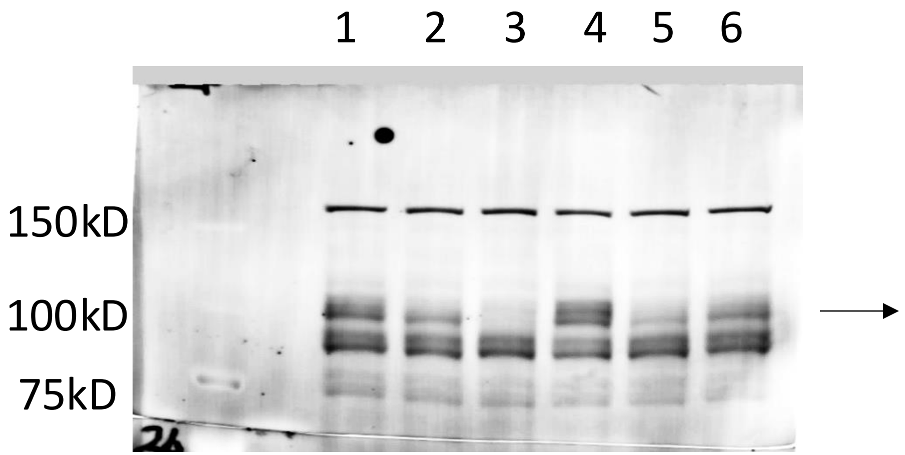

pFGFR (120, 145kD)
pFGFR (Tyr653/654)
Cell Signaling (Catalog#3471)

Full unedited gel for Figure S4A

1: ICC21 treated with IgG1 control
2: ICC21 treated with BpAb B/C
3: ICC21 treated with BpAb B/D
4: ICC21 treated with IgG1 control +FGF10
5: ICC21 treated with BpAb B/C + FGF10
6: ICC21 treated with BpAb B/D + FGF10

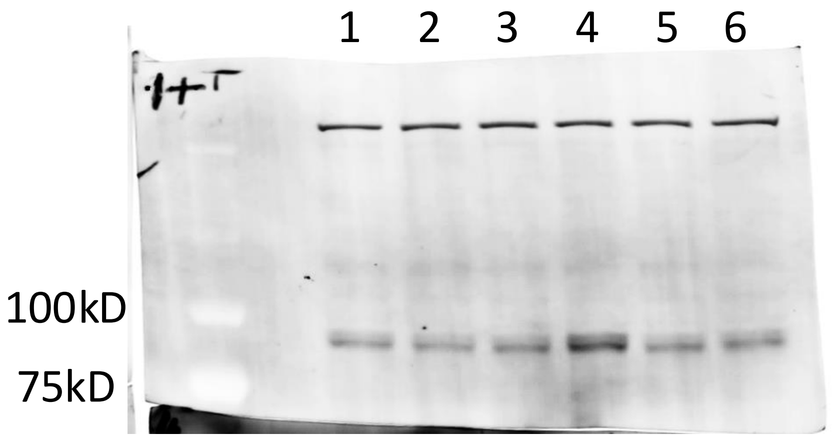

pFRS2 80-85kD
pFRS2(Y436)
Cell Signaling (Catalog#3861),

Full unedited gel for Figure S4A

1: ICC21 treated with IgG1 control
2: ICC21 treated with BpAb B/C
3: ICC21 treated with BpAb B/D
4: ICC21 treated with IgG1 control +FGF10
5: ICC21 treated with BpAb B/C + FGF10
6: ICC21 treated with BpAb B/D + FGF10

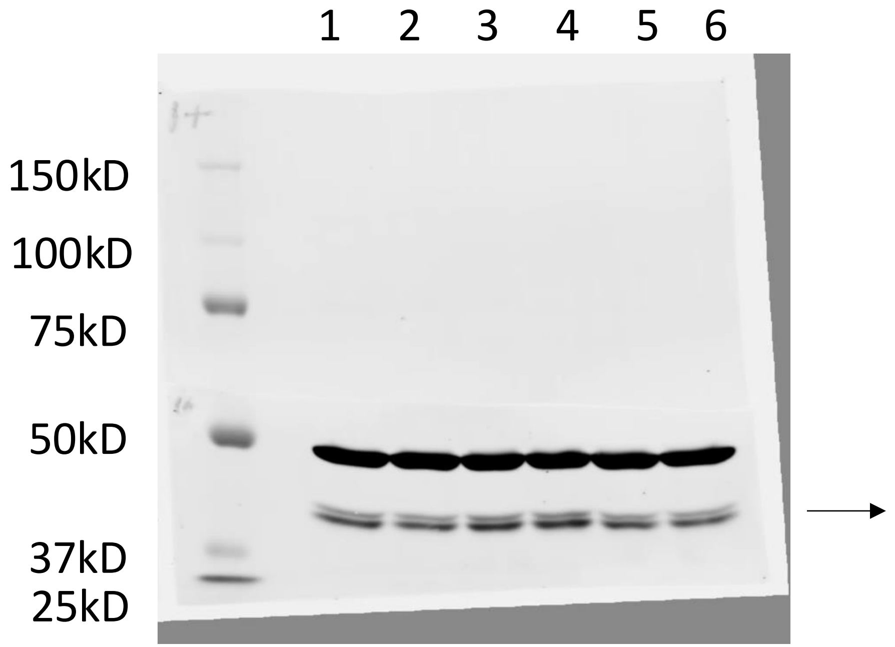

Full unedited gel for Figure S4A

1: ICC21 treated with IgG1 control
2: ICC21 treated with BpAb B/C
3: ICC21 treated with BpAb B/D
4: ICC21 treated with IgG1 control +FGF10
5: ICC21 treated with BpAb B/C + FGF10
6: ICC21 treated with BpAb B/D + FGF10

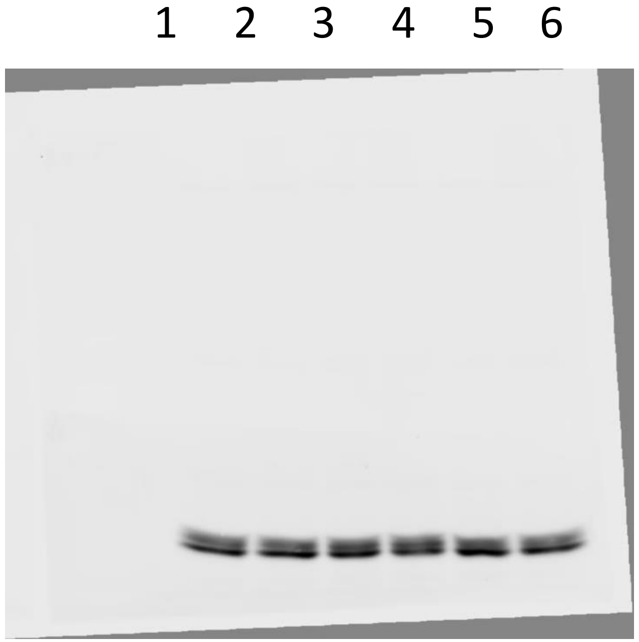

p44/42 MAPK (Erk1/2) 42, 44kD
Cell Signaling Catalog#4695

Full unedited gel for Figure S4A

1: ICC21 treated with IgG1 control
2: ICC21 treated with BpAb B/C
3: ICC21 treated with BpAb B/D
4: ICC21 treated with IgG1 control +FGF10
5: ICC21 treated with BpAb B/C + FGF10
6: ICC21 treated with BpAb B/D + FGF10

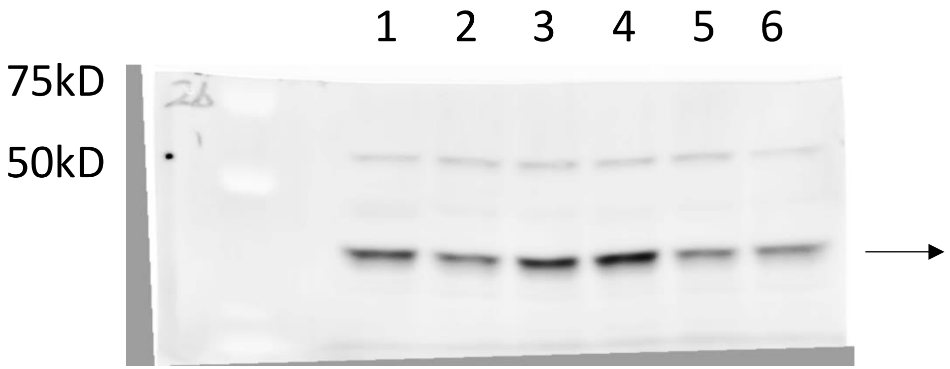

Phospho-MEK1/2 (Ser217/221) 45kD
Cell Signaling Catalog #9154

Full unedited gel for Figure S4A

1: ICC21 treated with IgG1 control
2: ICC21 treated with BpAb B/C
3: ICC21 treated with BpAb B/D
4: ICC21 treated with IgG1 control +FGF10
5: ICC21 treated with BpAb B/C + FGF10
6: ICC21 treated with BpAb B/D + FGF10

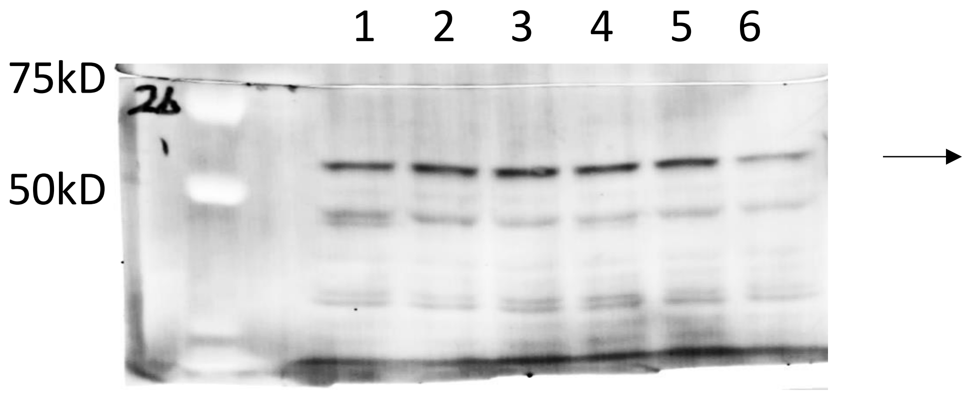

Phospho-Akt (Ser473), 60kD
Cell Signaling Catalog #4060

Full unedited gel for Figure S4A

1: ICC21 treated with IgG1 control
2: ICC21 treated with BpAb B/C
3: ICC21 treated with BpAb B/D
4: ICC21 treated with IgG1 control +FGF10
5: ICC21 treated with BpAb B/C + FGF10
6: ICC21 treated with BpAb B/D + FGF10

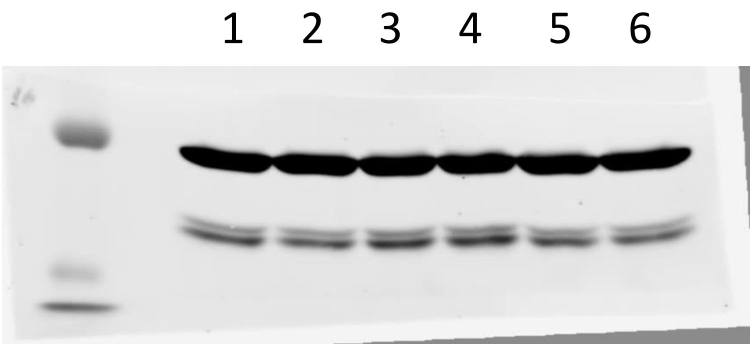

α-Tubulin, 52kD
Cell Signaling Catalog #3873

Full unedited gel for Figure S4B

1: NIH3T3: FGFR2-PHGDH treated with IgG1
2: NIH3T3: FGFR2-PHGDH treated with B/D
3: NIH3T3: FGFR2-PHGDH treated with B/C

1 2 3

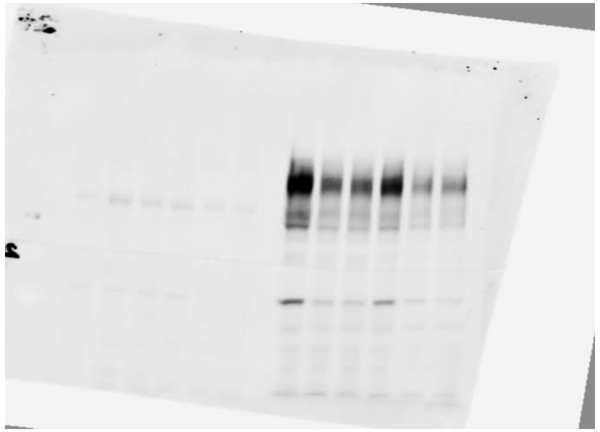

pFGFR (120, 145kD)
pFGFR (Tyr653/654)
Cell Signaling (Catalog#3471)

Full unedited gel for Figure S4B

1: NIH3T3: FGFR2-PHGDH treated with IgG1
2: NIH3T3: FGFR2-PHGDH treated with B/D
3: NIH3T3: FGFR2-PHGDH treated with B/C

1 2 3

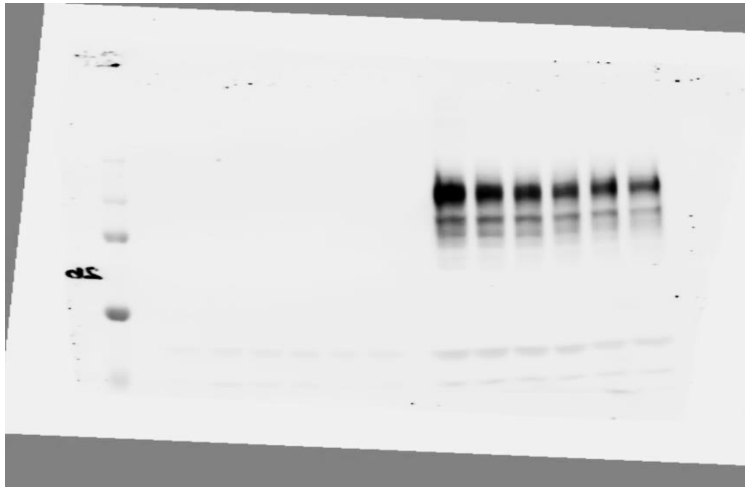

total FGFR2 (~130kD)
Antibody B/D

Full unedited gel for Figure S4B

1: NIH3T3: FGFR2-PHGDH treated with IgG1
2: NIH3T3: FGFR2-PHGDH treated with B/D
3: NIH3T3: FGFR2-PHGDH treated with B/C

1 2 3

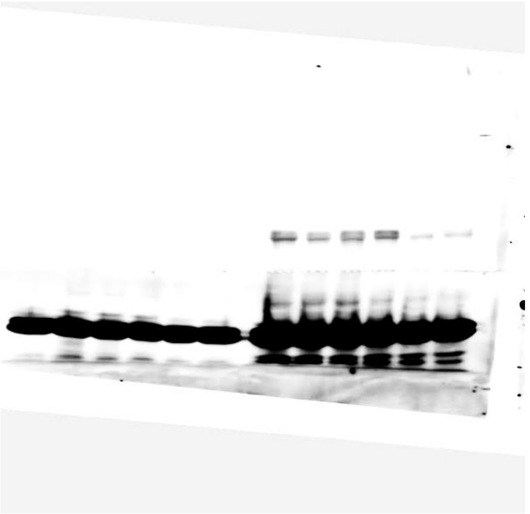

pFRS2 80-85kD
pFRS2(Y436)
Cell Signaling (Catalog#3861),

Full unedited gel for Figure S4B

1: NIH3T3: FGFR2-PHGDH treated with IgG1
2: NIH3T3: FGFR2-PHGDH treated with B/D
3: NIH3T3: FGFR2-PHGDH treated with B/C

1 2 3

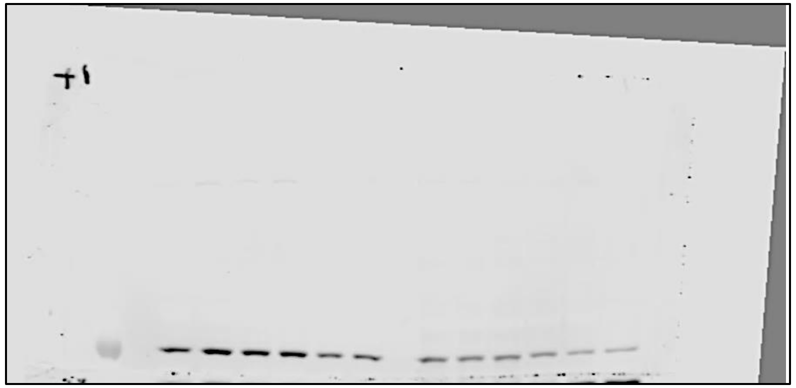

tFRS2 80-85kD
FRS2(Abcam, Catalog#ab183492)

Full unedited gel for Figure S4B

1: NIH3T3: FGFR2-PHGDH treated with IgG1
2: NIH3T3: FGFR2-PHGDH treated with B/D
3: NIH3T3: FGFR2-PHGDH treated with B/C

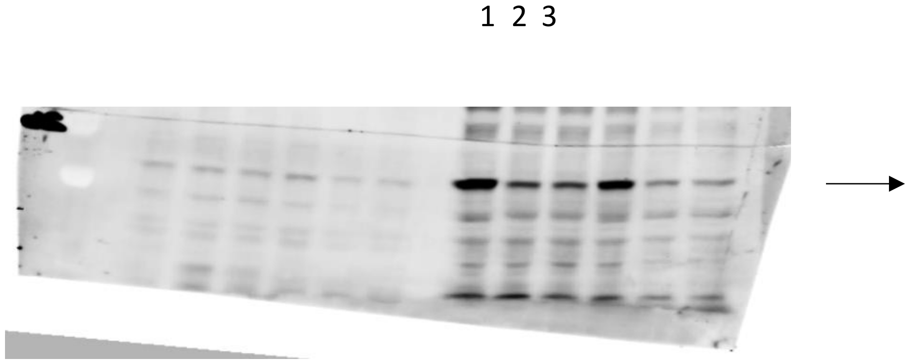

Phospho-Akt (Ser473), 60kD
Cell Signaling Catalog #4060

Full unedited gel for Figure S4B

1: NIH3T3: FGFR2-PHGDH treated with IgG1
2: NIH3T3: FGFR2-PHGDH treated with B/D
3: NIH3T3: FGFR2-PHGDH treated with B/C

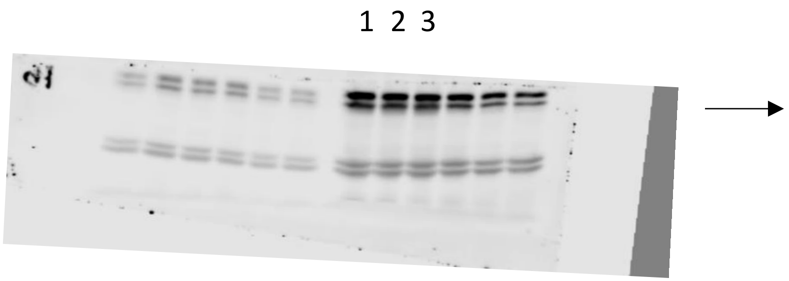

Akt (pan), 60kD
Cell Signaling Catalog #2920

Full unedited gel for Figure S4B

1: NIH3T3: FGFR2-PHGDH treated with IgG1
2: NIH3T3: FGFR2-PHGDH treated with B/D
3: NIH3T3: FGFR2-PHGDH treated with B/C

1 2 3

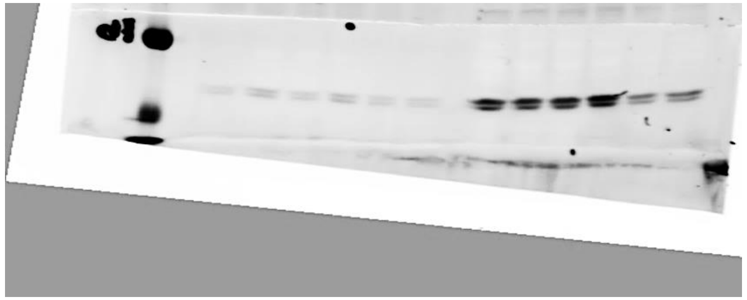

Phospho-p44/42 MAPK (Erk1/2) 45kD
(Thr202/Tyr204)
Cell Signaling Catalog#9106

Full unedited gel for Figure S4B

1: NIH3T3: FGFR2-PHGDH treated with IgG1
2: NIH3T3: FGFR2-PHGDH treated with B/D
3: NIH3T3: FGFR2-PHGDH treated with B/C

1 2 3

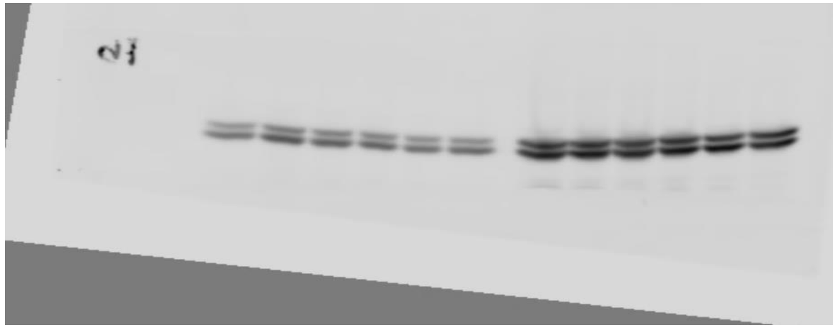

p44/42 MAPK (Erk1/2) 42, 44kD
Cell Signaling Catalog#4695

Full unedited gel for Figure S4B

1: NIH3T3: FGFR2-PHGDH treated with IgG1
2: NIH3T3: FGFR2-PHGDH treated with B/D
3: NIH3T3: FGFR2-PHGDH treated with B/C

1 2 3

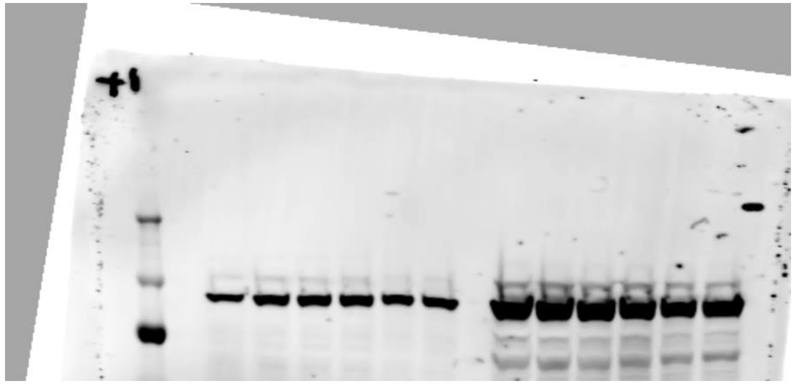

Vinculin 116kD
Abcam (Catalog#V9131)

Full unedited gel for Figure 5E

1: BaF3: FGFR2-PHGHD xenograft untreated Rep#1
2: BaF3: FGFR2-PHGHD xenograft untreated Rep#2
3: BaF3: FGFR2-PHGHD xenograft untreated Rep#3
4: BaF3: FGFR2-PHGHD xenograft treated with IgG1 15mg/kg Rep#1
5: BaF3: FGFR2-PHGHD xenograft treated with IgG1 15mg/kg Rep#2
6: BaF3: FGFR2-PHGHD xenograft treated with B/D 25mg/kg Rep#1
7: BaF3: FGFR2-PHGHD xenograft treated with B/D 25mg/kg Rep#2
8: BaF3: FGFR2-PHGHD xenograft treated with B/D 25mg/kg Rep#3
9: BaF3: FGFR2-PHGHD xenograft treated with B/C 25mg/kg Rep#1
10BaF3: FGFR2-PHGHD xenograft treated with B/C 25mg/kg Rep#2
11: BaF3: FGFR2-PHGHD xenograft treated with B/C 25mg/kg Rep#3

1 2 3 4 5 6 7 8 9 10 11

150kD

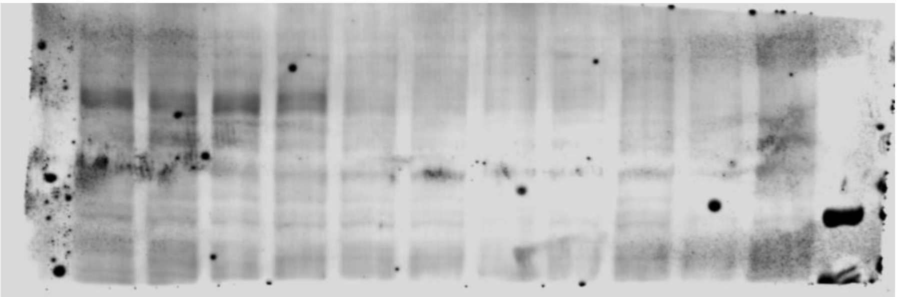

pFGFR (120, 145kD)
pFGFR (Tyr653/654)
Cell Signaling (Catalog#3471)

Full unedited gel for Figure 5E

1: BaF3: FGFR2-PHGHD xenograft untreated Rep#1
2: BaF3: FGFR2-PHGHD xenograft untreated Rep#2
3: BaF3: FGFR2-PHGHD xenograft untreated Rep#3
4: BaF3: FGFR2-PHGHD xenograft treated with IgG1 15mg/kg Rep#1
5: BaF3: FGFR2-PHGHD xenograft treated with IgG1 15mg/kg Rep#2
6: BaF3: FGFR2-PHGHD xenograft treated with B/D 25mg/kg Rep#1
7: BaF3: FGFR2-PHGHD xenograft treated with B/D 25mg/kg Rep#2
8: BaF3: FGFR2-PHGHD xenograft treated with B/D 25mg/kg Rep#3
9: BaF3: FGFR2-PHGHD xenograft treated with B/C 25mg/kg Rep#1
10BaF3: FGFR2-PHGHD xenograft treated with B/C 25mg/kg Rep#2
11: BaF3: FGFR2-PHGHD xenograft treated with B/C 25mg/kg Rep#3

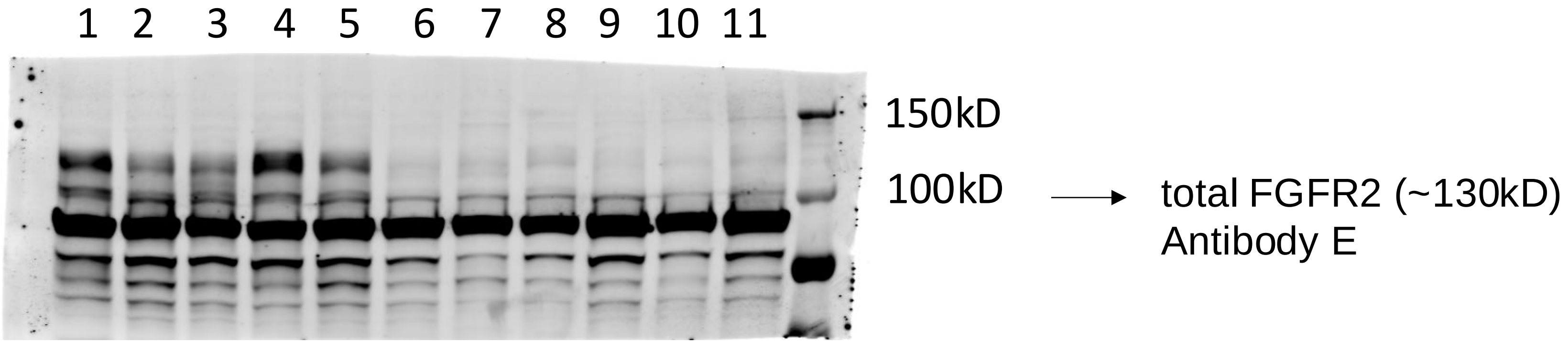

Full unedited gel for Figure 5E

1: BaF3: FGFR2-PHGHD xenograft untreated Rep#1
2: BaF3: FGFR2-PHGHD xenograft untreated Rep#2
3: BaF3: FGFR2-PHGHD xenograft untreated Rep#3
4: BaF3: FGFR2-PHGHD xenograft treated with IgG1 15mg/kg Rep#1
5: BaF3: FGFR2-PHGHD xenograft treated with IgG1 15mg/kg Rep#2
6: BaF3: FGFR2-PHGHD xenograft treated with B/D 25mg/kg Rep#1
7: BaF3: FGFR2-PHGHD xenograft treated with B/D 25mg/kg Rep#2
8: BaF3: FGFR2-PHGHD xenograft treated with B/D 25mg/kg Rep#3
9: BaF3: FGFR2-PHGHD xenograft treated with B/C 25mg/kg Rep#1
10BaF3: FGFR2-PHGHD xenograft treated with B/C 25mg/kg Rep#2
11: BaF3: FGFR2-PHGHD xenograft treated with B/C 25mg/kg Rep#3

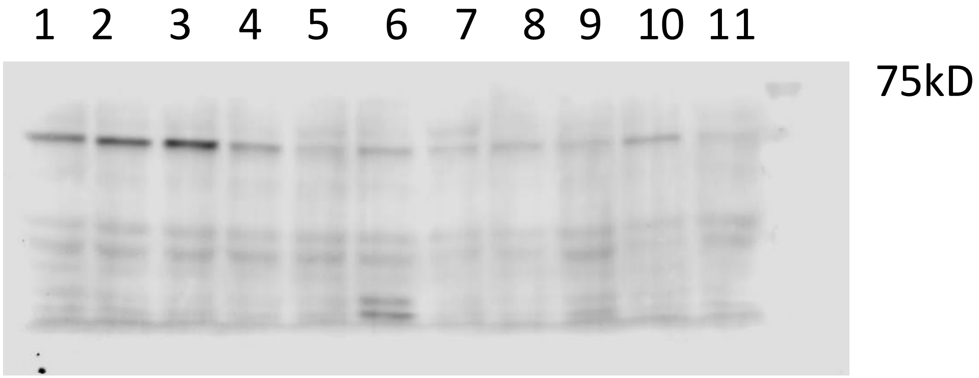

Phospho-Akt (Ser473), 60kD
Cell Signaling Catalog #4060

Full unedited gel for Figure 5E

1: BaF3: FGFR2-PHGHD xenograft untreated Rep#1
2: BaF3: FGFR2-PHGHD xenograft untreated Rep#2
3: BaF3: FGFR2-PHGHD xenograft untreated Rep#3
4: BaF3: FGFR2-PHGHD xenograft treated with IgG1 15mg/kg Rep#1
5: BaF3: FGFR2-PHGHD xenograft treated with IgG1 15mg/kg Rep#2
6: BaF3: FGFR2-PHGHD xenograft treated with B/D 25mg/kg Rep#1
7: BaF3: FGFR2-PHGHD xenograft treated with B/D 25mg/kg Rep#2
8: BaF3: FGFR2-PHGHD xenograft treated with B/D 25mg/kg Rep#3
9: BaF3: FGFR2-PHGHD xenograft treated with B/C 25mg/kg Rep#1
10BaF3: FGFR2-PHGHD xenograft treated with B/C 25mg/kg Rep#2
11: BaF3: FGFR2-PHGHD xenograft treated with B/C 25mg/kg Rep#3

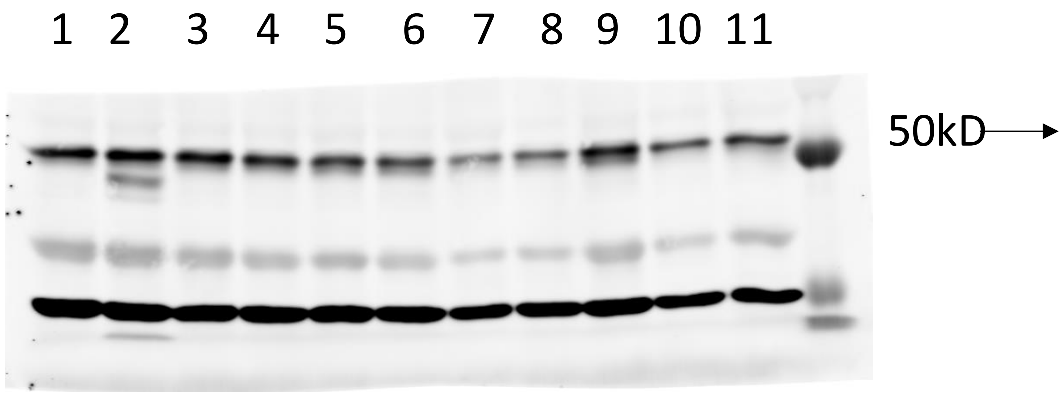

Akt (pan), 60kD
Cell Signaling Catalog #2920

Full unedited gel for Figure 5E

1: BaF3: FGFR2-PHGHD xenograft untreated Rep#1
2: BaF3: FGFR2-PHGHD xenograft untreated Rep#2
3: BaF3: FGFR2-PHGHD xenograft untreated Rep#3
4: BaF3: FGFR2-PHGHD xenograft treated with IgG1 15mg/kg Rep#1
5: BaF3: FGFR2-PHGHD xenograft treated with IgG1 15mg/kg Rep#2
6: BaF3: FGFR2-PHGHD xenograft treated with B/D 25mg/kg Rep#1
7: BaF3: FGFR2-PHGHD xenograft treated with B/D 25mg/kg Rep#2
8: BaF3: FGFR2-PHGHD xenograft treated with B/D 25mg/kg Rep#3
9: BaF3: FGFR2-PHGHD xenograft treated with B/C 25mg/kg Rep#1
10BaF3: FGFR2-PHGHD xenograft treated with B/C 25mg/kg Rep#2
11: BaF3: FGFR2-PHGHD xenograft treated with B/C 25mg/kg Rep#3

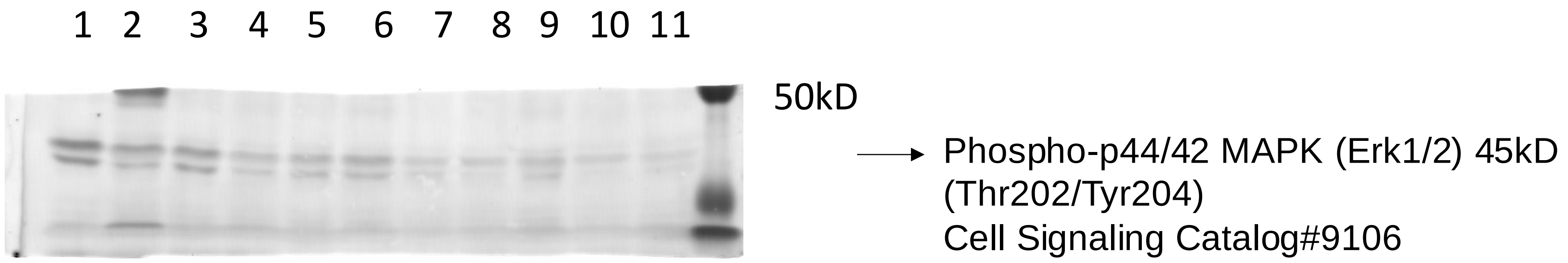

Full unedited gel for Figure 5E

1: BaF3: FGFR2-PHGHD xenograft untreated Rep#1
2: BaF3: FGFR2-PHGHD xenograft untreated Rep#2
3: BaF3: FGFR2-PHGHD xenograft untreated Rep#3
4: BaF3: FGFR2-PHGHD xenograft treated with IgG1 15mg/kg Rep#1
5: BaF3: FGFR2-PHGHD xenograft treated with IgG1 15mg/kg Rep#2
6: BaF3: FGFR2-PHGHD xenograft treated with B/D 25mg/kg Rep#1
7: BaF3: FGFR2-PHGHD xenograft treated with B/D 25mg/kg Rep#2
8: BaF3: FGFR2-PHGHD xenograft treated with B/D 25mg/kg Rep#3
9: BaF3: FGFR2-PHGHD xenograft treated with B/C 25mg/kg Rep#1
10BaF3: FGFR2-PHGHD xenograft treated with B/C 25mg/kg Rep#2
11: BaF3: FGFR2-PHGHD xenograft treated with B/C 25mg/kg Rep#3

1 2 3 4 5 6 7 8 9 10 11

50kD

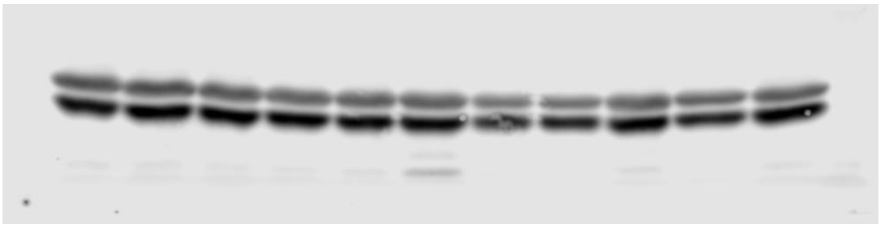

p44/42 MAPK (Erk1/2) 42, 44kD
Cell Signaling Catalog#4695

Full unedited gel for Figure 5E

1: BaF3: FGFR2-PHGHD xenograft untreated Rep#1
2: BaF3: FGFR2-PHGHD xenograft untreated Rep#2
3: BaF3: FGFR2-PHGHD xenograft untreated Rep#3
4: BaF3: FGFR2-PHGHD xenograft treated with IgG1 15mg/kg Rep#1
5: BaF3: FGFR2-PHGHD xenograft treated with IgG1 15mg/kg Rep#2
6: BaF3: FGFR2-PHGHD xenograft treated with B/D 25mg/kg Rep#1
7: BaF3: FGFR2-PHGHD xenograft treated with B/D 25mg/kg Rep#2
8: BaF3: FGFR2-PHGHD xenograft treated with B/D 25mg/kg Rep#3
9: BaF3: FGFR2-PHGHD xenograft treated with B/C 25mg/kg Rep#1
10BaF3: FGFR2-PHGHD xenograft treated with B/C 25mg/kg Rep#2
11: BaF3: FGFR2-PHGHD xenograft treated with B/C 25mg/kg Rep#3

1 2 3 4 5 6 7 8 9 10 11

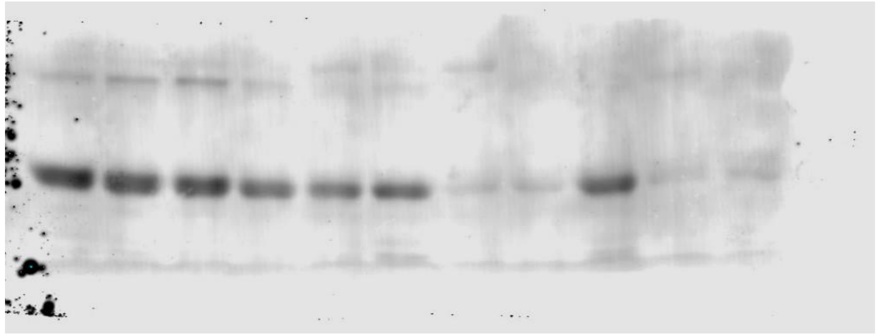

Phospho-MEK1/2 (Ser217/221) 45kD
Cell Signaling Catalog #9154

Full unedited gel for Figure 5E

1: BaF3: FGFR2-PHGHD xenograft untreated Rep#1
2: BaF3: FGFR2-PHGHD xenograft untreated Rep#2
3: BaF3: FGFR2-PHGHD xenograft untreated Rep#3
4: BaF3: FGFR2-PHGHD xenograft treated with IgG1 15mg/kg Rep#1
5: BaF3: FGFR2-PHGHD xenograft treated with IgG1 15mg/kg Rep#2
6: BaF3: FGFR2-PHGHD xenograft treated with B/D 25mg/kg Rep#1
7: BaF3: FGFR2-PHGHD xenograft treated with B/D 25mg/kg Rep#2
8: BaF3: FGFR2-PHGHD xenograft treated with B/D 25mg/kg Rep#3
9: BaF3: FGFR2-PHGHD xenograft treated with B/C 25mg/kg Rep#1
10BaF3: FGFR2-PHGHD xenograft treated with B/C 25mg/kg Rep#2
11: BaF3: FGFR2-PHGHD xenograft treated with B/C 25mg/kg Rep#3

1 2 3 4 5 6 7 8 9 10 11

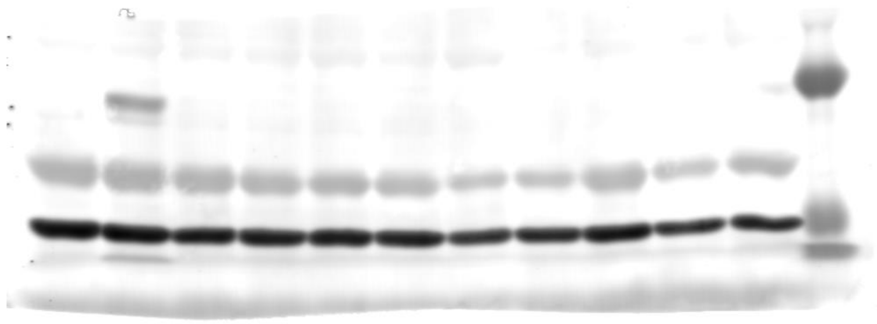

MEK1/2 (L38C12), 45kD
Cell Signaling Catalog #4694

Full unedited gel for Figure 5E

1: BaF3: FGFR2-PHGHD xenograft untreated Rep#1
2: BaF3: FGFR2-PHGHD xenograft untreated Rep#2
3: BaF3: FGFR2-PHGHD xenograft untreated Rep#3
4: BaF3: FGFR2-PHGHD xenograft treated with IgG1 15mg/kg Rep#1
5: BaF3: FGFR2-PHGHD xenograft treated with IgG1 15mg/kg Rep#2
6: BaF3: FGFR2-PHGHD xenograft treated with B/D 25mg/kg Rep#1
7: BaF3: FGFR2-PHGHD xenograft treated with B/D 25mg/kg Rep#2
8: BaF3: FGFR2-PHGHD xenograft treated with B/D 25mg/kg Rep#3
9: BaF3: FGFR2-PHGHD xenograft treated with B/C 25mg/kg Rep#1
10BaF3: FGFR2-PHGHD xenograft treated with B/C 25mg/kg Rep#2
11: BaF3: FGFR2-PHGHD xenograft treated with B/C 25mg/kg Rep#3

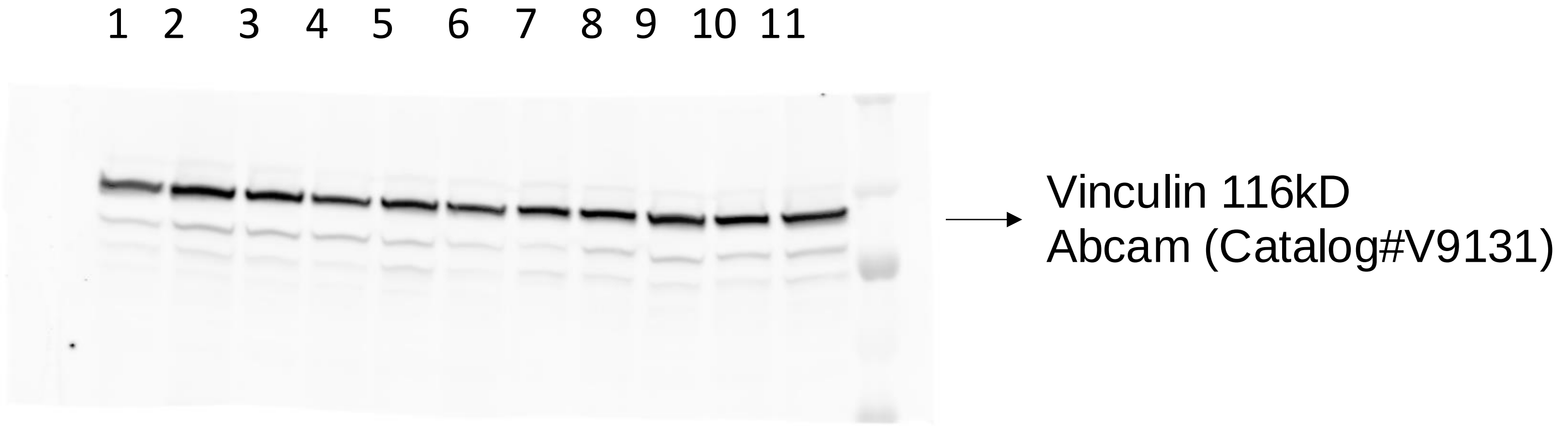

Full unedited gel for Figure 5F

1: ICC13-7 xenograft treated with IgG1 control Rep#1
2: ICC13-7 xenograft treated with IgG1 control Rep#2
3: ICC13-7 xenograft treated with IgG1 control Rep#3
4: ICC13-7 xenograft treated with B/D 10mg/kg Rep#1
5: ICC13-7 xenograft treated with B/D 10mg/kg Rep#2
6: ICC13-7 xenograft treated with B/D 30mg/kg Rep#1
7: ICC13-7 xenograft treated with B/D 30mg/kg Rep#2
8: ICC13-7 xenograft treated with B/D 30mg/kg Rep#3
9: ICC13-7 xenograft treated with B/C 10mg/kg Rep#1
10: ICC13-7 xenograft treated with B/C 10mg/kg Rep#2
11: ICC13-7 xenograft treated with B/C 30mg/kg Rep#1
12: ICC13-7 xenograft treated with B/C 30mg/kg Rep#2
13: ICC13-7 xenograft treated with B/C 30mg/kg Rep#3

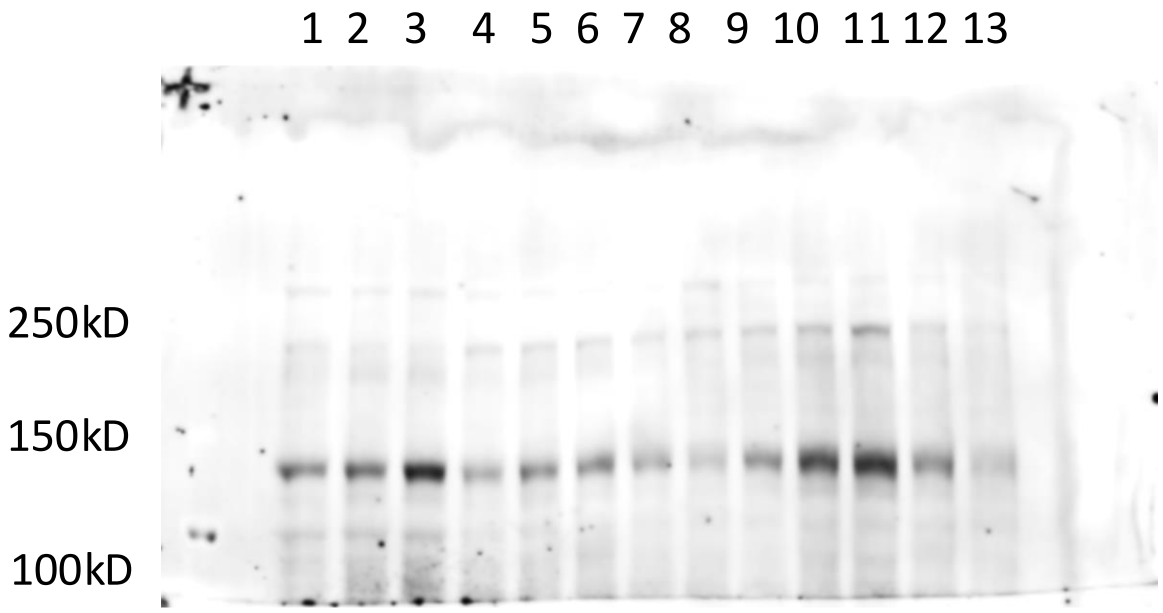

pFGFR (120, 145kD)
pFGFR (Tyr653/654)
Cell Signaling (Catalog#3471)

Full unedited gel for Figure 5F

1: ICC13-7 xenograft treated with IgG1 control Rep#1
2: ICC13-7 xenograft treated with IgG1 control Rep#2
3: ICC13-7 xenograft treated with IgG1 control Rep#3
4: ICC13-7 xenograft treated with B/D 10mg/kg Rep#1
5: ICC13-7 xenograft treated with B/D 10mg/kg Rep#2
6: ICC13-7 xenograft treated with B/D 30mg/kg Rep#1
7: ICC13-7 xenograft treated with B/D 30mg/kg Rep#2
8: ICC13-7 xenograft treated with B/D 30mg/kg Rep#3
9: ICC13-7 xenograft treated with B/C 10mg/kg Rep#1
10: ICC13-7 xenograft treated with B/C 10mg/kg Rep#2
11: ICC13-7 xenograft treated with B/C 30mg/kg Rep#1
12: ICC13-7 xenograft treated with B/C 30mg/kg Rep#2
13: ICC13-7 xenograft treated with B/C 30mg/kg Rep#3

1 2 3 4 5 6 7 8 9 10 11 12 13

250kD
150kD
100kD

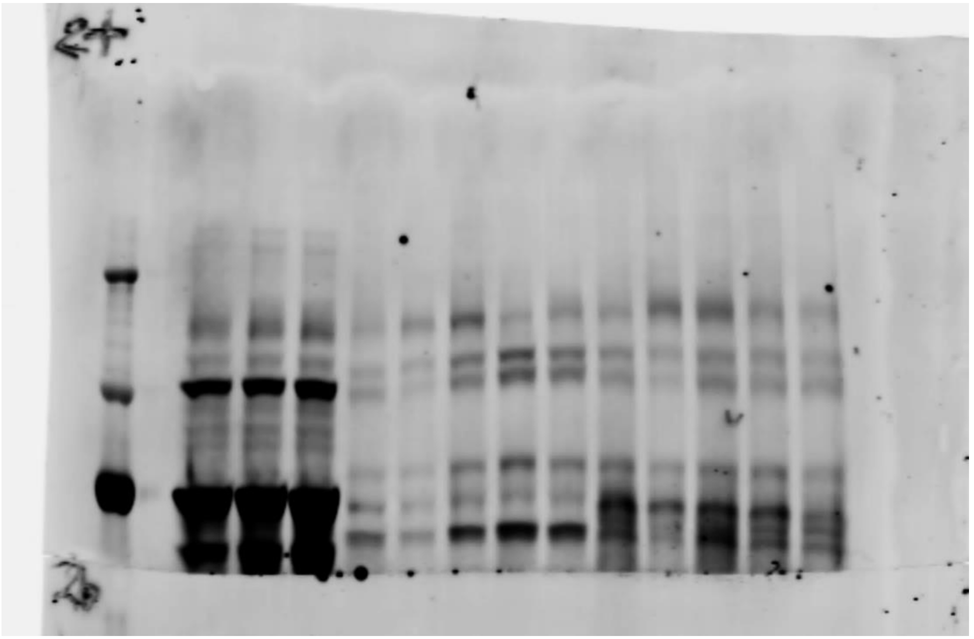

total FGFR2 (~130kD)
Antibody B/D

Full unedited gel for Figure 5F

1: ICC13-7 xenograft treated with IgG1 control Rep#1
2: ICC13-7 xenograft treated with IgG1 control Rep#2
3: ICC13-7 xenograft treated with IgG1 control Rep#3
4: ICC13-7 xenograft treated with B/D 10mg/kg Rep#1
5: ICC13-7 xenograft treated with B/D 10mg/kg Rep#2
6: ICC13-7 xenograft treated with B/D 30mg/kg Rep#1
7: ICC13-7 xenograft treated with B/D 30mg/kg Rep#2
8: ICC13-7 xenograft treated with B/D 30mg/kg Rep#3
9: ICC13-7 xenograft treated with B/C 10mg/kg Rep#1
10: ICC13-7 xenograft treated with B/C 10mg/kg Rep#2
11: ICC13-7 xenograft treated with B/C 30mg/kg Rep#1
12: ICC13-7 xenograft treated with B/C 30mg/kg Rep#2
13: ICC13-7 xenograft treated with B/C 30mg/kg Rep#3

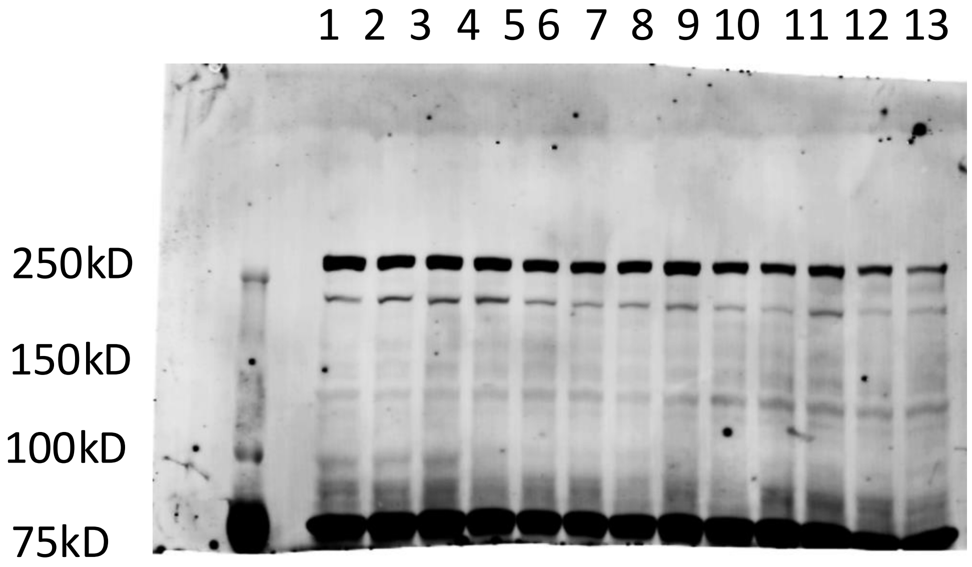

pFRS2 80-85kD
pFRS2(Y436)
Cell Signaling (Catalog#3861),

Full unedited gel for Figure 5F

1: ICC13-7 xenograft treated with IgG1 control Rep#1
2: ICC13-7 xenograft treated with IgG1 control Rep#2
3: ICC13-7 xenograft treated with IgG1 control Rep#3
4: ICC13-7 xenograft treated with B/D 10mg/kg Rep#1
5: ICC13-7 xenograft treated with B/D 10mg/kg Rep#2
6: ICC13-7 xenograft treated with B/D 30mg/kg Rep#1
7: ICC13-7 xenograft treated with B/D 30mg/kg Rep#2
8: ICC13-7 xenograft treated with B/D 30mg/kg Rep#3
9: ICC13-7 xenograft treated with B/C 10mg/kg Rep#1
10: ICC13-7 xenograft treated with B/C 10mg/kg Rep#2
11: ICC13-7 xenograft treated with B/C 30mg/kg Rep#1
12: ICC13-7 xenograft treated with B/C 30mg/kg Rep#2
13: ICC13-7 xenograft treated with B/C 30mg/kg Rep#3

1 2 3 4 5 6 7 8 9 10 11 12 13

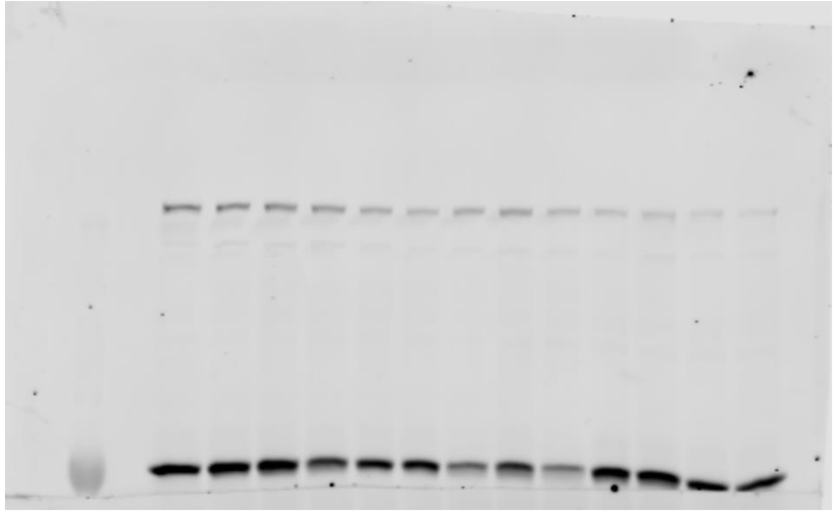

75kD

tFRS2 80-85kD
FRS2(Abcam, Catalog#ab183492)

Full unedited gel for Figure 5F

1: ICC13-7 xenograft treated with IgG1 control Rep#1
2: ICC13-7 xenograft treated with IgG1 control Rep#2
3: ICC13-7 xenograft treated with IgG1 control Rep#3
4: ICC13-7 xenograft treated with B/D 10mg/kg Rep#1
5: ICC13-7 xenograft treated with B/D 10mg/kg Rep#2
6: ICC13-7 xenograft treated with B/D 30mg/kg Rep#1
7: ICC13-7 xenograft treated with B/D 30mg/kg Rep#2
8: ICC13-7 xenograft treated with B/D 30mg/kg Rep#3
9: ICC13-7 xenograft treated with B/C 10mg/kg Rep#1
10: ICC13-7 xenograft treated with B/C 10mg/kg Rep#2
11: ICC13-7 xenograft treated with B/C 30mg/kg Rep#1
12: ICC13-7 xenograft treated with B/C 30mg/kg Rep#2
13: ICC13-7 xenograft treated with B/C 30mg/kg Rep#3

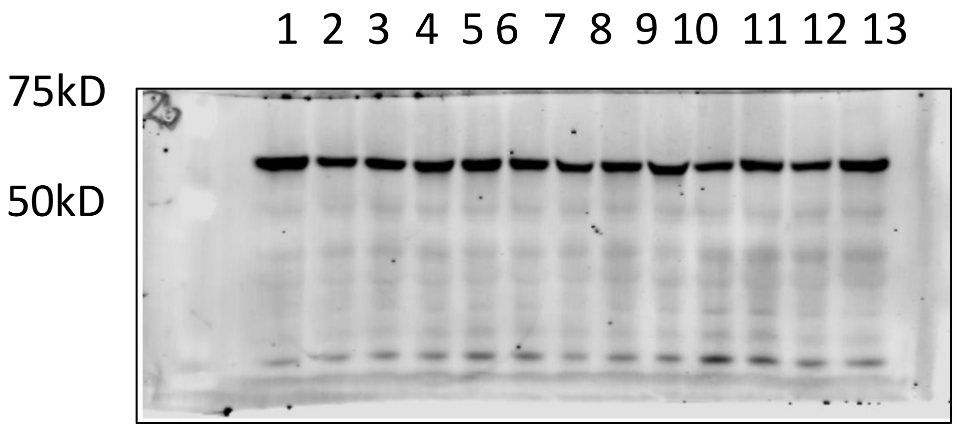

Phospho-Akt (Ser473), 60kD
Cell Signaling Catalog #4060

Full unedited gel for Figure 5F

1: ICC13-7 xenograft treated with IgG1 control Rep#1
2: ICC13-7 xenograft treated with IgG1 control Rep#2
3: ICC13-7 xenograft treated with IgG1 control Rep#3
4: ICC13-7 xenograft treated with B/D 10mg/kg Rep#1
5: ICC13-7 xenograft treated with B/D 10mg/kg Rep#2
6: ICC13-7 xenograft treated with B/D 30mg/kg Rep#1
7: ICC13-7 xenograft treated with B/D 30mg/kg Rep#2
8: ICC13-7 xenograft treated with B/D 30mg/kg Rep#3
9: ICC13-7 xenograft treated with B/C 10mg/kg Rep#1
10: ICC13-7 xenograft treated with B/C 10mg/kg Rep#2
11: ICC13-7 xenograft treated with B/C 30mg/kg Rep#1
12: ICC13-7 xenograft treated with B/C 30mg/kg Rep#2
13: ICC13-7 xenograft treated with B/C 30mg/kg Rep#3

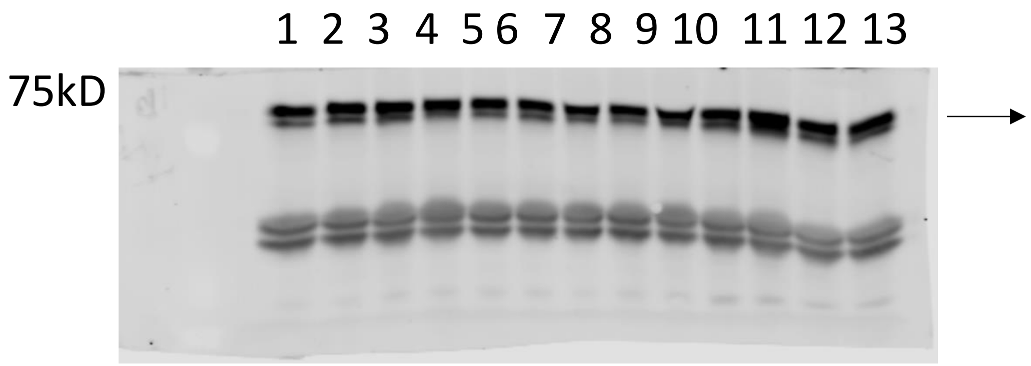

Akt (pan), 60kD
Cell Signaling Catalog #2920

Full unedited gel for Figure 5F

1: ICC13-7 xenograft treated with IgG1 control Rep#1
2: ICC13-7 xenograft treated with IgG1 control Rep#2
3: ICC13-7 xenograft treated with IgG1 control Rep#3
4: ICC13-7 xenograft treated with B/D 10mg/kg Rep#1
5: ICC13-7 xenograft treated with B/D 10mg/kg Rep#2
6: ICC13-7 xenograft treated with B/D 30mg/kg Rep#1
7: ICC13-7 xenograft treated with B/D 30mg/kg Rep#2
8: ICC13-7 xenograft treated with B/D 30mg/kg Rep#3
9: ICC13-7 xenograft treated with B/C 10mg/kg Rep#1
10: ICC13-7 xenograft treated with B/C 10mg/kg Rep#2
11: ICC13-7 xenograft treated with B/C 30mg/kg Rep#1
12: ICC13-7 xenograft treated with B/C 30mg/kg Rep#2
13: ICC13-7 xenograft treated with B/C 30mg/kg Rep#3

1 2 3 4 5 6 7 8 9 10 11 12 13

50kD

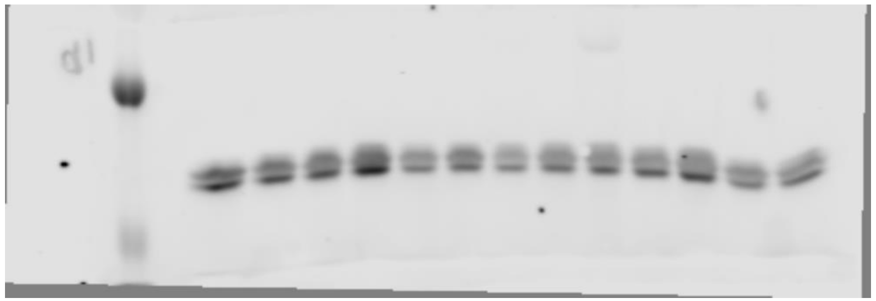

Phospho-p44/42 MAPK (Erk1/2) 45kD (Thr202/Tyr204)
Cell Signaling Catalog#9106

Full unedited gel for Figure 5F

1: ICC13-7 xenograft treated with IgG1 control Rep#1
2: ICC13-7 xenograft treated with IgG1 control Rep#2
3: ICC13-7 xenograft treated with IgG1 control Rep#3
4: ICC13-7 xenograft treated with B/D 10mg/kg Rep#1
5: ICC13-7 xenograft treated with B/D 10mg/kg Rep#2
6: ICC13-7 xenograft treated with B/D 30mg/kg Rep#1
7: ICC13-7 xenograft treated with B/D 30mg/kg Rep#2
8: ICC13-7 xenograft treated with B/D 30mg/kg Rep#3
9: ICC13-7 xenograft treated with B/C 10mg/kg Rep#1
10: ICC13-7 xenograft treated with B/C 10mg/kg Rep#2
11: ICC13-7 xenograft treated with B/C 30mg/kg Rep#1
12: ICC13-7 xenograft treated with B/C 30mg/kg Rep#2
13: ICC13-7 xenograft treated with B/C 30mg/kg Rep#3

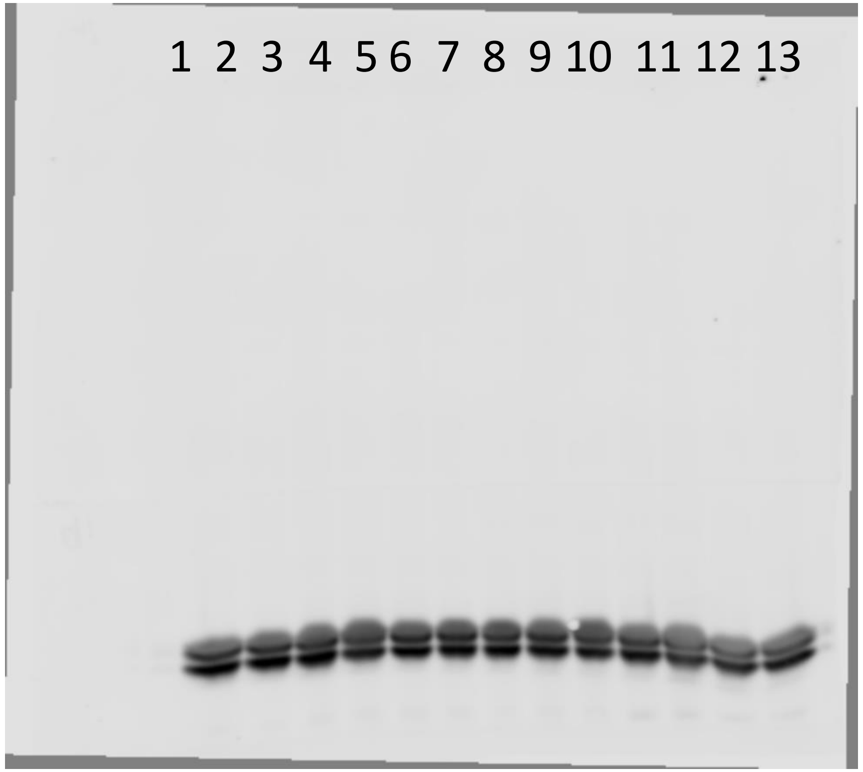

p44/42 MAPK (Erk1/2) 42, 44kD
Cell Signaling Catalog#4695

Full unedited gel for Figure 5F

1: ICC13-7 xenograft treated with IgG1 control Rep#1
2: ICC13-7 xenograft treated with IgG1 control Rep#2
3: ICC13-7 xenograft treated with IgG1 control Rep#3
4: ICC13-7 xenograft treated with B/D 10mg/kg Rep#1
5: ICC13-7 xenograft treated with B/D 10mg/kg Rep#2
6: ICC13-7 xenograft treated with B/D 30mg/kg Rep#1
7: ICC13-7 xenograft treated with B/D 30mg/kg Rep#2
8: ICC13-7 xenograft treated with B/D 30mg/kg Rep#3
9: ICC13-7 xenograft treated with B/C 10mg/kg Rep#1
10: ICC13-7 xenograft treated with B/C 10mg/kg Rep#2
11: ICC13-7 xenograft treated with B/C 30mg/kg Rep#1
12: ICC13-7 xenograft treated with B/C 30mg/kg Rep#2
13: ICC13-7 xenograft treated with B/C 30mg/kg Rep#3

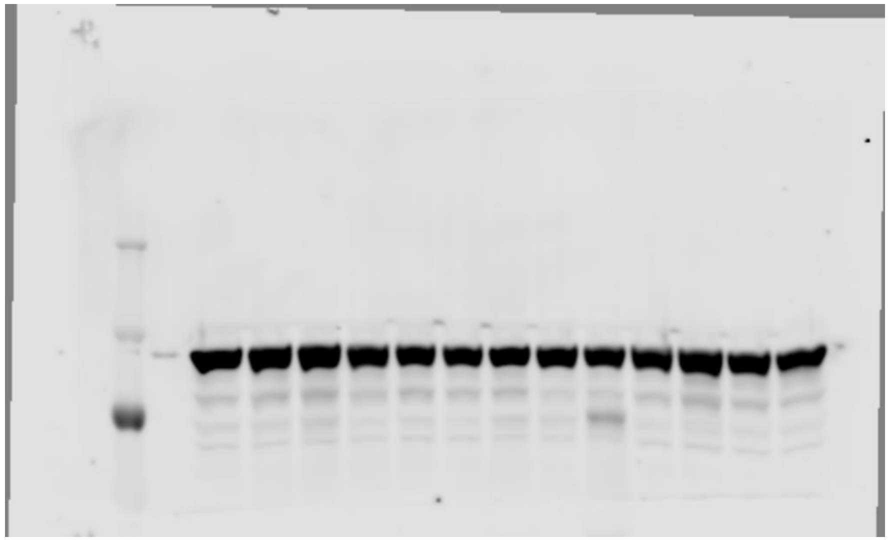

Full unedited gel for Figure S5E

1: ICC13-7 xenograft treated with IgG1 control Rep#1
2: ICC13-7 xenograft treated with IgG1 control Rep#2
3: ICC13-7 xenograft treated with IgG1 control Rep#3
4: ICC13-7 xenograft treated with B 10mg/kg Rep#1
5: ICC13-7 xenograft treated with B 10mg/kg Rep#2
6: ICC13-7 xenograft treated with B 10mg/kg Rep#3
7: ICC13-7 xenograft treated with D 10mg/kg Rep#1
8: ICC13-7 xenograft treated with D 10mg/kg Rep#2
9: ICC13-7 xenograft treated with D 10mg/kg Rep#3
10: ICC13-7 xenograft treated with C 10mg/kg Rep#1
11: ICC13-7 xenograft treated with C 10mg/kg Rep#2
12: ICC13-7 xenograft treated with C 10mg/kg Rep#3

L 1 2 3 4 5 6 7 8 9 10 11 12

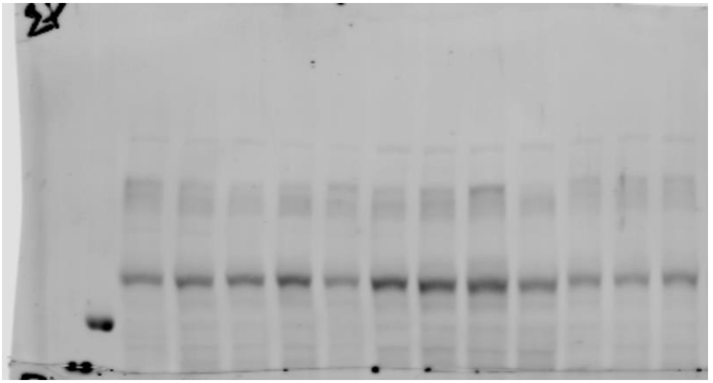

100kD

pFGFR (120, 145kD)
pFGFR (Tyr653/654)
Cell Signaling (Catalog#3471)

Full unedited gel for Figure S5E

1: ICC13-7 xenograft treated with IgG1 control Rep#1
2: ICC13-7 xenograft treated with IgG1 control Rep#2
3: ICC13-7 xenograft treated with IgG1 control Rep#3
4: ICC13-7 xenograft treated with B 10mg/kg Rep#1
5: ICC13-7 xenograft treated with B 10mg/kg Rep#2
6: ICC13-7 xenograft treated with B 10mg/kg Rep#3
7: ICC13-7 xenograft treated with D 10mg/kg Rep#1
8: ICC13-7 xenograft treated with D 10mg/kg Rep#2
9: ICC13-7 xenograft treated with D 10mg/kg Rep#3
10: ICC13-7 xenograft treated with C 10mg/kg Rep#1
11: ICC13-7 xenograft treated with C 10mg/kg Rep#2
12: ICC13-7 xenograft treated with C 10mg/kg Rep#3

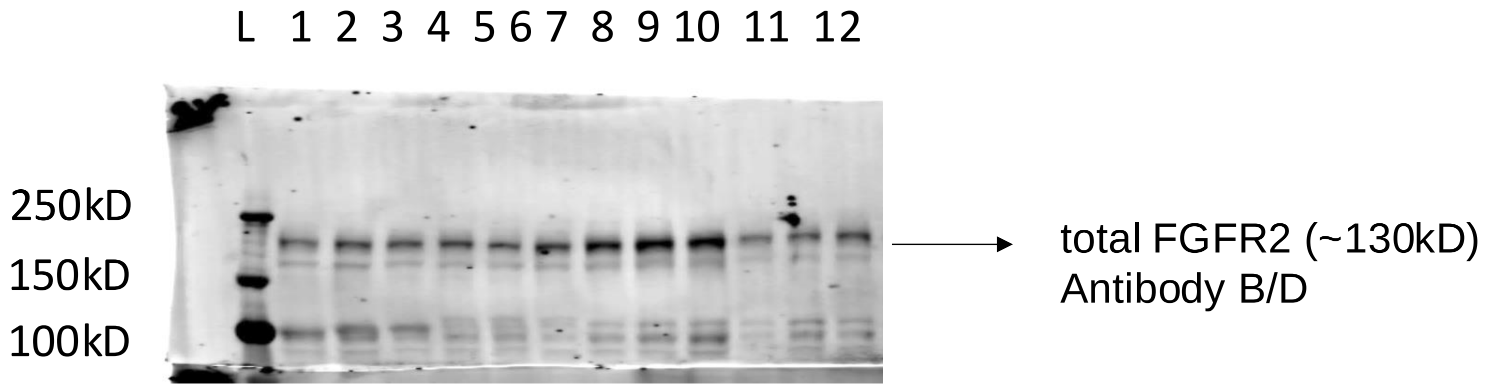

Full unedited gel for Figure S5E

1: ICC13-7 xenograft treated with IgG1 control Rep#1
2: ICC13-7 xenograft treated with IgG1 control Rep#2
3: ICC13-7 xenograft treated with IgG1 control Rep#3
4: ICC13-7 xenograft treated with B 10mg/kg Rep#1
5: ICC13-7 xenograft treated with B 10mg/kg Rep#2
6: ICC13-7 xenograft treated with B 10mg/kg Rep#3
7: ICC13-7 xenograft treated with D 10mg/kg Rep#1
8: ICC13-7 xenograft treated with D 10mg/kg Rep#2
9: ICC13-7 xenograft treated with D 10mg/kg Rep#3
10: ICC13-7 xenograft treated with C 10mg/kg Rep#1
11: ICC13-7 xenograft treated with C 10mg/kg Rep#2
12: ICC13-7 xenograft treated with C 10mg/kg Rep#3

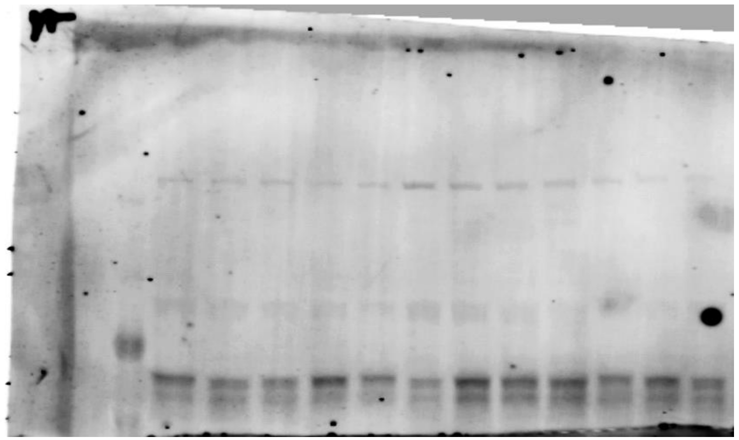

Full unedited gel for Figure S5E

1: ICC13-7 xenograft treated with IgG1 control Rep#1
2: ICC13-7 xenograft treated with IgG1 control Rep#2
3: ICC13-7 xenograft treated with IgG1 control Rep#3
4: ICC13-7 xenograft treated with B 10mg/kg Rep#1
5: ICC13-7 xenograft treated with B 10mg/kg Rep#2
6: ICC13-7 xenograft treated with B 10mg/kg Rep#3
7: ICC13-7 xenograft treated with D 10mg/kg Rep#1
8: ICC13-7 xenograft treated with D 10mg/kg Rep#2
9: ICC13-7 xenograft treated with D 10mg/kg Rep#3
10: ICC13-7 xenograft treated with C 10mg/kg Rep#1
11: ICC13-7 xenograft treated with C 10mg/kg Rep#2
12: ICC13-7 xenograft treated with C 10mg/kg Rep#3

L 1 2 3 4 5 6 7 8 9 10 11 12

100kD

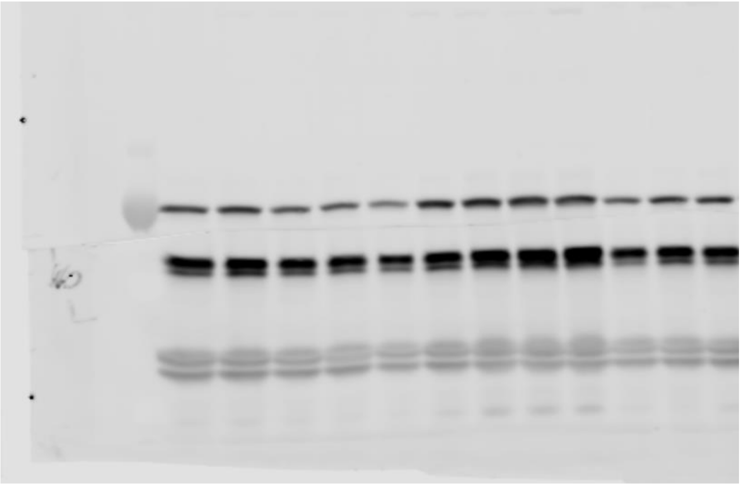

tFRS2 80-85kD
FRS2(Abcam, Catalog#ab183492)

Full unedited gel for Figure S5E

1: ICC13-7 xenograft treated with IgG1 control Rep#1
2: ICC13-7 xenograft treated with IgG1 control Rep#2
3: ICC13-7 xenograft treated with IgG1 control Rep#3
4: ICC13-7 xenograft treated with B 10mg/kg Rep#1
5: ICC13-7 xenograft treated with B 10mg/kg Rep#2
6: ICC13-7 xenograft treated with B 10mg/kg Rep#3
7: ICC13-7 xenograft treated with D 10mg/kg Rep#1
8: ICC13-7 xenograft treated with D 10mg/kg Rep#2
9: ICC13-7 xenograft treated with D 10mg/kg Rep#3
10: ICC13-7 xenograft treated with C 10mg/kg Rep#1
11: ICC13-7 xenograft treated with C 10mg/kg Rep#2
12: ICC13-7 xenograft treated with C 10mg/kg Rep#3

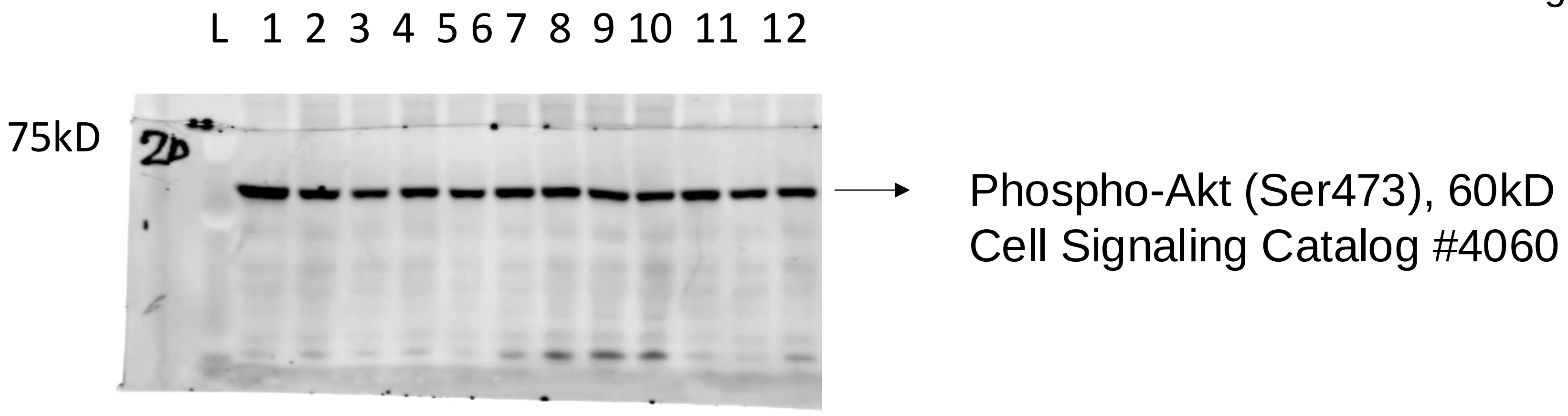

Full unedited gel for Figure S5E

1: ICC13-7 xenograft treated with IgG1 control Rep#1
2: ICC13-7 xenograft treated with IgG1 control Rep#2
3: ICC13-7 xenograft treated with IgG1 control Rep#3
4: ICC13-7 xenograft treated with B 10mg/kg Rep#1
5: ICC13-7 xenograft treated with B 10mg/kg Rep#2
6: ICC13-7 xenograft treated with B 10mg/kg Rep#3
7: ICC13-7 xenograft treated with D 10mg/kg Rep#1
8: ICC13-7 xenograft treated with D 10mg/kg Rep#2
9: ICC13-7 xenograft treated with D 10mg/kg Rep#3
10: ICC13-7 xenograft treated with C 10mg/kg Rep#1
11: ICC13-7 xenograft treated with C 10mg/kg Rep#2
12: ICC13-7 xenograft treated with C 10mg/kg Rep#3

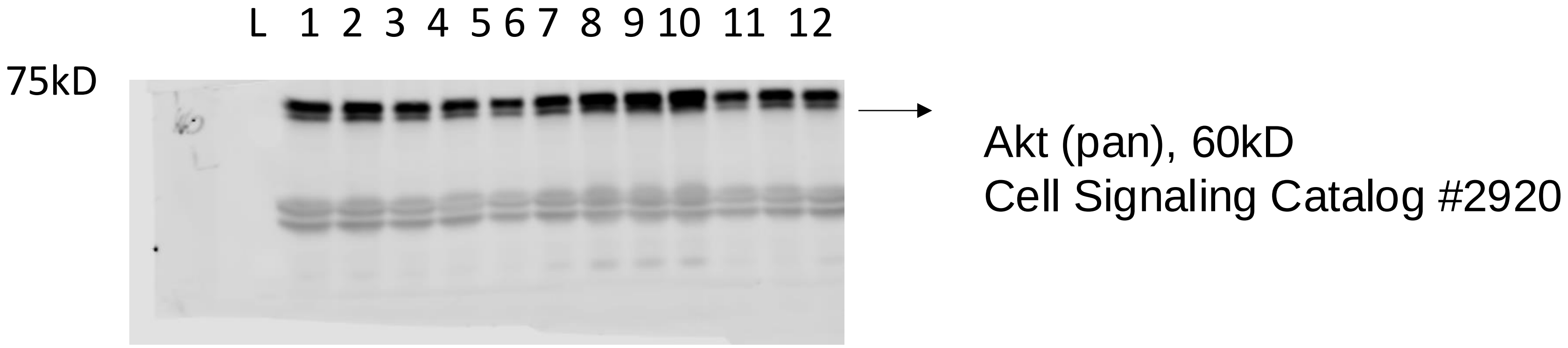

Full unedited gel for Figure S5E

1: ICC13-7 xenograft treated with IgG1 control Rep#1
2: ICC13-7 xenograft treated with IgG1 control Rep#2
3: ICC13-7 xenograft treated with IgG1 control Rep#3
4: ICC13-7 xenograft treated with B 10mg/kg Rep#1
5: ICC13-7 xenograft treated with B 10mg/kg Rep#2
6: ICC13-7 xenograft treated with B 10mg/kg Rep#3
7: ICC13-7 xenograft treated with D 10mg/kg Rep#1
8: ICC13-7 xenograft treated with D 10mg/kg Rep#2
9: ICC13-7 xenograft treated with D 10mg/kg Rep#3
10: ICC13-7 xenograft treated with C 10mg/kg Rep#1
11: ICC13-7 xenograft treated with C 10mg/kg Rep#2
12: ICC13-7 xenograft treated with C 10mg/kg Rep#3

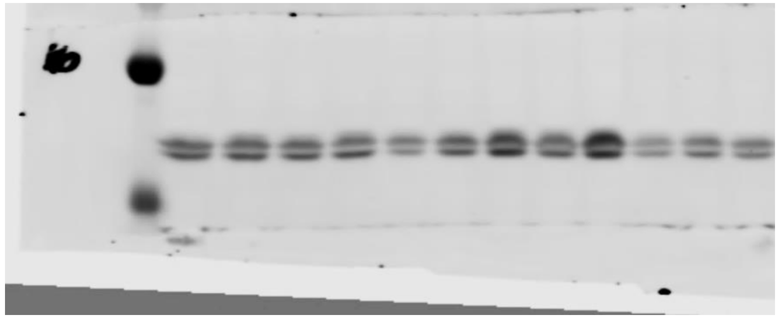

Phospho-p44/42 MAPK (Erk1/2) 45kD
(Thr202/Tyr204)
Cell Signaling Catalog#9106

Full unedited gel for Figure S5E

1: ICC13-7 xenograft treated with IgG1 control Rep#1
2: ICC13-7 xenograft treated with IgG1 control Rep#2
3: ICC13-7 xenograft treated with IgG1 control Rep#3
4: ICC13-7 xenograft treated with B 10mg/kg Rep#1
5: ICC13-7 xenograft treated with B 10mg/kg Rep#2
6: ICC13-7 xenograft treated with B 10mg/kg Rep#3
7: ICC13-7 xenograft treated with D 10mg/kg Rep#1
8: ICC13-7 xenograft treated with D 10mg/kg Rep#2
9: ICC13-7 xenograft treated with D 10mg/kg Rep#3
10: ICC13-7 xenograft treated with C 10mg/kg Rep#1
11: ICC13-7 xenograft treated with C 10mg/kg Rep#2
12: ICC13-7 xenograft treated with C 10mg/kg Rep#3

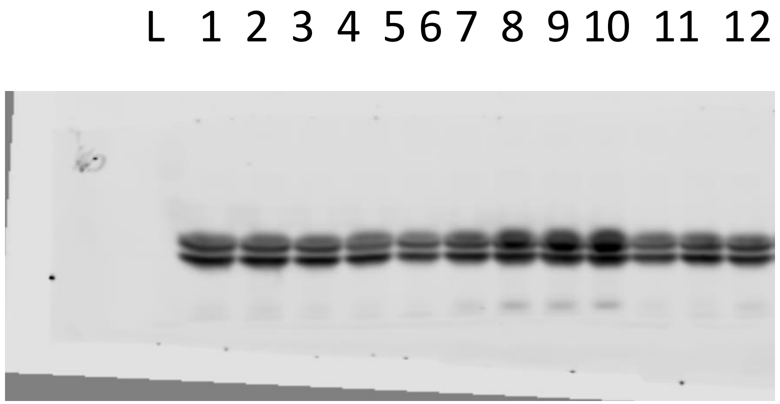

p44/42 MAPK (Erk1/2) 42, 44kD
Cell Signaling Catalog#4695

Full unedited gel for Figure S5E

1: ICC13-7 xenograft treated with IgG1 control Rep#1
2: ICC13-7 xenograft treated with IgG1 control Rep#2
3: ICC13-7 xenograft treated with IgG1 control Rep#3
4: ICC13-7 xenograft treated with B 10mg/kg Rep#1
5: ICC13-7 xenograft treated with B 10mg/kg Rep#2
6: ICC13-7 xenograft treated with B 10mg/kg Rep#3
7: ICC13-7 xenograft treated with D 10mg/kg Rep#1
8: ICC13-7 xenograft treated with D 10mg/kg Rep#2
9: ICC13-7 xenograft treated with D 10mg/kg Rep#3
10: ICC13-7 xenograft treated with C 10mg/kg Rep#1
11: ICC13-7 xenograft treated with C 10mg/kg Rep#2
12: ICC13-7 xenograft treated with C 10mg/kg Rep#3

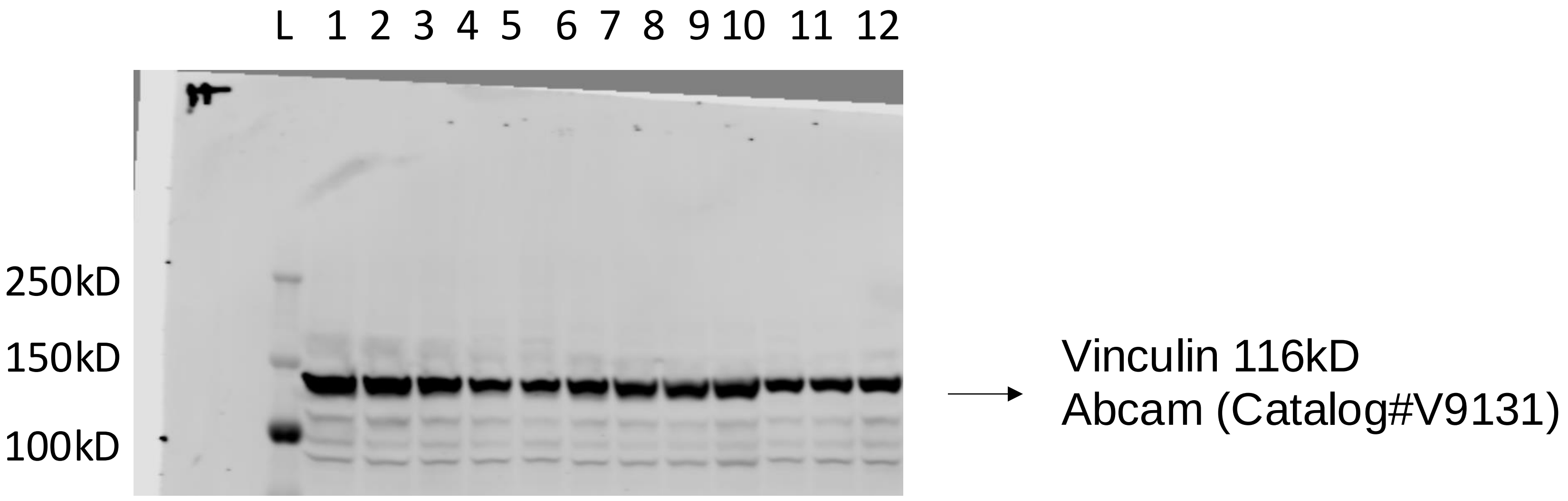

Full unedited gel for Figure 6J

1: ICC13-7 treated with IgG1 control
2: ICC13-7 treated with B/D 1uM
3: ICC13-7 treated with B/C 1uM
4: ICC13-7 treated with IgG1 control + BafA1
5: ICC13-7 treated with B/D 1uM+ BafA1
6: ICC13-7 treated with B/C 1uM + BafA1

1 2 3 4 5 6

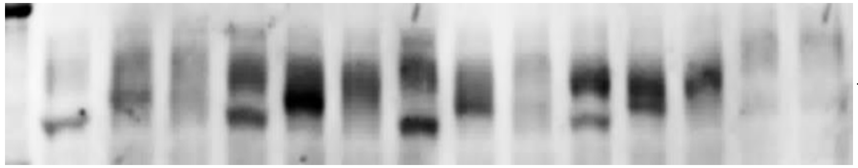

total FGFR2-OPTN (~130kD)
Antibody B/D

Full unedited gel for Figure 6J

1: ICC13-7 treated with IgG1 control
2: ICC13-7 treated with B/D 1uM
3: ICC13-7 treated with B/C 1uM
4: ICC13-7 treated with IgG1 control + BafA1
5: ICC13-7 treated with B/D 1uM+ BafA1
6: ICC13-7 treated with B/C 1uM + BafA1

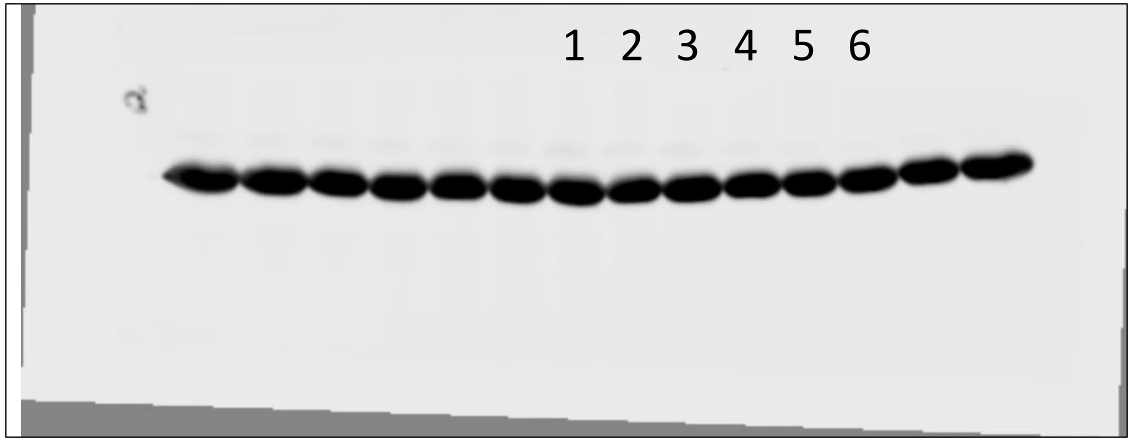

α-Tubulin, 52kD
Cell Signaling Catalog #3873

Full unedited gel for Figure 7F

0: NIH3T3: FGFR2-ACHYL1
1: NIH3T3: FGFR2-ACHYL1, V565F treated with IgG1 control
2: NIH3T3: FGFR2-ACHYL1, V565F treated with B/D
3: NIH3T3: FGFR2-ACHYL1, V565F treated with B/C
4: NIH3T3: FGFR2-ACHYL1, V565F treated with IgG1+ FGF10
5: NIH3T3: FGFR2-ACHYL1, V565F treated with B/D + FGF10
6: NIH3T3: FGFR2-ACHYL1, V565F treated with B/C +FGF10
7: NIH3T3: FGFR2-ACHYL1, V565I treated with IgG1 control
8: NIH3T3: FGFR2-ACHYL1, V565I treated with B/D
9: NIH3T3: FGFR2-ACHYL1, V565I treated with B/C
10: NIH3T3: FGFR2-ACHYL1, V565I treated with IgG1+ FGF10
11: NIH3T3: FGFR2-ACHYL1, V565I treated with B/D + FGF10
12: NIH3T3: FGFR2-ACHYL1, V565I treated with B/C +FGF10

1 2 3 4 5 6 7 8 9 10 11 12

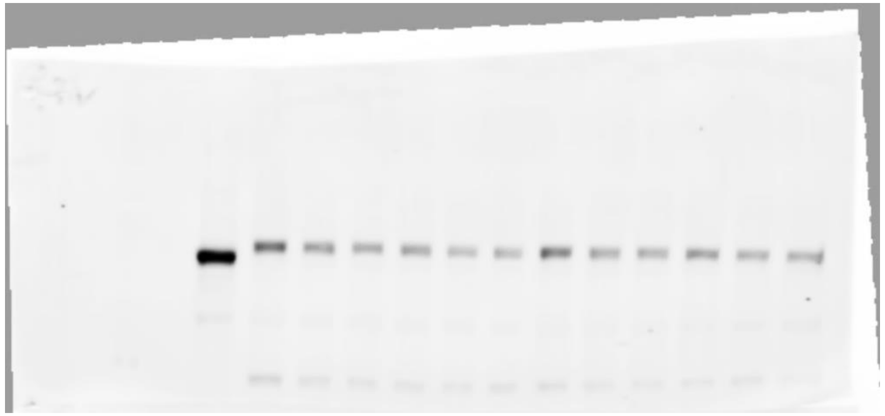

100kD

pFGFR (120, 145kD)
pFGFR (Tyr653/654)
Cell Signaling (Catalog#3471)

Full unedited gel for Figure 7F

0: NIH3T3: FGFR2-ACHYL1
1: NIH3T3: FGFR2-ACHYL1, V565F treated with IgG1 control
2: NIH3T3: FGFR2-ACHYL1, V565F treated with B/D
3: NIH3T3: FGFR2-ACHYL1, V565F treated with B/C
4: NIH3T3: FGFR2-ACHYL1, V565F treated with IgG1+ FGF10
5: NIH3T3: FGFR2-ACHYL1, V565F treated with B/D + FGF10
6: NIH3T3: FGFR2-ACHYL1, V565F treated with B/C +FGF10
7: NIH3T3: FGFR2-ACHYL1, V565I treated with IgG1 control
8: NIH3T3: FGFR2-ACHYL1, V565I treated with B/D
9: NIH3T3: FGFR2-ACHYL1, V565I treated with B/C
10: NIH3T3: FGFR2-ACHYL1, V565I treated with IgG1+ FGF10
11: NIH3T3: FGFR2-ACHYL1, V565I treated with B/D + FGF10
12: NIH3T3: FGFR2-ACHYL1, V565I treated with B/C +FGF10

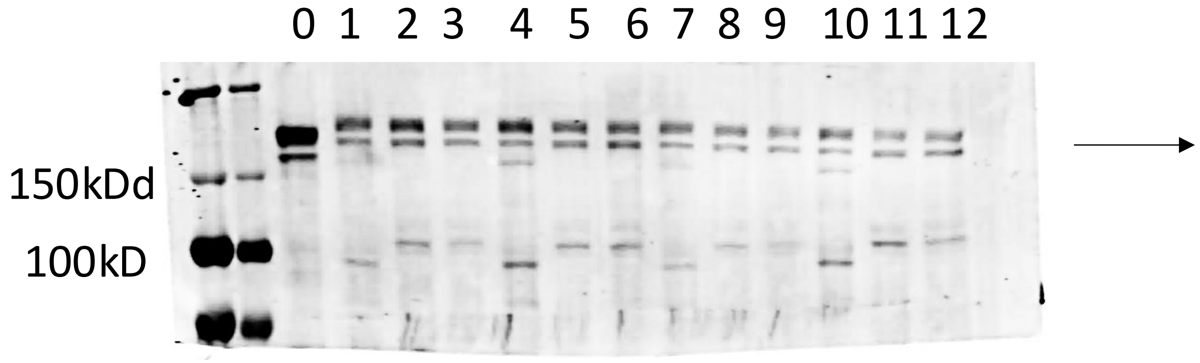

total FGFR2 (~130kD)
Antibody B/D

Full unedited gel for Figure 7F

0: NIH3T3 parental
1: NIH3T3: FGFR2-ACHYL1, V565F treated with IgG1 control
2: NIH3T3: FGFR2-ACHYL1, V565F treated with B/D
3: NIH3T3: FGFR2-ACHYL1, V565F treated with B/C
4: NIH3T3: FGFR2-ACHYL1, V565F treated with IgG1+ FGF10
5: NIH3T3: FGFR2-ACHYL1, V565F treated with B/D + FGF10
6: NIH3T3: FGFR2-ACHYL1, V565F treated with B/C +FGF10
7: NIH3T3: FGFR2-ACHYL1, V565I treated with IgG1 control
8: NIH3T3: FGFR2-ACHYL1, V565I treated with B/D
9: NIH3T3: FGFR2-ACHYL1, V565I treated with B/C
10: NIH3T3: FGFR2-ACHYL1, V565I treated with IgG1+ FGF10
11: NIH3T3: FGFR2-ACHYL1, V565I treated with B/D + FGF10
12: NIH3T3: FGFR2-ACHYL1, V565I treated with B/C +FGF10

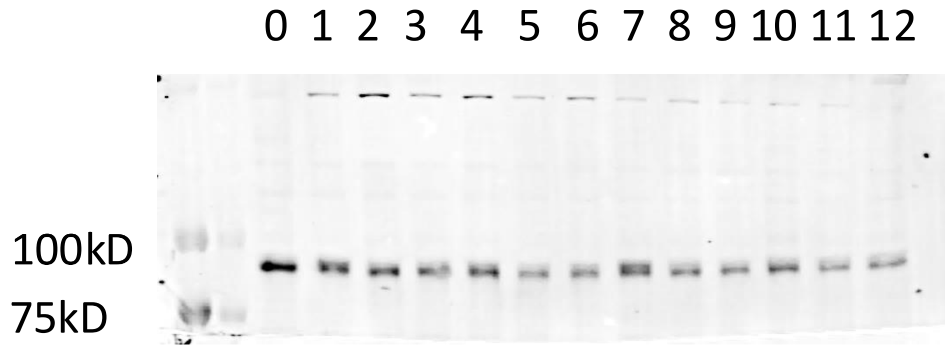

pFRS2 80-85kD
pFRS2(Y436)
Cell Signaling (Catalog#3861)

Full unedited gel for Figure 7F

0: NIH3T3 parental
1: NIH3T3: FGFR2-ACHYL1, V565F treated with IgG1 control
2: NIH3T3: FGFR2-ACHYL1, V565F treated with B/D
3: NIH3T3: FGFR2-ACHYL1, V565F treated with B/C
4: NIH3T3: FGFR2-ACHYL1, V565F treated with IgG1+ FGF10
5: NIH3T3: FGFR2-ACHYL1, V565F treated with B/D + FGF10
6: NIH3T3: FGFR2-ACHYL1, V565F treated with B/C +FGF10
7: NIH3T3: FGFR2-ACHYL1, V565I treated with IgG1 control
8: NIH3T3: FGFR2-ACHYL1, V565I treated with B/D
9: NIH3T3: FGFR2-ACHYL1, V565I treated with B/C
10: NIH3T3: FGFR2-ACHYL1, V565I treated with IgG1+ FGF10
11: NIH3T3: FGFR2-ACHYL1, V565I treated with B/D + FGF10
12: NIH3T3: FGFR2-ACHYL1, V565I treated with B/C +FGF10

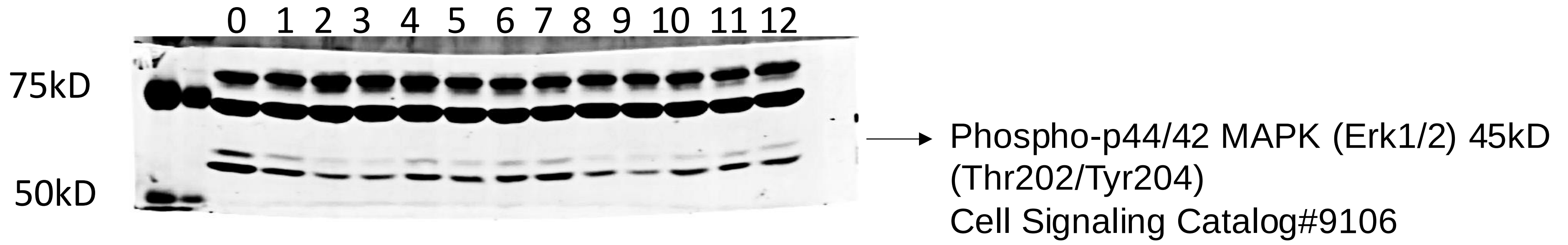

Full unedited gel for Figure 7F

0: NIH3T3 parental
1: NIH3T3: FGFR2-ACHYL1, V565F treated with IgG1 control
2: NIH3T3: FGFR2-ACHYL1, V565F treated with B/D
3: NIH3T3: FGFR2-ACHYL1, V565F treated with B/C
4: NIH3T3: FGFR2-ACHYL1, V565F treated with IgG1+ FGF10
5: NIH3T3: FGFR2-ACHYL1, V565F treated with B/D + FGF10
6: NIH3T3: FGFR2-ACHYL1, V565F treated with B/C +FGF10
7: NIH3T3: FGFR2-ACHYL1, V565I treated with IgG1 control
8: NIH3T3: FGFR2-ACHYL1, V565I treated with B/D
9: NIH3T3: FGFR2-ACHYL1, V565I treated with B/C
10: NIH3T3: FGFR2-ACHYL1, V565I treated with IgG1+ FGF10
11: NIH3T3: FGFR2-ACHYL1, V565I treated with B/D + FGF10
12: NIH3T3: FGFR2-ACHYL1, V565I treated with B/C +FGF10

0 1 2 3 4 5 6 7 8 9 10 11 12

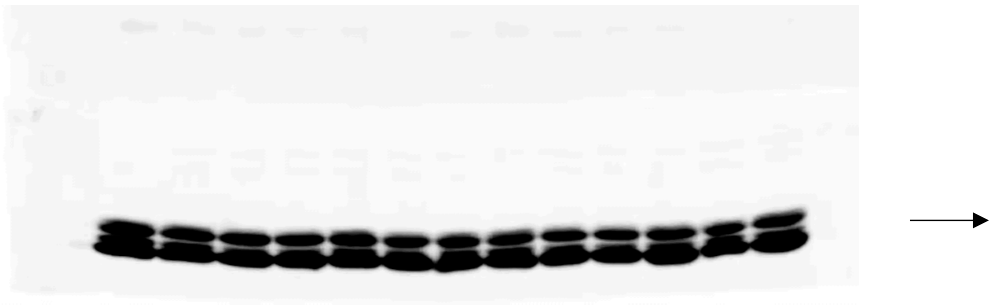

p44/42 MAPK (Erk1/2) 42, 44kD
Cell Signaling Catalog#4695

Full unedited gel for Figure 7F

0: NIH3T3 parental
1: NIH3T3: FGFR2-ACHYL1, V565F treated with IgG1 control
2: NIH3T3: FGFR2-ACHYL1, V565F treated with B/D
3: NIH3T3: FGFR2-ACHYL1, V565F treated with B/C
4: NIH3T3: FGFR2-ACHYL1, V565F treated with IgG1+ FGF10
5: NIH3T3: FGFR2-ACHYL1, V565F treated with B/D + FGF10
6: NIH3T3: FGFR2-ACHYL1, V565F treated with B/C +FGF10
7: NIH3T3: FGFR2-ACHYL1, V565I treated with IgG1 control
8: NIH3T3: FGFR2-ACHYL1, V565I treated with B/D
9: NIH3T3: FGFR2-ACHYL1, V565I treated with B/C
10: NIH3T3: FGFR2-ACHYL1, V565I treated with IgG1+ FGF10
11: NIH3T3: FGFR2-ACHYL1, V565I treated with B/D + FGF10
12: NIH3T3: FGFR2-ACHYL1, V565I treated with B/C +FGF10

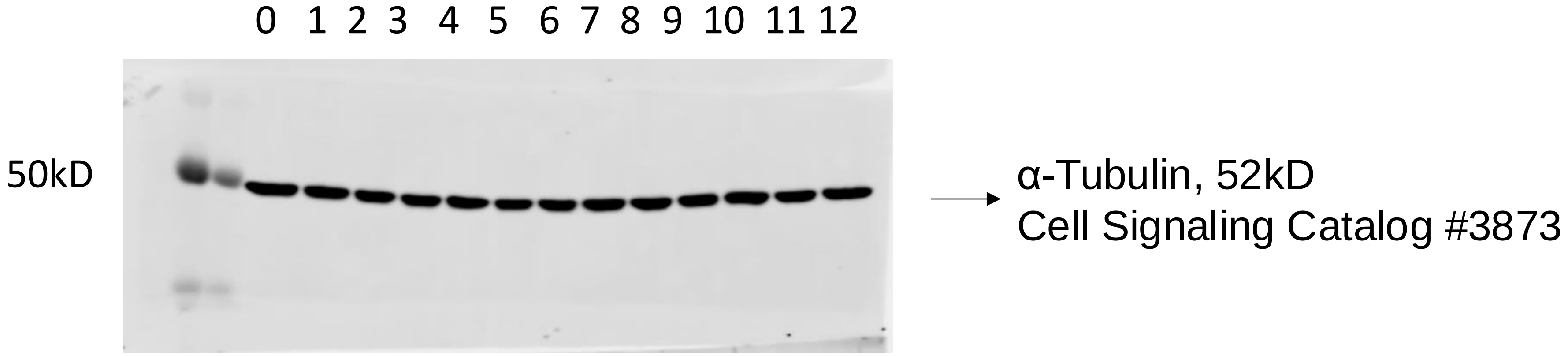

Full unedited gel for Figure 7J

1: CCLP1 parental non treated
2: CCLP1 parental treated with BGJ398
3: CCLP1: FGFR2-PHGDH (V565F) treated with IgG1 con
4: CCLP1: FGFR2-PHGDH (V565F) treated with BGJ398
5: CCLP1: FGFR2-PHGDH (V565F) treated with B/D+BGJ
6: CCLP1: FGFR2-PHGDH (V565F) treated with B/C+BGJ
7: CCLP1: FGFR2-PHGDH (V565F) treated with B/D
8: CCLP1: FGFR2-PHGDH (V565F) treated with B/C

1 2 3 4 5 6 7 8

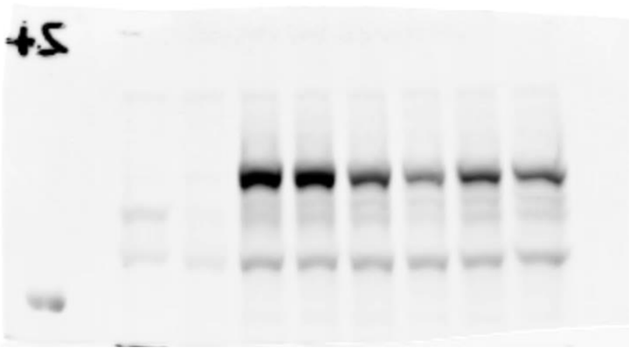

→ pFGFR (120, 145kD)
pFGFR (Tyr653/654)
Cell Signaling (Catalog#3471)

Full unedited gel for Figure 7J

1: CCLP1 parental non treated
2: CCLP1 parental treated with BGJ398
3: CCLP1: FGFR2-PHGDH (V565F) treated with IgG1 con
4: CCLP1: FGFR2-PHGDH (V565F) treated with BGJ398
5: CCLP1: FGFR2-PHGDH (V565F) treated with B/D+BGJ
6: CCLP1: FGFR2-PHGDH (V565F) treated with B/C+BGJ
7: CCLP1: FGFR2-PHGDH (V565F) treated with B/D
8: CCLP1: FGFR2-PHGDH (V565F) treated with B/C

1 2 3 4 5 6 7 8

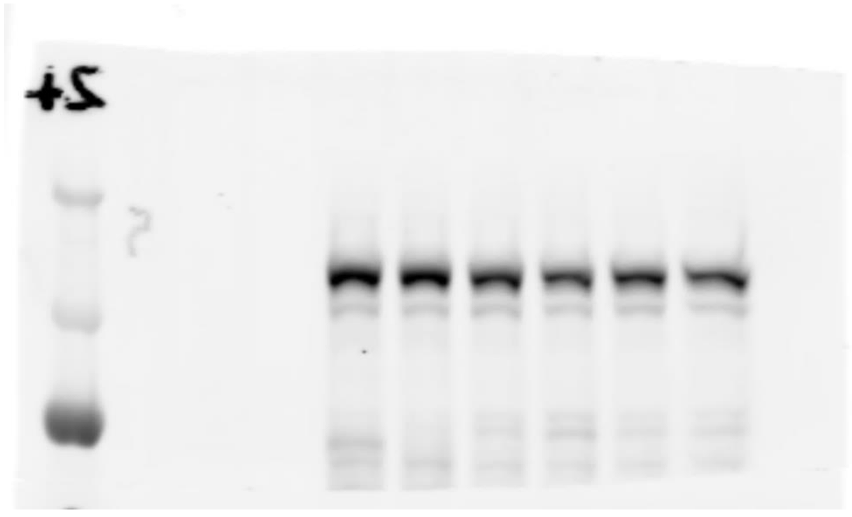

total FGFR2 (~130kD)
Antibody B/D

Full unedited gel for Figure 7J

1: CCLP1 parental non treated
2: CCLP1 parental treated with BGJ398
3: CCLP1: FGFR2-PHGDH (V565F) treated with IgG1 con
4: CCLP1: FGFR2-PHGDH (V565F) treated with BGJ398
5: CCLP1: FGFR2-PHGDH (V565F) treated with B/D+BGJ
6: CCLP1: FGFR2-PHGDH (V565F) treated with B/C+BGJ
7: CCLP1: FGFR2-PHGDH (V565F) treated with B/D
8: CCLP1: FGFR2-PHGDH (V565F) treated with B/C

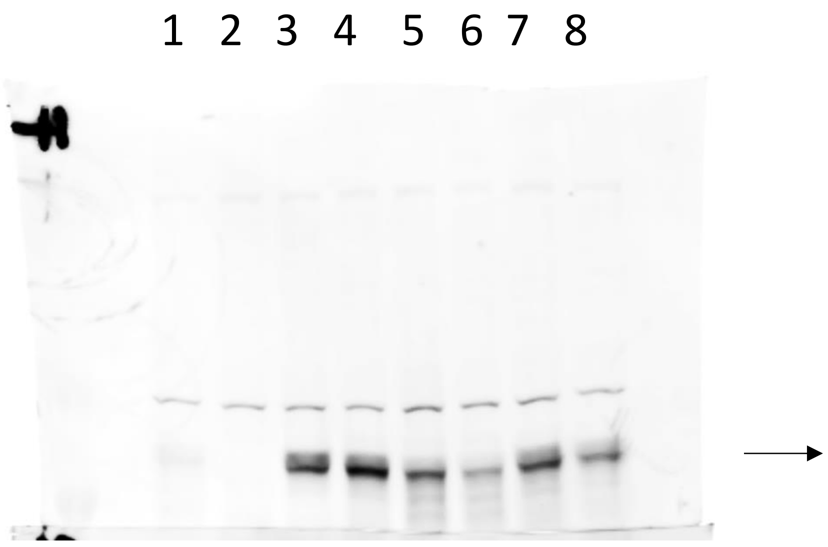

pFRS2 80-85kD
pFRS2(Y436)
Cell Signaling (Catalog#3861)

Full unedited gel for Figure 7J

1: CCLP1 parental non treated
2: CCLP1 parental treated with BGJ398
3: CCLP1: FGFR2-PHGDH (V565F) treated with IgG1 con
4: CCLP1: FGFR2-PHGDH (V565F) treated with BGJ398
5: CCLP1: FGFR2-PHGDH (V565F) treated with B/D+BGJ
6: CCLP1: FGFR2-PHGDH (V565F) treated with B/C+BGJ
7: CCLP1: FGFR2-PHGDH (V565F) treated with B/D
8: CCLP1: FGFR2-PHGDH (V565F) treated with B/C

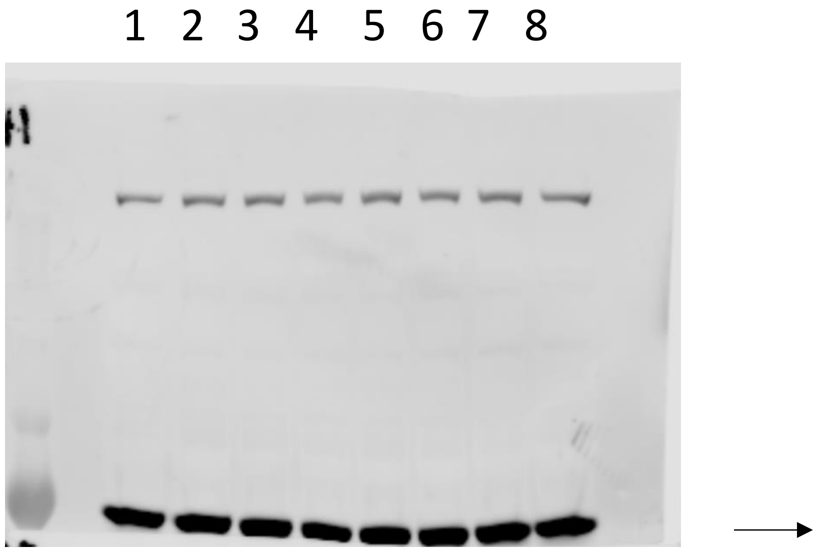

tFRS2 80-85kD
FRS2(Abcam, Catalog#ab183492)

Full unedited gel for Figure 7J

1: CCLP1 parental non treated
2: CCLP1 parental treated with BGJ398
3: CCLP1: FGFR2-PHGDH (V565F) treated with IgG1 con
4: CCLP1: FGFR2-PHGDH (V565F) treated with BGJ398
5: CCLP1: FGFR2-PHGDH (V565F) treated with B/D+BGJ
6: CCLP1: FGFR2-PHGDH (V565F) treated with B/C+BGJ
7: CCLP1: FGFR2-PHGDH (V565F) treated with B/D
8: CCLP1: FGFR2-PHGDH (V565F) treated with B/C

1 2 3 4 5 6 7 8

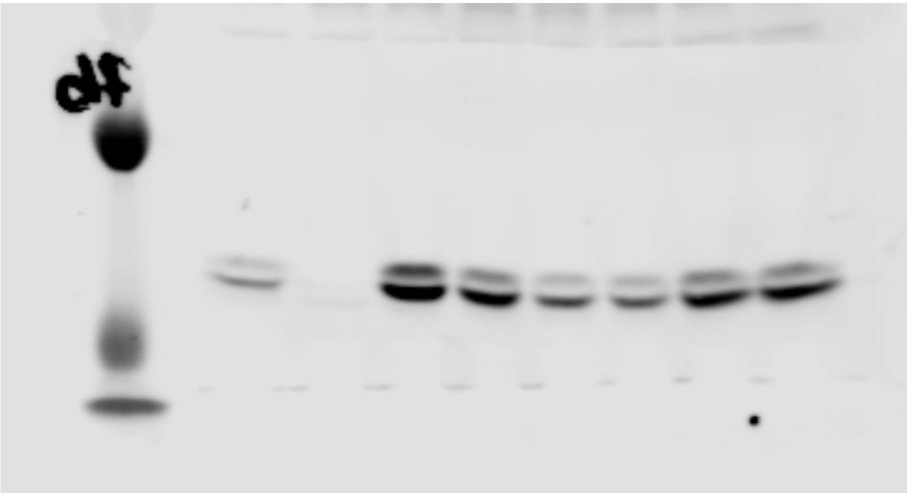

Phospho-p44/42 MAPK (Erk1/2) 45kD
(Thr202/Tyr204)
Cell Signaling Catalog#9106

Full unedited gel for Figure 7J

1: CCLP1 parental non treated
2: CCLP1 parental treated with BGJ398
3: CCLP1: FGFR2-PHGDH (V565F) treated with IgG1 con
4: CCLP1: FGFR2-PHGDH (V565F) treated with BGJ398
5: CCLP1: FGFR2-PHGDH (V565F) treated with B/D+BGJ
6: CCLP1: FGFR2-PHGDH (V565F) treated with B/C+BGJ
7: CCLP1: FGFR2-PHGDH (V565F) treated with B/D
8: CCLP1: FGFR2-PHGDH (V565F) treated with B/C

1 2 3 4 5 6 7 8

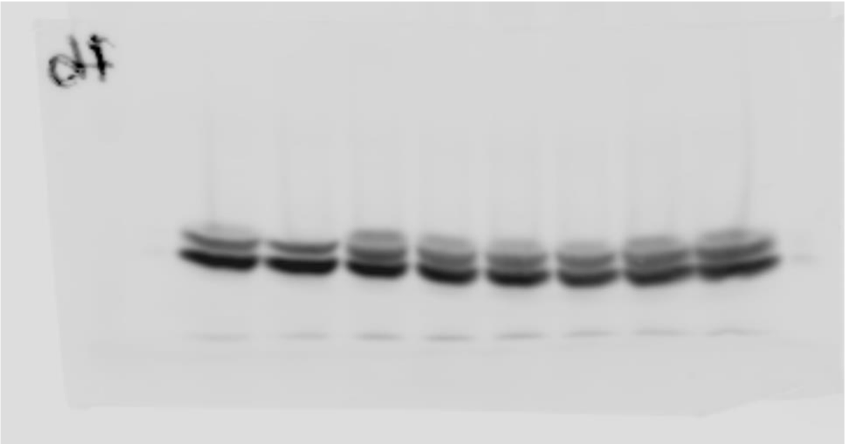

p44/42 MAPK (Erk1/2) 42, 44kD
Cell Signaling Catalog#4695

Full unedited gel for Figure 7J

1: CCLP1 parental non treated
2: CCLP1 parental treated with BGJ398
3: CCLP1: FGFR2-PHGDH (V565F) treated with IgG1 con
4: CCLP1: FGFR2-PHGDH (V565F) treated with BGJ398
5: CCLP1: FGFR2-PHGDH (V565F) treated with B/D+BGJ
6: CCLP1: FGFR2-PHGDH (V565F) treated with B/C+BGJ
7: CCLP1: FGFR2-PHGDH (V565F) treated with B/D
8: CCLP1: FGFR2-PHGDH (V565F) treated with B/C

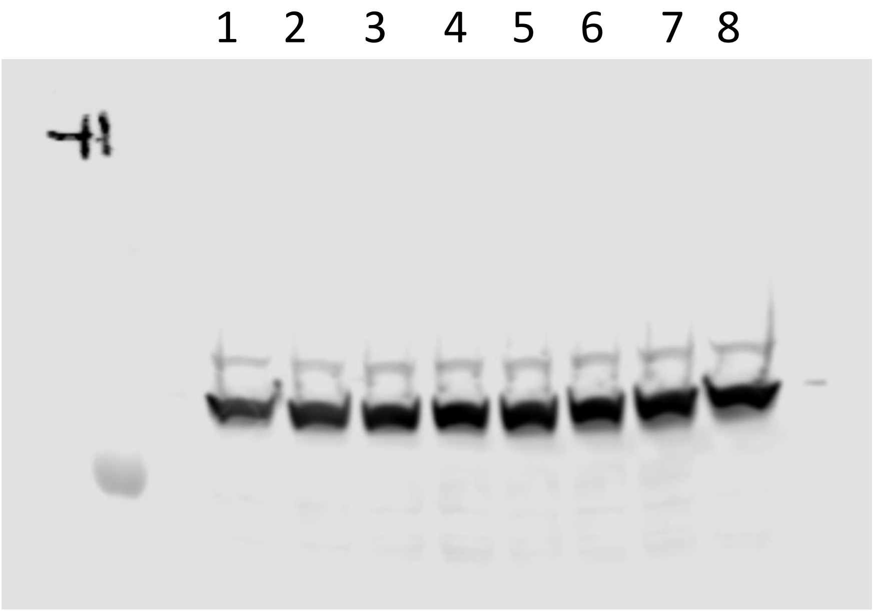

Vinculin 116kD
Abcam (Catalog#V9131)

Full unedited gel for Figure 7M

1: NIH3T3 parental
2: NIH3T3: FGFR2 (H167_N173Del) treated with IgG1 con
3: NIH3T3: FGFR2 (H167_N173Del) treated with B/D
4: NIH3T3: FGFR2 (H167_N173Del) treated with B/C
5: NIH3T3: FGFR2 (H167_N173Del) treated with B
6: NIH3T3: FGFR2 (H167_N173Del) treated with D
7: NIH3T3: FGFR2 (H167_N173Del) treated with C

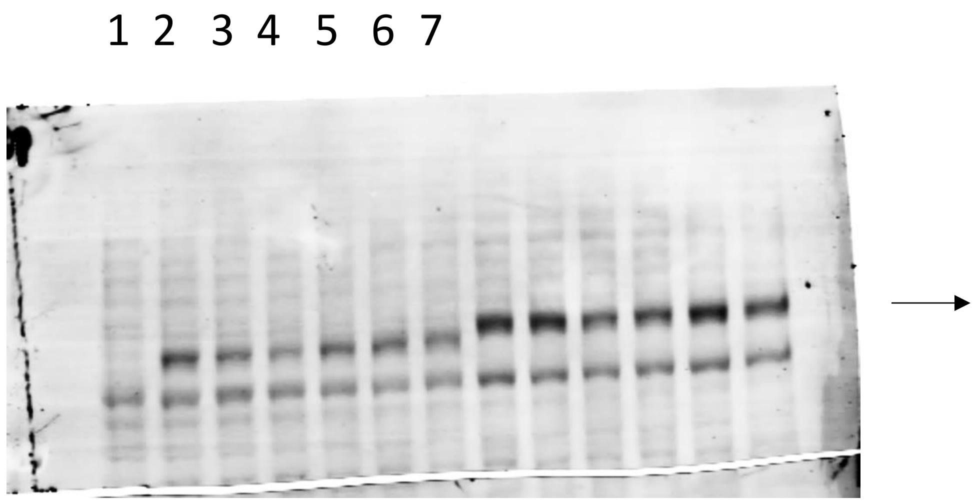

pFGFR (120, 145kD)
pFGFR (Tyr653/654)
Cell Signaling (Catalog#3471)

Full unedited gel for Figure 7M

1: NIH3T3 parental
2: NIH3T3: FGFR2 (H167_N173Del) treated with IgG1 con
3: NIH3T3: FGFR2 (H167_N173Del) treated with B/D
4: NIH3T3: FGFR2 (H167_N173Del) treated with B/C
5: NIH3T3: FGFR2 (H167_N173Del) treated with B
6: NIH3T3: FGFR2 (H167_N173Del) treated with D
7: NIH3T3: FGFR2 (H167_N173Del) treated with C

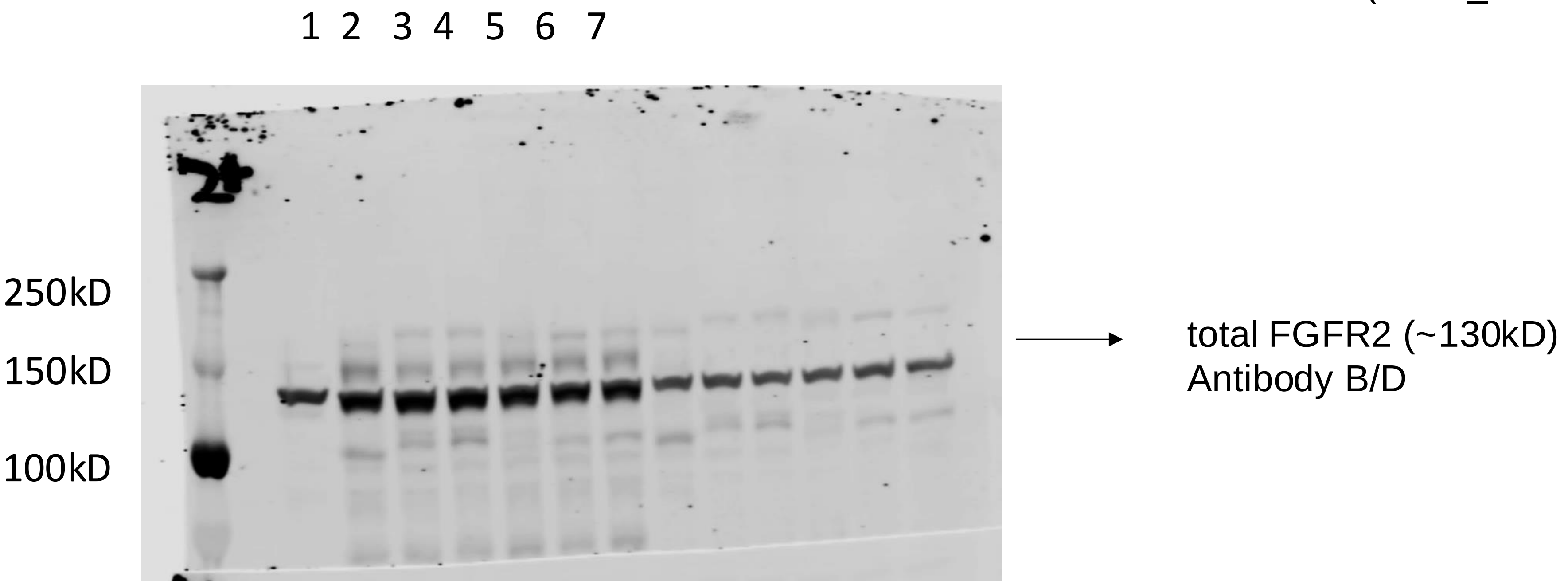

Full unedited gel for Figure 7M

1: NIH3T3 parental
2: NIH3T3: FGFR2 (H167_N173Del) treated with IgG1 con
3: NIH3T3: FGFR2 (H167_N173Del) treated with B/D
4: NIH3T3: FGFR2 (H167_N173Del) treated with B/C
5: NIH3T3: FGFR2 (H167_N173Del) treated with B
6: NIH3T3: FGFR2 (H167_N173Del) treated with D
7: NIH3T3: FGFR2 (H167_N173Del) treated with C

1 2 3 4 5 6 7

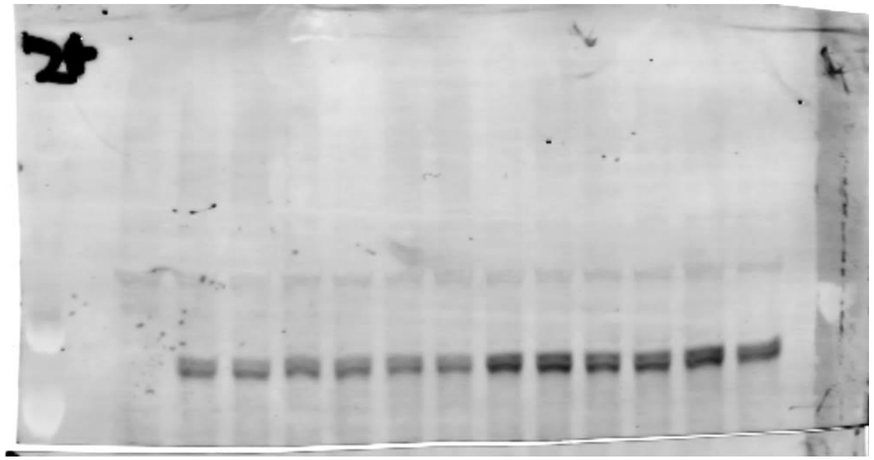

→ pFRS2 80-85kD
pFRS2(Y436)
Cell Signaling (Catalog#3861)

Full unedited gel for Figure 7M

1: NIH3T3 parental
2: NIH3T3: FGFR2 (H167_N173Del) treated with IgG1 con
3: NIH3T3: FGFR2 (H167_N173Del) treated with B/D
4: NIH3T3: FGFR2 (H167_N173Del) treated with B/C
5: NIH3T3: FGFR2 (H167_N173Del) treated with B
6: NIH3T3: FGFR2 (H167_N173Del) treated with D
7: NIH3T3: FGFR2 (H167_N173Del) treated with C

1 2 3 4 5 6 7

75kD

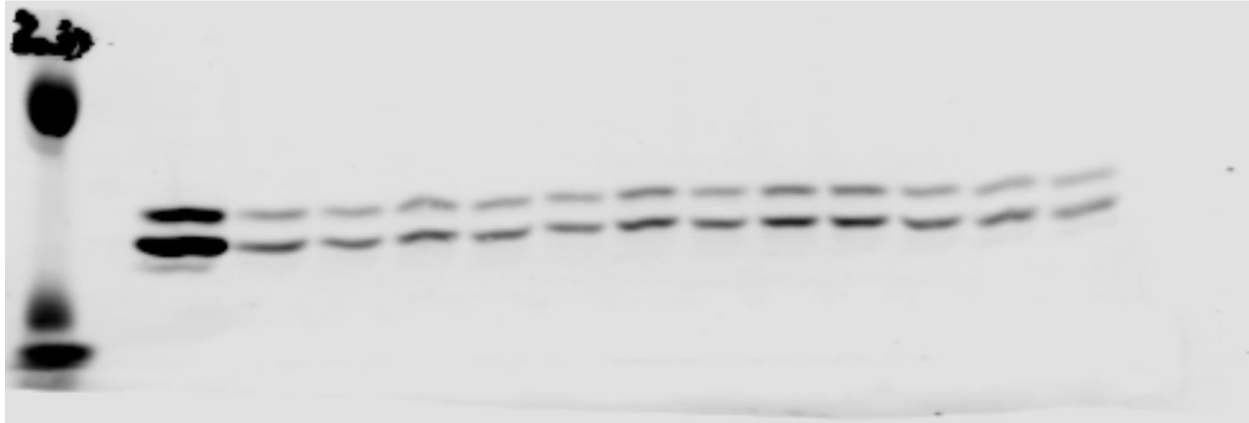

Phospho-p44/42 MAPK (Erk1/2) 45kD (Thr202/Tyr204)
Cell Signaling Catalog#9106

Full unedited gel for Figure 7M

1: NIH3T3 parental
2: NIH3T3: FGFR2 (H167_N173Del) treated with IgG1 con
3: NIH3T3: FGFR2 (H167_N173Del) treated with B/D
4: NIH3T3: FGFR2 (H167_N173Del) treated with B/C
5: NIH3T3: FGFR2 (H167_N173Del) treated with B
6: NIH3T3: FGFR2 (H167_N173Del) treated with D
7: NIH3T3: FGFR2 (H167_N173Del) treated with C

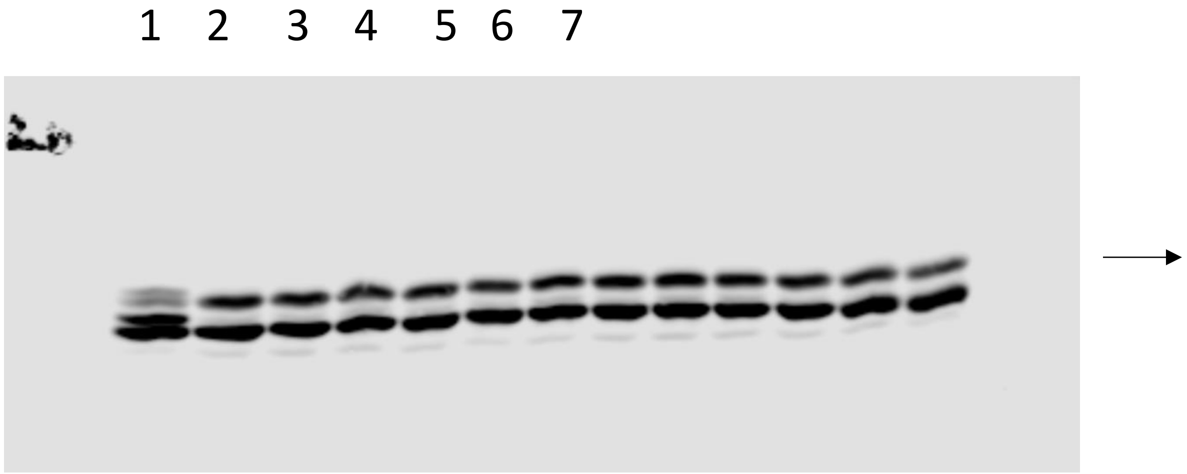

p44/42 MAPK (Erk1/2) 42, 44kD
Cell Signaling Catalog#4695

Full unedited gel for Figure 7M

1: NIH3T3 parental
2: NIH3T3: FGFR2 (H167_N173Del) treated with IgG1 con
3: NIH3T3: FGFR2 (H167_N173Del) treated with B/D
4: NIH3T3: FGFR2 (H167_N173Del) treated with B/C
5: NIH3T3: FGFR2 (H167_N173Del) treated with B
6: NIH3T3: FGFR2 (H167_N173Del) treated with D
7: NIH3T3: FGFR2 (H167_N173Del) treated with C

1 2 3 4 5 6 7

250kD
150kD
100kD

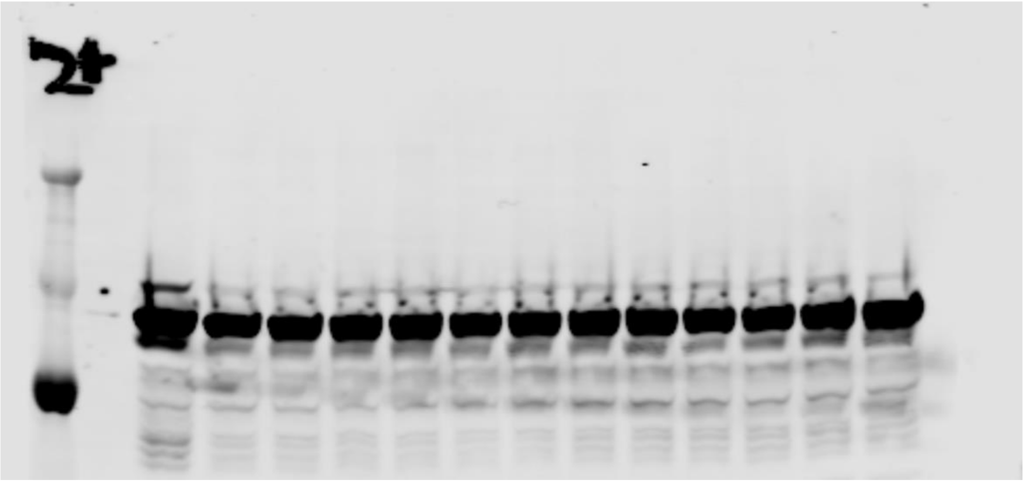

Vinculin 116kD
Abcam (Catalog#V9131)

Full unedited gel for Figure S7C

1: CCLP1 parental
2: CCLP1: FGFR2-PHGDH
3: CCLP1: FGFR2-PGHDH (V565F)

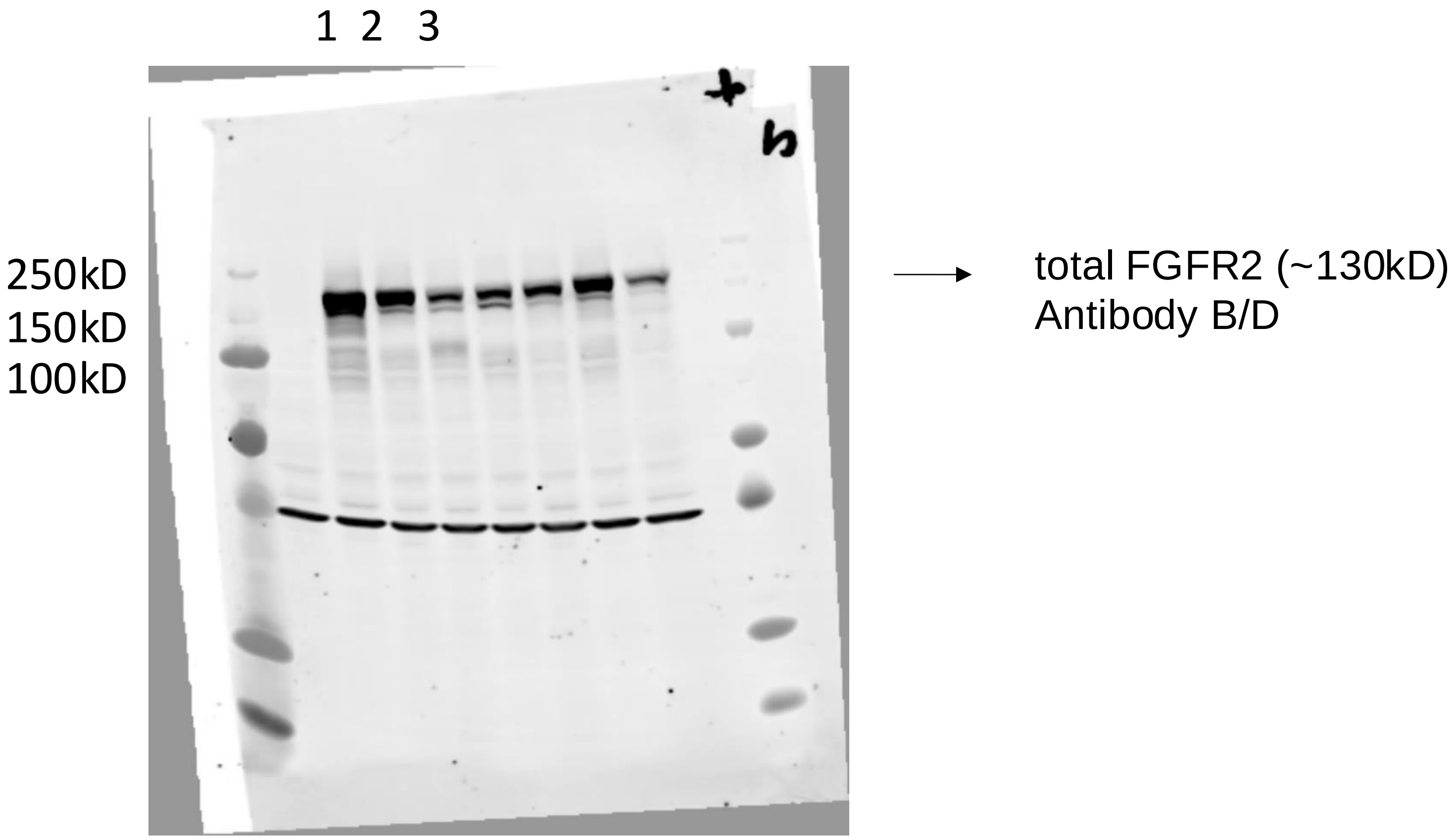

Full unedited gel for Figure S7C

1: CCLP1 parental
2: CCLP1: FGFR2-PHGDH
3: CCLP1: FGFR2-PGHDH (V565F)

1 2 3

250kD
150kD
100kD

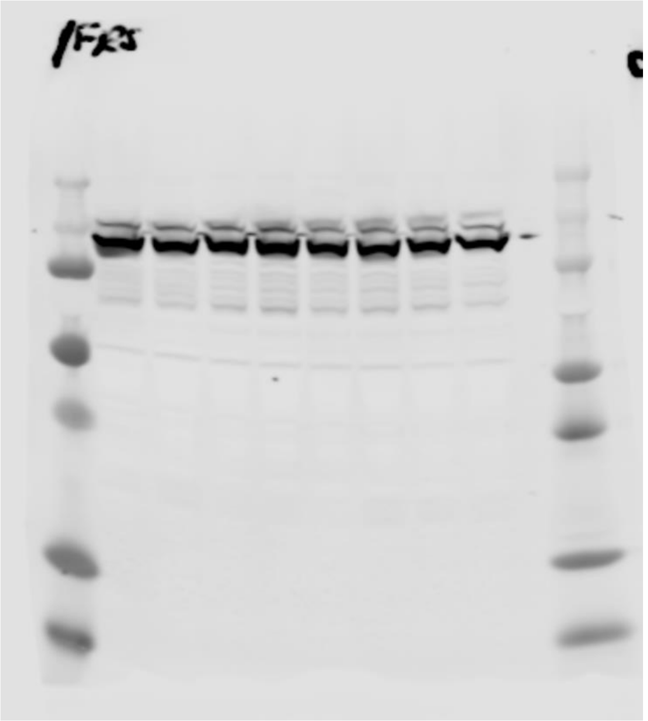

Vinculin 116kD
Abcam (Catalog#V9131)

Full unedited gel for Figure S7G

1: NIH3T3 parental
2: NIH3T3: FGFR2 WT
3: NIH3T3: FGFR2 with mutation as Patient 1
4: NIH3T3: FGFR2 with mutation as Patient 2
5: NIH3T3: FGFR2 with mutation as Patient 3
6: NIH3T3: FGFR2 with mutation as Patient 4

1 2 3 4 5 6

150kD
100kD

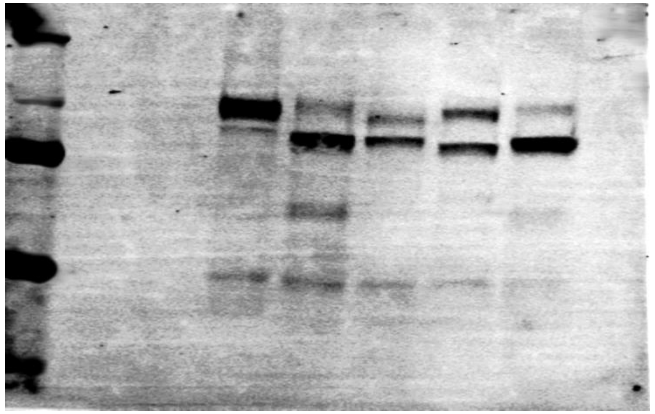

total FGFR2 (~130kD)
Antibody E

Full unedited gel for Figure S7G

1: NIH3T3 parental
2: NIH3T3: FGFR2 WT
3: NIH3T3: FGFR2 with mutation as Patient 1
4: NIH3T3: FGFR2 with mutation as Patient 2
5: NIH3T3: FGFR2 with mutation as Patient 3
6: NIH3T3: FGFR2 with mutation as Patient 4

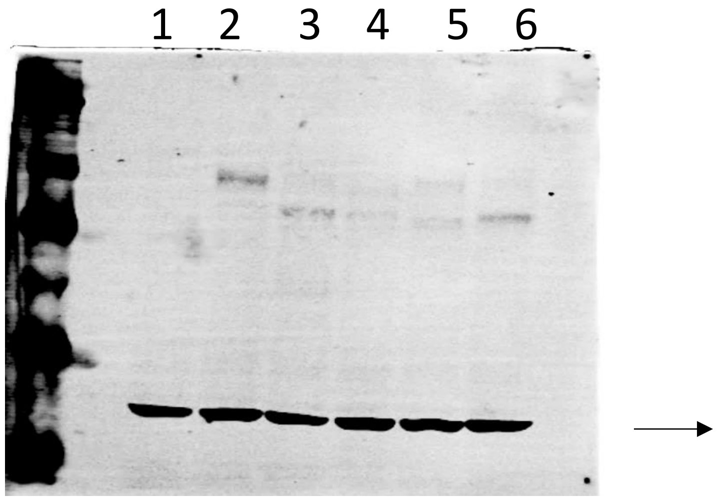

α-Tubulin, 52kD
Cell Signaling Catalog #3873

Full unedited gel for Figure S7L

1: NIH3T3: FGFR2 with mutation as Patient 1
2: NIH3T3: FGFR2 with mutation as Patient 1 + BGJ398 (Infigratinib)
3: NIH3T3: FGFR2 with mutation as Patient 2
4: NIH3T3: FGFR2 with mutation as Patient 2 + BGJ398 (Infigratinib)
5: NIH3T3: FGFR2 with mutation as Patient 3
6: NIH3T3: FGFR2 with mutation as Patient 3 + BGJ398 (Infigratinib)
7: NIH3T3: FGFR2 with mutation as Patient 4
8: NIH3T3: FGFR2 with mutation as Patient 4 + BGJ398 (Infigratinib)

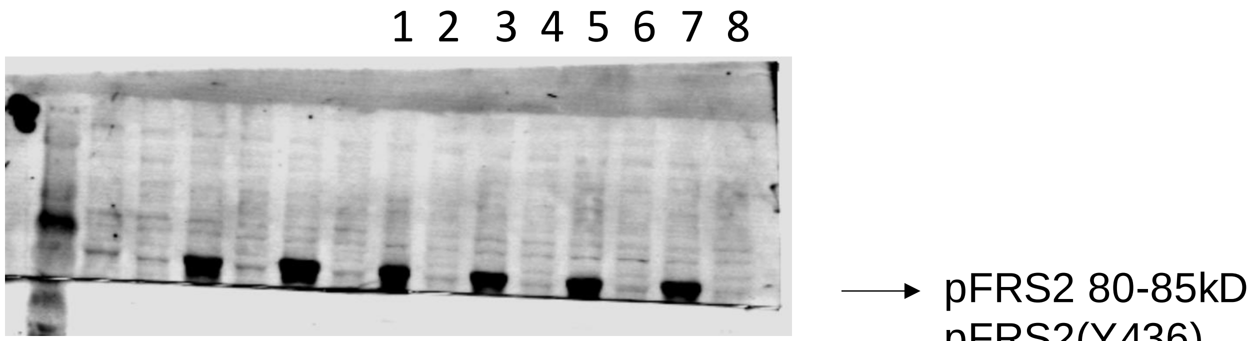

pFRS2(Y436)
Cell Signaling (Catalog#3861)

Full unedited gel for Figure S7L

1: NIH3T3: FGFR2 with mutation as Patient 1
2: NIH3T3: FGFR2 with mutation as Patient 1 + BGJ398 (Infigratinib)
3: NIH3T3: FGFR2 with mutation as Patient 2
4: NIH3T3: FGFR2 with mutation as Patient 2 + BGJ398 (Infigratinib)
5: NIH3T3: FGFR2 with mutation as Patient 3
6: NIH3T3: FGFR2 with mutation as Patient 3 + BGJ398 (Infigratinib)
7: NIH3T3: FGFR2 with mutation as Patient 4
8: NIH3T3: FGFR2 with mutation as Patient 4 + BGJ398 (Infigratinib)

1 2 3 4 5 6 7 8

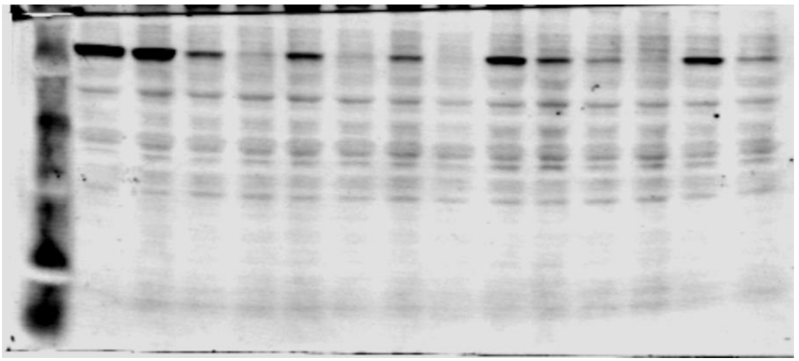

Phospho-Akt (Ser473), 60kD
Cell Signaling Catalog #4060

Full unedited gel for Figure S7L

1: NIH3T3: FGFR2 with mutation as Patient 1
2: NIH3T3: FGFR2 with mutation as Patient 1 + BGJ398 (Infigratinib)
3: NIH3T3: FGFR2 with mutation as Patient 2
4: NIH3T3: FGFR2 with mutation as Patient 2 + BGJ398 (Infigratinib)
5: NIH3T3: FGFR2 with mutation as Patient 3
6: NIH3T3: FGFR2 with mutation as Patient 3 + BGJ398 (Infigratinib)
7: NIH3T3: FGFR2 with mutation as Patient 4
8: NIH3T3: FGFR2 with mutation as Patient 4 + BGJ398 (Infigratinib)

1 2 3 4 5 6 7 8

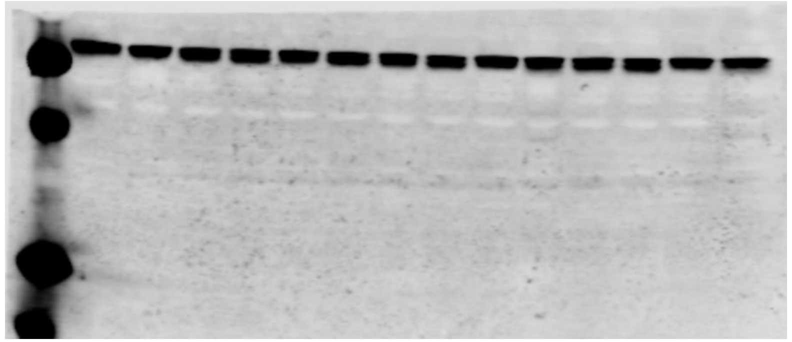

Akt (pan), 60kD
Cell Signaling Catalog #2920

Full unedited gel for Figure S7L

1: NIH3T3: FGFR2 with mutation as Patient 1
2: NIH3T3: FGFR2 with mutation as Patient 1 + BGJ398 (Infigratinib)
3: NIH3T3: FGFR2 with mutation as Patient 2
4: NIH3T3: FGFR2 with mutation as Patient 2 + BGJ398 (Infigratinib)
5: NIH3T3: FGFR2 with mutation as Patient 3
6: NIH3T3: FGFR2 with mutation as Patient 3 + BGJ398 (Infigratinib)
7: NIH3T3: FGFR2 with mutation as Patient 4
8: NIH3T3: FGFR2 with mutation as Patient 4 + BGJ398 (Infigratinib)

1 2 3 4 5 6 7 8

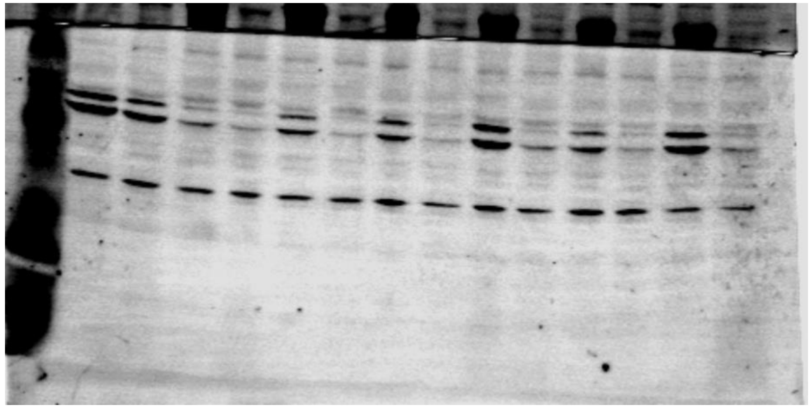

Phospho-p44/42 MAPK (Erk1/2) 45kD
(Thr202/Tyr204)
Cell Signaling Catalog#9106

Full unedited gel for Figure S7L

1: NIH3T3: FGFR2 with mutation as Patient 1
2: NIH3T3: FGFR2 with mutation as Patient 1 + BGJ398 (Infigratinib)
3: NIH3T3: FGFR2 with mutation as Patient 2
4: NIH3T3: FGFR2 with mutation as Patient 2 + BGJ398 (Infigratinib)
5: NIH3T3: FGFR2 with mutation as Patient 3
6: NIH3T3: FGFR2 with mutation as Patient 3 + BGJ398 (Infigratinib)
7: NIH3T3: FGFR2 with mutation as Patient 4
8: NIH3T3: FGFR2 with mutation as Patient 4 + BGJ398 (Infigratinib)

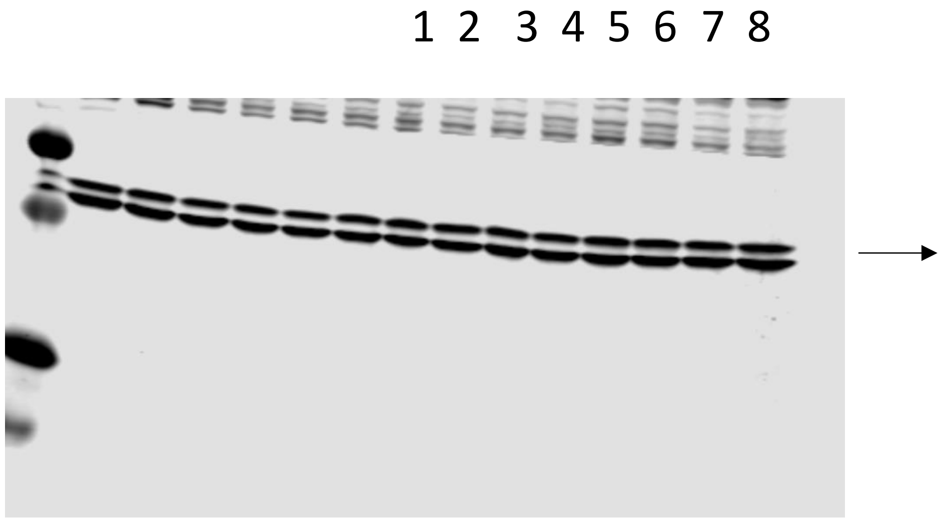

Full unedited gel for Figure S7L

1: NIH3T3: FGFR2 with mutation as Patient 1
2: NIH3T3: FGFR2 with mutation as Patient 1 + BGJ398 (Infigratinib)
3: NIH3T3: FGFR2 with mutation as Patient 2
4: NIH3T3: FGFR2 with mutation as Patient 2 + BGJ398 (Infigratinib)
5: NIH3T3: FGFR2 with mutation as Patient 3
6: NIH3T3: FGFR2 with mutation as Patient 3 + BGJ398 (Infigratinib)
7: NIH3T3: FGFR2 with mutation as Patient 4
8: NIH3T3: FGFR2 with mutation as Patient 4 + BGJ398 (Infigratinib)

1 2 3 4 5 6 7 8

250kD
150kD
100kD

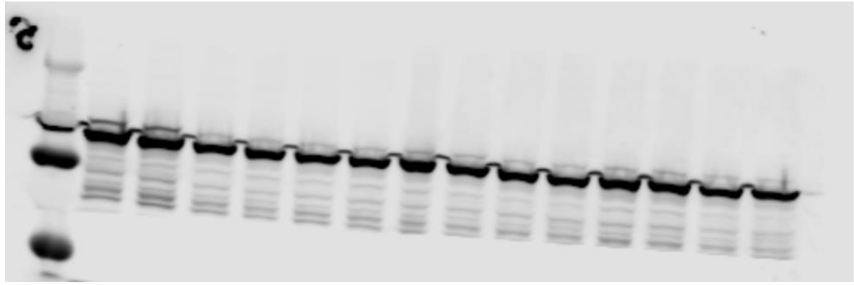

Vinculin 116kD
Abcam (Catalog#V9131)
